# TEMP: a computational method for analyzing transposable element polymorphism in populations

**DOI:** 10.1093/nar/gku323

**Published:** 2014-04-21

**Authors:** Jiali Zhuang, Jie Wang, William Theurkauf, Zhiping Weng

**Affiliations:** 1 Program in Bioinformatics and Integrative Biology, Department of Biochemistry and Molecular Pharmacology; 2 Program in Cell and Developmental Dynamics; 3 Program in Molecular Medicine, and University of Massachusetts Medical School, Worcester, MA 01605, USA

## Abstract

Insertions and excisions of transposable elements (TEs) affect both the stability and variability of the genome. Studying the dynamics of transposition at the population level can provide crucial insights into the processes and mechanisms of genome evolution. Pooling genomic materials from multiple individuals followed by high-throughput sequencing is an efficient way of characterizing genomic polymorphisms in a population. Here we describe a novel method named TEMP, specifically designed to detect TE movements present with a wide range of frequencies in a population. By combining the information provided by pair-end reads and split reads, TEMP is able to identify both the presence and absence of TE insertions in genomic DNA sequences derived from heterogeneous samples; accurately estimate the frequencies of transposition events in the population and pinpoint junctions of high frequency transposition events at nucleotide resolution. Simulation data indicate that TEMP outperforms other algorithms such as PoPoolationTE, RetroSeq, VariationHunter and GASVPro. TEMP also performs well on whole-genome human data derived from the 1000 Genomes Project. We applied TEMP to characterize the TE frequencies in a wild *Drosophila melanogaster* population and study the inheritance patterns of TEs during hybrid dysgenesis. We also identified sequence signatures of TE insertion and possible molecular effects of TE movements, such as altered gene expression and piRNA production. TEMP is freely available at github: https://github.com/JialiUMassWengLab/TEMP.git.

## INTRODUCTION

Transposable element (TE) mobilization is one of the major sources of genomic variation and a potential driving force of evolution (1–3). Detecting transposition events within the genome is therefore crucial for understanding the mechanisms by which TEs are regulated and the phenotypic consequences that result from TE movements. The task of detecting TE insertions and excisions falls within the more general category of genomic structural variation detection (4). Much progress has been made in discovering structural variations from high-throughput genomic DNA sequencing data (5–7). So far, most structural variation discovery tools are designed to handle isogenic samples—i.e. they assume that the sequence reads originate from a single genome or at least the sample is dominated by a single genome (4). However, just as any other types of genomic variation, it would be extremely useful to estimate the population frequency of polymorphic transposition events. Sequencing a large number of individuals in a population separately is impossible under many circumstances because of the prohibitively high costs and the difficulty in obtaining enough experimental material. Pooled sequencing is a widely employed experimental practice whereby investigators pool tissues from multiple individuals (or organisms) and sequence the DNA (or RNA) without knowing which read originates from which individual (or organism) (8–11). In fact, for many species that cannot be individually cultured in laboratory conditions, pooled sequencing is the only means for obtaining sufficient experimental material as required by state-of-the-art sequencing technologies. When analyzed with an effective computational algorithm, this approach can accurately estimate the population frequency of transposition events.

When applied to pooled sequencing data, methods designed to detect structural variations in largely isogenic samples can only detect variations that are shared by most genomes in the pool. Discovering TE transpositions and estimating their frequencies using a pooled sequencing dataset present some unique computational challenges. Detecting rare TE transposition events with high confidence, identifying reads that are likely to support the same transposition event and overcoming biases stemming from the non-uniformity of sequencing depth across the genome are some of the difficulties involved. Kofler *et al.* designed an algorithm named PoPoolationTE to detect novel TE insertions and estimate their population frequency from pooled sequencing data. They applied PoPoolationTE to a natural population of *Drosophila* to study transposon evolution. In this article, we present an algorithm named TEMP that uses discordant mapping reads to detect TE polymorphisms relative to a reference genome, pinpoint the position of their junctions within genomic DNA and estimate their population frequencies from the pooled sequencing data. We demonstrated TEMP's performance by comparing it with PoPoolationTE, RetroSeq (an algorithm designed for detecting TE insertions in individual genomes), and two general-purpose structural variation discovery algorithms VariationHunter and GASVPro using simulated data. We further used TEMP to analyze several biological datasets in *Drosophila melanogaster* to demonstrate the unique biological insights that can be obtained using our algorithm. TEMP requires a curated library of transposon consensus sequences and cannot identify transposition events *de novo*. The TEMP software package is freely available at github: https://github.com/JialiUMassWengLab/TEMP.git or the TEMP webpage: http://zlab.umassmed.edu/TEMP/.

## MATERIALS AND METHODS

### Sequence mapping and the input files for TEMP

TEMP takes input files in the BAM format obtained by mapping sequencing reads to a reference genome. Throughout this article, we used BWA (v0.6.1-r104) (12) as the mapping software and *D. melanogaster* dm3 as the reference genome for mapping. Mapping was done using the BWA aln algorithm with command line options -n 3 -l 100 -R 10000, which allows for three mismatches. Other input files required by TEMP are transposon consensus sequences, which can be downloaded from Repbase (Version 17.07, http://www.girinst.org/repbase/), and RepeatMasker files containing the annotated TEs in the reference genome, which can be downloaded from the UCSC Genome Browser (http://genome.ucsc.edu/).

### The TEMP method for identifying TE insertions and absence

**Figure 1.**
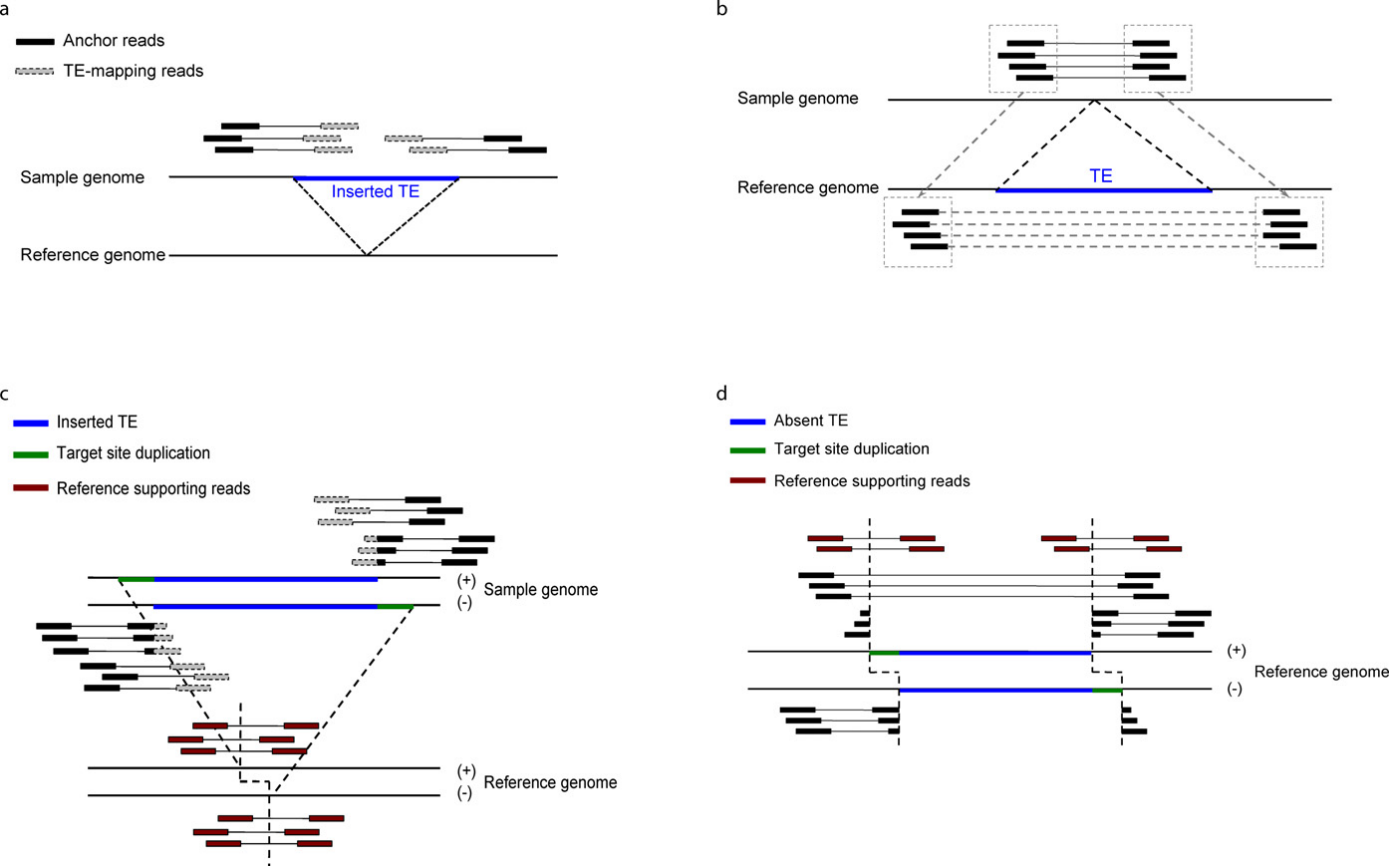
Diagrams depicting how TEMP detects presence (**a**) and absence (**b**) of insertion events and estimates junctions at base-pair resolution for presence (**c**) and absence (**d**) of insertion events.

In order to detect a TE insertion, TEMP first identifies all discordant read pairs (Figure 1), with one uniquely mapped read (the anchor read, or anchor) and a second read that is unmappable or maps to multiple distant locations. Those non-uniquely mapping reads are then compared to a library of consensus TE sequences. The TE to which the read maps with fewest mismatches determines the type of the TE insertion. For example, if the TE-mapping read maps to the *P-element* consensus sequence then it is likely that there is a *P-element* insertion in the vicinity of where the anchor maps. TEMP infers the orientation of the insertion by examining the genomic strand of the anchor and the transposon strand of the TE-mapping read. (Supplementary Figure S1). A single read pair is usually insufficient for inferring the precise junction. Therefore, TEMP first attempts to identify a genomic interval that includes the junction, called the interval estimate. This estimate is based on the average insert size of the sequencing library. The junction must be located in the interval beginning at the end of the anchor and extending into the genome by the length of the average insert size. The reads that support the same insertion event (i.e. the same type of TE, in the same genomic strand and with interval estimates that overlap by at least 1 nucleotide (nt)) are clustered and their intersecting region provides a refined interval estimate (Supplementary Figure S2).

To detect TE insertions that are present in the reference genome but absent in the sample genomes, TEMP first identifies all read pairs for which the distances between the two genome-mapping reads are significantly longer than average insert size (but less than 10k bps to avoid mapping artifacts) and then examines whether the intervening genomic region spans one or more known TEs as annotated in the reference genome. In order to prevent false positives, we require that both reads are uniquely mapped to the reference genome and that the distance between the two reads (after subtraction of the excised TEs) is consistent with the average insert size of the library. Read pairs that support the same event are clustered (Figure 1b). These structural alterations could reflect strain-specific excision of DNA elements, which move by a cut and paste mechanism, or could reflect polymorphic DNA or RNA element insertions that are specific to the reference genome. TEMP cannot distinguish between these alternatives unambiguously, but if the transposon is an RNA element transposing by the ‘copy and paste’ mechanism and its frequency is close to zero in most of the sample genomes, it is most likely that the element is a polymorphic insertion in the reference genome.

### Estimation of new junctions and transposition frequencies in a population

Based on the interval estimates obtained in the previous step, TEMP attempts to determine the new junctions created by transposition events up to base-pair resolution (base estimates) by taking advantage of reads that start in genomic sequence but are interrupted by transposon or non-contiguous genomic sequence (soft-clipped reads; Figure 1c and d).

For insertions, TEMP first extends the interval estimates obtained in the detection step by 20 bps in both directions. Soft-clipped reads that map within the extended interval are identified. For each such read, TEMP determines if the clipped portion of the read can be confidently explained by the insertion event (i.e. the clipped sequence corroborates the type and direction of the TE insertion determined by the previous step). We require the clipped portion to be at least 7 nt long and map perfectly to the appropriate TE sequence. When multiple junction estimates are identified, TEMP chooses the one supported by the most reads. We use a similar approach to estimate the junctions of TEs that are absent in the reference genome. Soft-clipped reads that map near the annotated boundaries of the absent TE are identified and the clipped portion of each read is examined to ensure that it maps to the sequence on the other side of the transposon. We note that base estimates of the junctions are strand-specific as the soft-clipped reads are mapped to only one strand of the genome. When the base estimates are not available, TEMP uses the midpoint of the interval estimates and the annotated TE boundaries as surrogates for insertion and absence, respectively.

For each detected presence or absence of transposon insertion, TEMP first compiles all the read pairs that support the transposition event, which include the discordant read pairs that define the transposition event and the soft-clipped reads that delineate its junctions with genomic sequence. TEMP also keeps track of another set of read pairs that originate from the genomes where the transposition event does not happen; these read pairs span the estimated junctions of the transposition (Figure 1). TEMP computes the ratio *T*/(*T*+*R*) as an estimate of the population frequency of the transposon, where *T* stands for the total number of read pairs that support the presence and *R* stands for the total number of read pairs that are consistent with the absence of the transposon insertion.

The workflow of TEMP is represented in Supplementary Figures S3 and S4.

### Simulation analysis

In each experiment, 50 insertions and 50 excisions were randomly placed across chromosome arm 2L of the *D. melanogaster* reference genome. For each simulated insertion, the TE family and insertion site coordinate were selected randomly. The entire sequence of the chosen TE was then inserted at the selected coordinate. For each simulated excision, an annotated transposon (as annotated in the output of the RepeatMasker program) was randomly selected and the entire sequence was deleted. The insertion and deletion operations were carried out using a genomic structural variation simulation package named *RSVSim* (v1.1.1) (13). Simulated read pairs with read length of 90 nucleotides (nt) following a normal distribution of insert sizes (500 ± 50 nt) were then generated from the simulated genome obtained in the previous step with four different sequencing depths (5X, 10X, 20X and 40X) using a profile-based Illumina paired-end reads simulator named *pIRS* (14). We used *pIRS* v1.1.0 with options -l 90 -m 500 -v 50 -e 0.0001 -a 0 -g 0, which simulated 90-nt long reads, with mean insert size set at 500 nt, standard deviation of insert sizes at 50 nt, sequencing error rate at 0.0001, no insertions or deletions in the reads, and no GC bias. To simulate various population frequencies of transposition events, we mixed reads generated from the simulated genome with reads generated from the reference genome at appropriate ratios. Finally, we mapped all the reads to dm3 using BWA and fed the mapping results to TEMP and other algorithms to evaluate their performance. The above procedure was repeated 100 times to obtain 5000 simulated insertions and 5000 simulated excisions.

To compare TEMP with other algorithms, we generated datasets by combining five independently simulated *Drosophila* chromosome 2L arms. Each simulated chromosome arm was generated as described above, and pair-end reads were simulated at 5X coverage. Each simulated dataset hence contained reads originating from the five simulated chromosome arms with an apparent coverage of 25X, and the process was repeated 20 times. We compared TEMP with PoPoolationTE (15), RetroSeq (16), VariationHunter (17) and GASVPro (18) on these datasets. The results are summarized in Table1. We evaluated PoPoolationTE (v1.02, https://code.google.com/p/popoolationte/), RetroSeq (https://github.com/tk2/RetroSeq), VariationHunter CommonLaw (v0.04, http://variationhunter.sourceforge.net/Home) and GASVPro-HQ (2013 Oct Release, http://code.google.com/p/gasv/). For PoPoolationTE we followed the typical workflow described at https://code.google.com/p/popoolationte/wiki/Workflow and used the parameters therein. For RetroSeq, we used the BAM format alignment file produced by the BWA aln algorithm as the input and chose the same parameters as described in the tutorial https://github.com/tk2/RetroSeq/wiki/RetroSeq-Tutorial. For VariationHunter-CL, we first mapped paired-end reads to the reference genome (dm3) using mrfast (v2.6.0.1, http://mrfast.sourceforge.net/) with parameters -min 400 –max 600 -e 3 (which allows for three mismatches and defines concordant insert sizes as between 400 nt and 600 nt), and then ran VH and multiInd_SetCover with default parameters. Structural variations supported by fewer than eight reads were discarded. For GASVPro-HQ, we used the BAM format alignment file produced by the BWA aln program as the ‘high quality unique mapping BAM file’ and default parameters. GASVPro produced a large number of predictions. We ranked the predictions by log-likelihood ratio and kept the top 250 predictions.

A simulated insertion was correctly recovered if the interval estimate given by TEMP included its true junction with the genomic DNA, and if the transposon family and direction of the insertion were determined correctly. A simulated excision was correctly recovered if TEMP reported the absence of the corresponding transposon. For a simulated transposition event, we considered its junction correctly identified if the base estimate TEMP reported lay within 5 nt of the true junction.

### Testing TEMP on pair-end sequencing data from the 1000 genomes project

BAM files containing alignments to the GRCh37 (hg19) human reference genome for four individuals (NA18517, NA19240, NA12156, NA12878) were downloaded from the data portal of the 1000 Genomes Project and merged to mimic a pooled sequencing dataset. We then ran TEMP on this dataset and predicted the presence and absence of TE insertions with frequency greater than or equal to 20% and covered by more than eight reads. TEMP predictions were compared with previously reported insertions and deletions involving these four individuals as deposited in structural variation database DGV (database of genomic variants, http://dgv.tcag.ca/dgv/app/home). Note that the structural variations in DGV include all types of changes in genomic DNA regardless whether they are caused by TEs.

### Hybrid dysgenesis population analysis

The small RNA sequencing and genomic deep sequencing datasets were downloaded from NCBI SRA database (SRP007937) and processed and analyzed as described in Khurana *et al.* (19). We define parental transposons as TE insertions with population frequencies greater than 10% in at least one of the parental strains (*w^1^* or Harwich). The frequency change of a parental transposon is defined by *FC* = *F*–(*H*+*W*)/2, where *F*, *W*, *H* represent frequency of the transposon in the *w^1^* x Har; *2–4 day* F1 population, the *w^1^* population and the Harwich population, respectively. The junction spanning small RNA reads need to be at least 21 nt long and map perfectly across the genome-transposon junction. We use the piRNA cluster annotation by Brennecke *et al.* (19), which includes 141 clusters in total (excluding the chrX_TAS cluster), occupying 4 924 944 bp of the dm3 genome. piRNA clusters are the genomic loci from which precursor piRNA transcripts are produced.

### The *Drosophila* Genetic Reference Panel datasets

We downloaded genomic deep sequencing data for 53 DGRP inbred lines (20) from NCBI SRA (Supplementary Table S1). Except for lines RAL-362, RAL-765 and RAL-517, the other 50 lines each had >20X sequencing coverage. We included those three lines with <20X coverage because they were the only lines with RNA-seq data. We mapped the reads to dm3 with the BWA aln algorithm, allowing for three mismatches and then ran TEMP on the BWA output files in the BAM format.

For TE insertion distribution analysis, 11 311 insertions that had frequencies greater than 80% in at least one of the inbred lines were chosen. We profiled the number of insertions in each of the five genomic features: promoters (2 kb upstream of an annotated transcription start site), exons (Flybase annotation), intron/UTR regions (regions within annotated genes but not in exons), intergenic regions (regions more than 2 kb from any annotated genes) and piRNA clusters for each TE family. A binomial test was performed to assess the statistical significance of enrichment or depletion for each TE family in each of the five genomic features and the Benjamini–Hochberg procedure (21) was used for multiple testing corrections. Only enrichments and depletions with *q*-values lower than 0.15 are shown in Supplementary Table S2. The annotation for genes and exons were obtained from FlyBase (Release 5.45) and the annotation for piRNA clusters was from Brennecke *et al.* (19) as described above.

For RNA-seq data, we downloaded seven datasets involving the three lines RAL-362, RAL-765 and RAL-517 and four progeny populations (Supplementary Table S1). The samples involving two lines were F1 samples (i.e. the progeny of the two indicated lines separated by ‘x’). We mapped the reads to the reference genome using Tophat (v2.0.8b with default parameters) and then used Cufflinks (v2.1.1 with default parameters) to compute the expression level for each gene (in Fragments Per Kilobase of transcript per Million, or FPKM). Thus for the three parental lines (RAL-362, RAL-765 and RAL-517), both genomic sequencing and RNA-seq data were available. To find the TE insertions that could potentially affect gene expression, we looked for genes that had (i) TE insertions with frequencies greater than 20% in their promoters, introns, exons or UTRs in only one of the three parental lines and (ii) expression levels in that line were more than 2-fold higher or lower (with a pseudo count of 0.5 FPKM) than the corresponding expression levels in the other two lines where the insertion was absent. The 48 genes obtained are listed in Supplementary Table S3 along with their expression levels in all seven lines.

### Sequence signature of TE insertions

We ran TEMP on all *Drosophila* genomic sequencing data we had and obtained 14 363 non-redundant insertion events with junctions on both strands detected. By calculating the difference between the coordinates of the junctions on two strands, we were able to estimate the length of target site duplications (TSDs) for each such insertion. We investigated the nucleotide composition of the sequence around the junctions by extending 15 bp both upstream and downstream from the midpoint between the junctions of the two strands for each insertion. We then ran MEME on these 30-bp long sequences to report up to five most significant motifs with lengths of 4–15 nt (MEM was ran with options -dna -mod zoops -nmotifs 5 -minw 4 -maxw 15 -pal, where dna and zoops indicate that there is zero or one motif site per input DNA sequence and pal indicates that we were looking for palindromic motifs.) This procedure was performed for each TE family to identify any sequence motifs that were enriched in the sequences surrounding the junctions. In the mono- and dinucleotide composition analysis, the same length (i.e. 30 bps) of flanking sequences (100 bps upstream and downstream of the junction) was selected as background, and the enrichment for each mono or dinucleotide was measured by the ratio of its frequency in the junction surrounding sequence over its frequency in flanking sequences.

## RESULTS

We first describe the general approach that TEMP takes for detecting TE polymorphisms and estimating their population frequencies. We then evaluate TEMP's performance on both simulated datasets and pooled human genome sequencing data. We compare TEMP with four other algorithms (PoPoolationTE, RetroSeq, VariationHunter and GASVPro) on the simulated data. To showcase how TEMP can be applied to studying biological problems, we use TEMP to investigate the inheritance patterns of polymorphic transpositions in *D. melanogaster* hybrid dysgenic strains. Finally, we analyze the genomic sequencing and RNA-seq data of 53 lines in a wild *D. melanogaster* population to learn about the molecular signatures of TE integration sites and potential molecular consequences of TE insertions.

### Overview of the TEMP method

TEMP detects the presence or absence of TE insertions in a population of sample genomes using read pairs that are mapped discordantly on a reference genome. Discordant read pairs with one read mapped uniquely to the reference genome and the other read mapped to TE sequence indicate sample-specific TE insertions (Figure 1a). Sample-specific absence of TEs can be detected by looking for read pairs that are separated by a distance substantially longer than the average insert size of the library and span a TE presents in the reference genome (Figure 1b). As detailed in Materials and Methods, TEMP can identify the presence and absence of TE insertions by identifying and sorting through discordant read pairs. TEMP then attempts to estimate the minimal genomic interval that includes the junction, called the interval estimate. For the insertions supported by sufficient numbers of reads, TEMP proceeds to refine the interval estimates to base-pair resolution by taking advantage of soft-clipped reads (Figure 1c and d).

In order to estimate the population frequency of transposition events, TEMP assumes that (i) the pool of sample genomes is a faithful representative of the population from which it is drawn and (ii) the number of read pairs supporting a transposition event is proportional to the frequency of the event in the pool of sample genomes. For each transposition event, TEMP keeps track of two sets of read pairs (including both discordant and soft-clipped read pairs), one set originating from the genomes where the transposition is present (*T* pairs) and the other set from the genomes where the transposition is absent (*R* pairs). TEMP computes the ratio *T*/(*T*+*R*) as an estimate of the population frequency of the transposon (see Materials and Methods for more details).

### Assessment of TEMP performance on simulated and biological datasets

As there are no pooled sequencing datasets for which the population frequencies of polymorphic transposition events are known, we first evaluated the performance of TEMP on a simulated dataset. We randomly inserted and deleted TE sequences in chromosome arm 2L of the *Drosophila* reference genome with the *RSVSim* (13) program and generated simulated reads from the simulated genomes using the *pIRS* (14) program. The simulated reads were mapped back to chr2L and TEMP was used to detect the presence and absence of insertions, resolve the junctions and estimate the population frequencies of the transposons.

**Figure 2.**
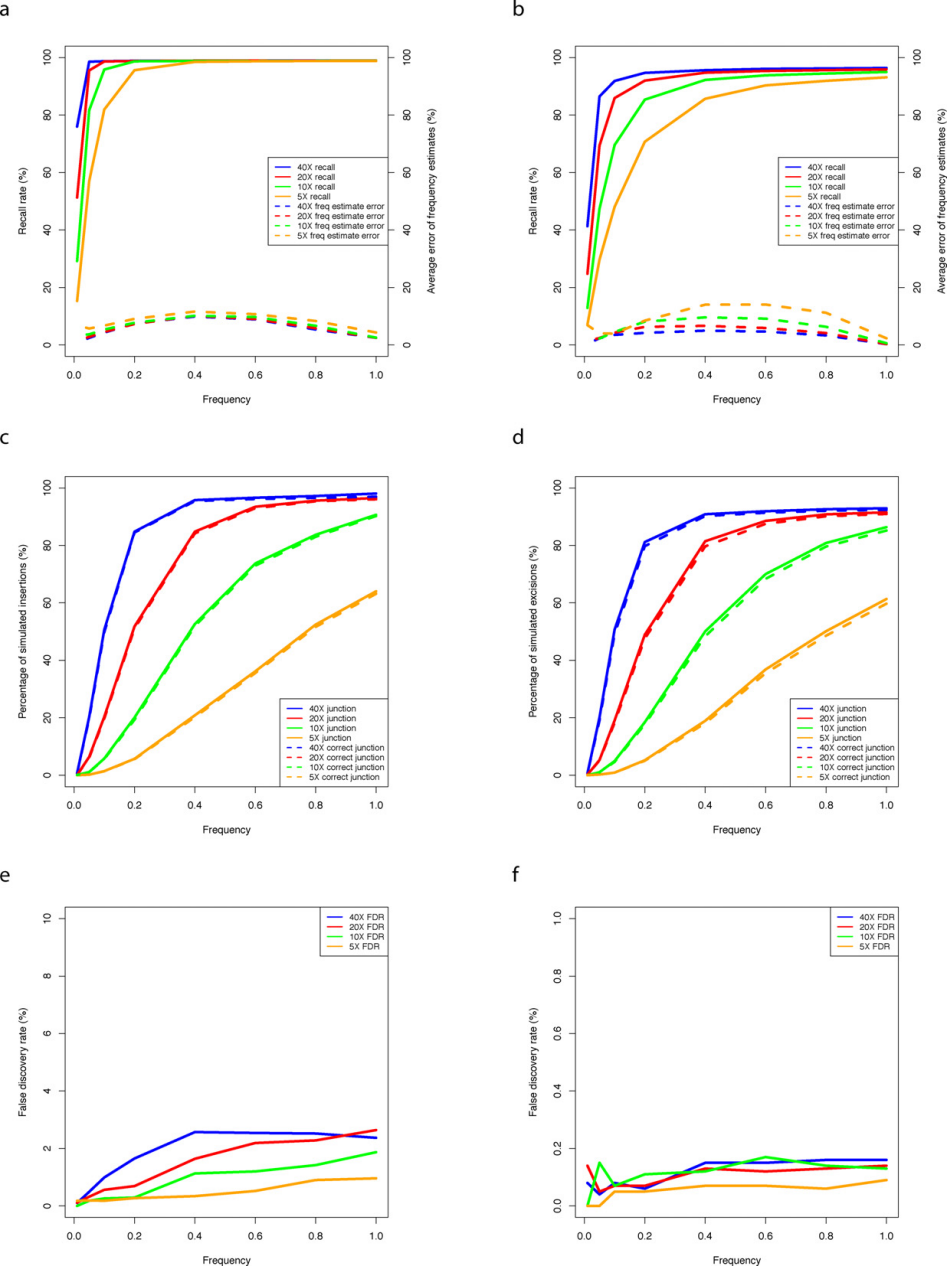
Evaluation of TEMP performance on a simulated dataset and the effects of sequencing depth and the population frequencies of the transposition events on the performance. The sequencing depth is color coded, with blue, red, green and orange denoting coverage 40X, 20X, 10X and 5X, respectively. Detection recall rates (solid lines) and average errors of frequency estimates (dashed lines) are plotted against population frequencies for presence (**a**) and absence (**b**) of insertion events. Percentage of transposition events for which TEMP identified junctions (solid lines) and for which TEMP *correctly* identified junctions (dashed lines) are plotted against population frequencies for presence (**c**) and absence (**d**) of insertion events. FDRs for detecting presence (**e**) and absence (**f**) of insertion events are plotted against population frequencies.

The performance of TEMP depends on the sequencing depth as well as the frequency of the transposition (Figure 2). TEMP performs better at higher sequencing depth, in terms of higher detection rates (Figure 2a and b, solid lines), more accurate estimates of population frequency (Figure 2a and b, dashed lines), higher probability of discovering the junctions (Figure 2c and d, solid lines) and more correctly recovered junctions (Figure 2c and d, dashed lines). Frequency of the target transpositions has similar effects. Those instances of the presence and absence of insertions with very low frequencies are often undetected and it is difficult to determine their precise junctions because there are only a few reads. False discovery rate (FDR) for TE insertion detection rises slowly with increasing sequencing depth and insertion frequency (Figure 2e). On the other hand, for TE absence detection the FDR remains low and flat across the range of sequencing depth and transposon frequency (Figure 2f).

At 20-fold genome coverage, which is easily achievable with current technology even for large mammalian genomes, TEMP is able to detect more than 95% of the presence and absence of insertions with population frequencies exceeding 20%. The average error of frequency estimation is <10% for presence and <9% for absence across the entire frequency range. Among the base estimates of the junctions reported by TEMP, more than 95% of them are correct. These results demonstrate that TEMP is effective in detecting sample specific TE insertion and absence, estimating their population frequency with high accuracy, and pinpointing the precise junctions for some of the transposition instances across a wide range of sequencing depths and transposition frequencies.

We compared TEMP with four other algorithms on a simulated dataset that mimics a pooled sequencing library: PoPoolationTE (15), an algorithm designed for detecting transposon insertions in pooled sequences; RetroSeq (16), designed for detecting transposon insertions in individual genomes; and two commonly used general-purpose structural variation discovery tools VariationHunter (17,22) and GASVPro (18). The results are presented in Table 1. TEMP achieved better performance in detecting both the presence and absence of TE insertion than the other four methods.

**Table 1.** Performance comparison between TEMP and transposon or structural variation discovery methods

Measures/Methods	TEMP	PoPoolationTE	RetroSeq	Variation Hunter-CL	GASVPro
TE insertion Sensitivity	***98.80%***	*88.50%*	*71.82%*	*0.00%*	*NA*
TE insertion Precision	***99.50%***	*92.45%*	*93.95%*	*0.00%*	*NA*
Average error of TE insertion frequency estimate	***7.27%***	*8.77%*	*NA*	*NA*	*NA*
TE absence Sensitivity	***93.09%***	*NA*	*NA*	*86.91%*	*72.37%*
TE absence Precision	***98.64%***	*NA*	*NA*	*79.74%*	*61.26%*
Average error of TE absence frequency estimate	***7.25%***	*NA*	*NA*	*NA*	*NA*

As a method designed for pooled sequencing data, PoPoolationTE performed worse than TEMP in terms of sensitivity (88.50% versus 98.80%), precision (92.45% versus 99.50%) and average error of estimating insertion frequency (8.77% versus 7.27%). RetroSeq is specifically designed for detecting TE insertions, but since it is not intended for handling pooled sequencing data, it does not estimate insertion frequency. RetroSeq achieved a low sensitivity (71.82%) and a high precision (93.95%). Neither PoPoolationTE nor RetroSeq is designed for detecting sample-specific absence of TEs in the reference genome. In comparison, TEMP can detect transposon absence with high sensitivity (93.09%), precision (98.64%) and low error in frequency estimate (7.25%). The two general-purpose structural variation detection algorithms VariationHunter and GASVPro could detect transposon absence, although with lower sensitivity and precision than TEMP (Table 1). Neither algorithm could detect TE insertion nor are they designed to estimate transposon frequency. GASVPro produced many false positives in detecting TE absence.

We also assessed TEMP's ability in detecting polymorphic TE transpositions in human genomes using whole-genome datasets generated by the 1000 Genomes Project (23). We pooled the reads from four individuals and ran TEMP to detect TE insertion and absence relative to the reference genome (GRCh37, see Supplementary Table S4 for details of TEMP predictions). Since there is little information on experimentally validated TE presence and absence genome-wide, we used structural variations deposited in the DGV for evaluating TEMP (24). Overall, 363 out of the 536 (67.7%) of insertions predicted by TEMP overlapped with insertions for these individuals in DGV and 423 out of the 1593 instances (26.5%) of absence predicted by TEMP overlapped with the deletions in DGV. The percentage of predictions that overlapped insertions and deletions in DGV went up to 81.5% and 95.5%, respectively, if we considered all individuals deposited in the DGV. Supplementary Table S4 also lists which DGV insertions or deletions that TEMP predictions matched. Thus TEMP works effectively in detecting transposition events in human genomes.

We evaluated the time complexity of TEMP on the same human whole-genome sequencing dataset. The combined dataset is equivalent to ∼12X coverage of the human genome and the insertion analysis took 1382 minutes on a Dell M605 node with 2 quad core AMD Opterons. The absence analysis took 721 minutes on the same machine.

### Identifying potentially selected TE insertions from pooled sequencing of hybrid-dysgenic population

We used TEMP to analyze the pooled genomic sequencing data of a wild-type strain of *D. melanogaster* (Harwich or Har in short), a lab strain (*w^1^*) and the offspring populations from crossing Har males with *w^1^* females. When Har females are mated with *w^1^* males, the first-generation offspring (F1) are normal; however, when Har males are mated with *w^1^* females, the offspring suffer from widespread TE transpositions, genomic instability and are initially sterile, a phenomenon known as hybrid dysgenesis (25–28). As the surviving female dysgenic flies age, they partially recover from the dysgenic phenotypes and begin to produce viable offspring, a change thought to be the result of *de novo* piRNA production in the ovaries (19).

We used TEMP to detect TE insertions relative to the reference genome and estimate their frequencies in each of the parental and progeny populations (Supplementary Table S5). This enables us to find insertions that show inheritance patterns potentially under adaptive selection. For a neutral insertion polymorphism, its population frequency in the progeny population should be close to the arithmetic mean of the frequencies in the two parental populations if the inheritance obeys Mendelian segregation. We therefore defined *frequency change* using a simple formula *FC* = *F*–(*H*+*W*)/2, where *F*, *H*, *W* denote the population frequency of a TE insertion in the dysgenic F1 population, the Harwich population and the *w^1^* population, respectively. A large positive value of frequency change suggests positive (adaptive) selection whereas a large negative value suggests negative (purifying) selection.

**Figure 3.**
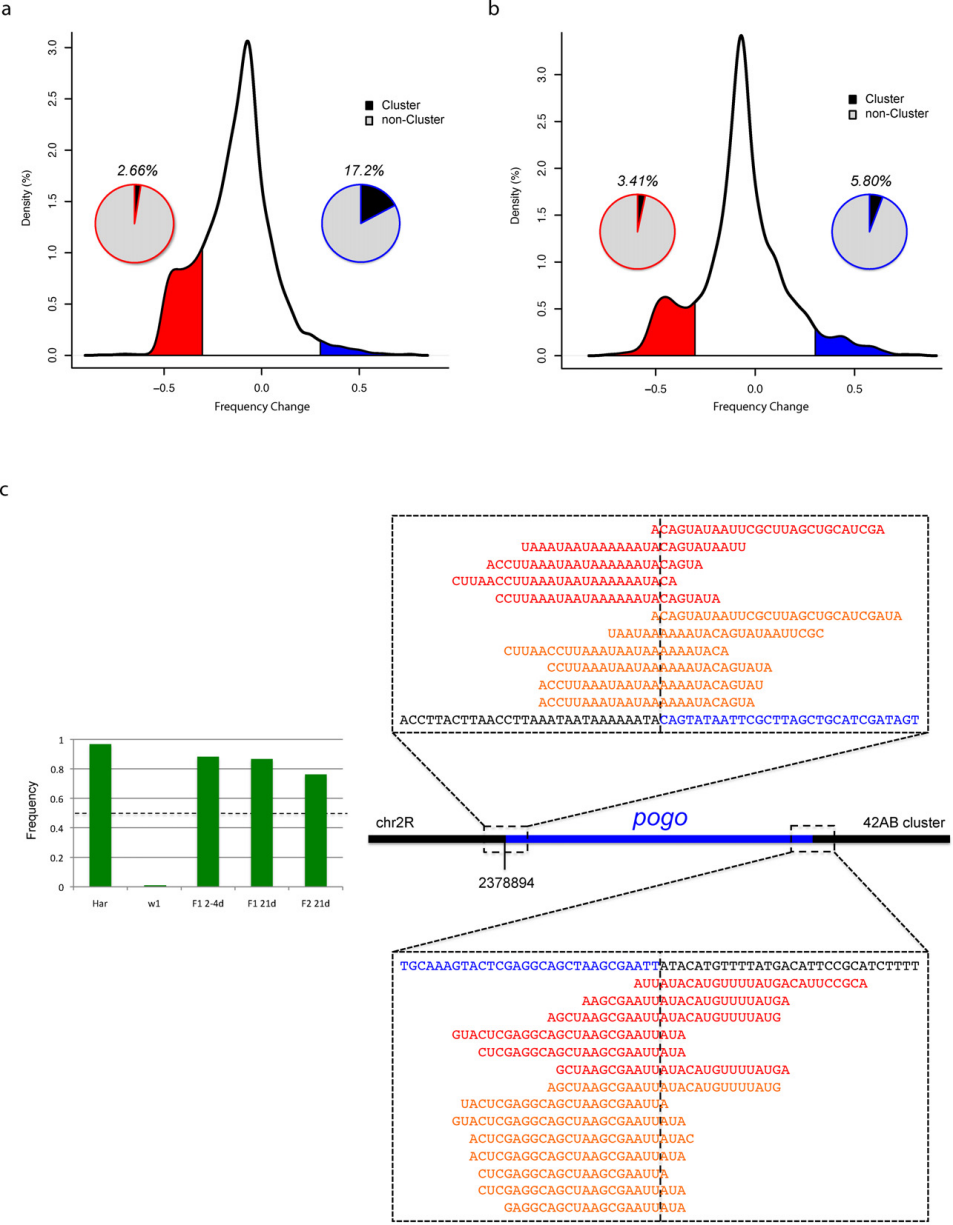
(**a**) Distribution of selection strength acted on parental TEs. Positively selected TEs (blue shaded region) shows enrichment for piRNA cluster residing TEs whereas negatively selected TEs (red shaded region) shows depletion for piRNA cluster residing TEs. The pie charts represent the percentages of piRNA cluster insertions (labeled) among the positively (or negatively) selected TEs. (**b**) Same as (a) except for F2 backcross progeny. (**c**) A *pogo* insertion within the 42AB piRNA cluster is under strong positive selection. It led to the *de novo* production of piRNAs as demonstrated by piRNA reads that span the insertion junctions in two F1 populations, *w^1^ X Har* 2–4 days (red) and *w^1^ X Har* 21 days (orange). The bar plots on the left show the frequency of the *pogo* insertion in the parental, F1 and F2 populations.

We computed the frequency change for each parental TE insertion (defined as the insertions whose frequencies exceed 10% in at least one of the parental populations) (Figure 3a). As expected, the vast majority of parental insertions have negative but close to zero frequency changes in the F1 population (Figure 3a), suggesting that they were under weak purifying selection. The most critical challenge facing the hybrid dysgenic flies was coping with hyperactive transpositions and any trait that helped suppress TE mobilization could be potentially selected for. Insertion of a TE into piRNA clusters can lead to production of piRNAs whose sequences are complementary to the TE, and these piRNAs can in turn silence the corresponding transposon genome-wide (29–31). Indeed, among insertions whose frequencies increased by 30% or more in the F1 population (*FC* ≥ 0.3), there were more insertions residing within piRNA clusters than expected (*P*-value = 5.38E-4, hypergeometric test). In contrast, among insertions with *FC* ≤ −0.3, there were fewer of them than expected in piRNA clusters (*P*-value = 8.02E-5, hypergeometric test) (Figure 3a). We also analyzed the germline DNA isolated from the ovaries of the second-generation progenies (F2) produced by backcrossing F1 dysgenic females to *w^1^* males. Again, we computed the *FC* for each parental TE insertion, i.e. those insertions whose frequency exceeded 10% in either F1 or *w^1^*. These F2 females did not suffer from hyperactive TE movement; accordingly, our data support that there were fewer insertions under negative selection than their parents (14.05% with *FC* ≤ −0.3 in F2 versus 19.09% in F1; *P*-value = 4.90E-5, *X*^2^-test). Moreover, there was no enrichment for TE insertions in piRNA clusters (*P*-value = 0.53, hypergeometric test), consistent with the notion that such insertions would not confer significant selective advantages in non-dysgenic individuals (Figure 3b). We note that according to the Wright–Fisher model with a population size of 100 (200 chromosomes), the probability of *FC* ≤ −0.3 or ≥ 0.3 or more extreme is smaller than 1E-15 (Supplementary Table S6). Therefore, the sites with *FC* ≤ −0.3 or ≥ 0.3 are likely under selective pressure. Moreover, at 20X sequencing depth TEMP's FDR is 1.17% for sites with frequency at 0.3 (Figure 2e). Therefore most of the sites with *FC* ≤ −0.3 or ≥ 0.3 represent actual change, not detection error.

We were able to resolve the junctions for one of the insertions that were both strongly selected for and lie within a piRNA cluster. A *pogo* insertion at position 2 378 892–2 378 894 of chromosome arm 2R (within the piRNA cluster 42AB) had a frequency of 96.77% in the Har population and was absent in the *w^1^* population. In the F1 hybrid dysgenic population the frequency of the same insertion is 88.24%, which far exceeded what would be expected from Mendelian inheritance—suggesting that it was under strong positive selection. Evidently, in addition to the F1 embryos that lacked a *pogo* insertion in both alleles, some of the F1 embryos that were heterozygous for this *pogo* insertion did not mature to adulthood. As shown by the large number of piRNA reads that mapped across the unique junctions produced by the insertion, this insertion led to *de novo* production of piRNAs and probably helped repress transposition of the *pogo* element, giving the individuals a selective advantage (Figure 3c). Interestingly, the same insertion also exhibited higher than expected frequency in the F2 backcross progeny, suggesting a persisted adaptive selection at this locus even though the backcross did not induce hybrid dysgenesis (Figure 3c, bar plots).

### Sequence signatures and potential effects on gene expression of TE insertions

The *D. melanogaster* Genetic Reference Panel (DGRP) is a community resource of inbred lines of fruit flies derived from a wild population (20). In freeze 1.0, the genomes of 168 inbred lines have been sequenced and the sequencing data are publicly available. Moreover, the RNA-seq data for three of these lines are also available, as well as the RNA-seq data on four progeny populations of these three lines. We selected 53 lines with the highest genome sequencing coverage and applied TEMP to detect the presence and absence of TE insertions. TEMP detected in total 11 316 instances of the presence and 1378 instances of the absence of transposons that had frequency greater than 80% in at least one line (Supplementary Tables S7). The distribution of TE insertions across the genome showed that most TE insertions are enriched in intronic and intergenic regions and depleted in exonic regions (Supplementary Table S2). This is consistent with a recent report on a related dataset (32).

We also used TEMP to pinpoint the junctions in both the DGRP datasets and the hybrid dysgenic datasets. TEMP reported the positions for 14 363 non-redundant junctions at base pair resolution, which enabled us to investigate the sequence signatures near the TE insertion sites including the length of TSDs, the dinucleotide composition of target site sequences and potential sequence motifs at target sites that may reveal the sequence preferences of the integrases (Supplementary Figure S5).

**Figure 4.**
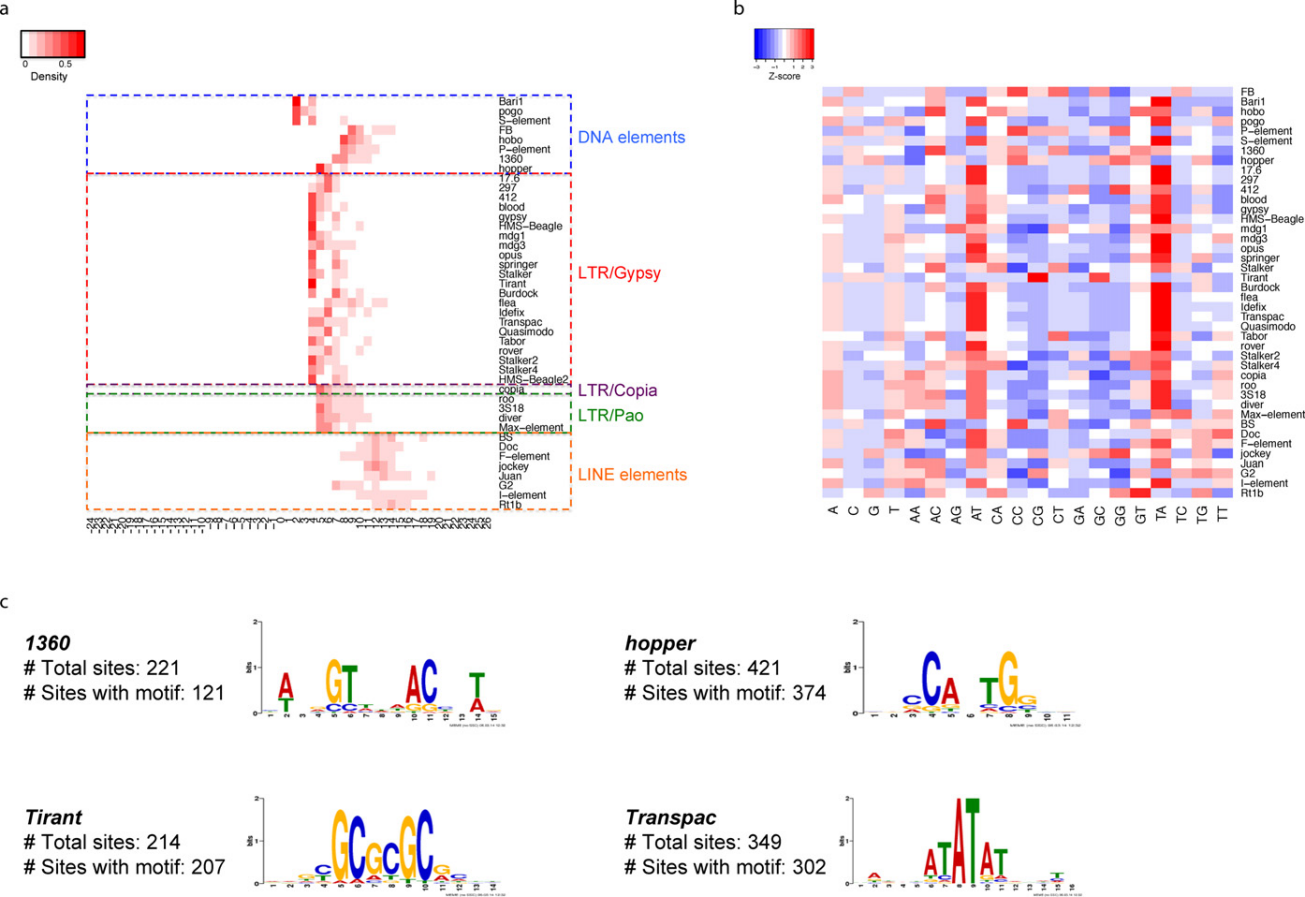
(**a**) Length distribution of TSDs (depletions) for TEs. The TEs are grouped according to families. Negative values on the x-axis denote the length of target site depletions. (**b**) Dinucleotide composition around target sites. Each row is normalized and the Z-score for each entry is color coded with red represents enrichment and blue represents depletion. (**c**) Sequence motifs for TE elements *1360*, *Tirant*, *Transpac* and *hopper*.

There were 44 TE families for which we detected more than 50 non-redundant target sites. Most of these TE families exhibited narrow TSD length distributions (Supplementary Figure S6). Strikingly, TEs in the same super-family showed very similar TSD length distributions (Figure 4a) except for DNA elements. TEs in most LTR/Gypsy super-families showed 4-nt-long TSDs and nearly all TEs in the LTR/Copia and LTR/Pao super-family produced 5-nt-long TSDs. This interesting pattern probably reflects the evolutionary relationship among TEs, as integrases encoded by the TEs within the same super-family are more likely to share similar sequences and functional features (33,34). LINE elements had much longer TSDs and much broader TSD distributions compared with other retro-transposon super-families (Figure 4a), which is consistent with previous findings about the L1 element in the human genome (35,36).

We also discovered that the genomic sequences around predicted insertion junctions (±15 nt) of many TEs are AT rich, with the AT and TA dinucleotides being most prevalent (Figure 4b). The enriched sequence motif around the junctions is a simple dinucleotide repeat for many TEs (Supplementary Table S8), which is consistent with the mono- and dinucleotide composition analysis. As exceptions, we detected high-information-content motifs around the insertion sites of the *hopper*, *1360*, *Tirant* and *Transpac* elements, suggesting that their integrases or transposases may possess sequence specificity (Figure 4c).

**Figure 5.**
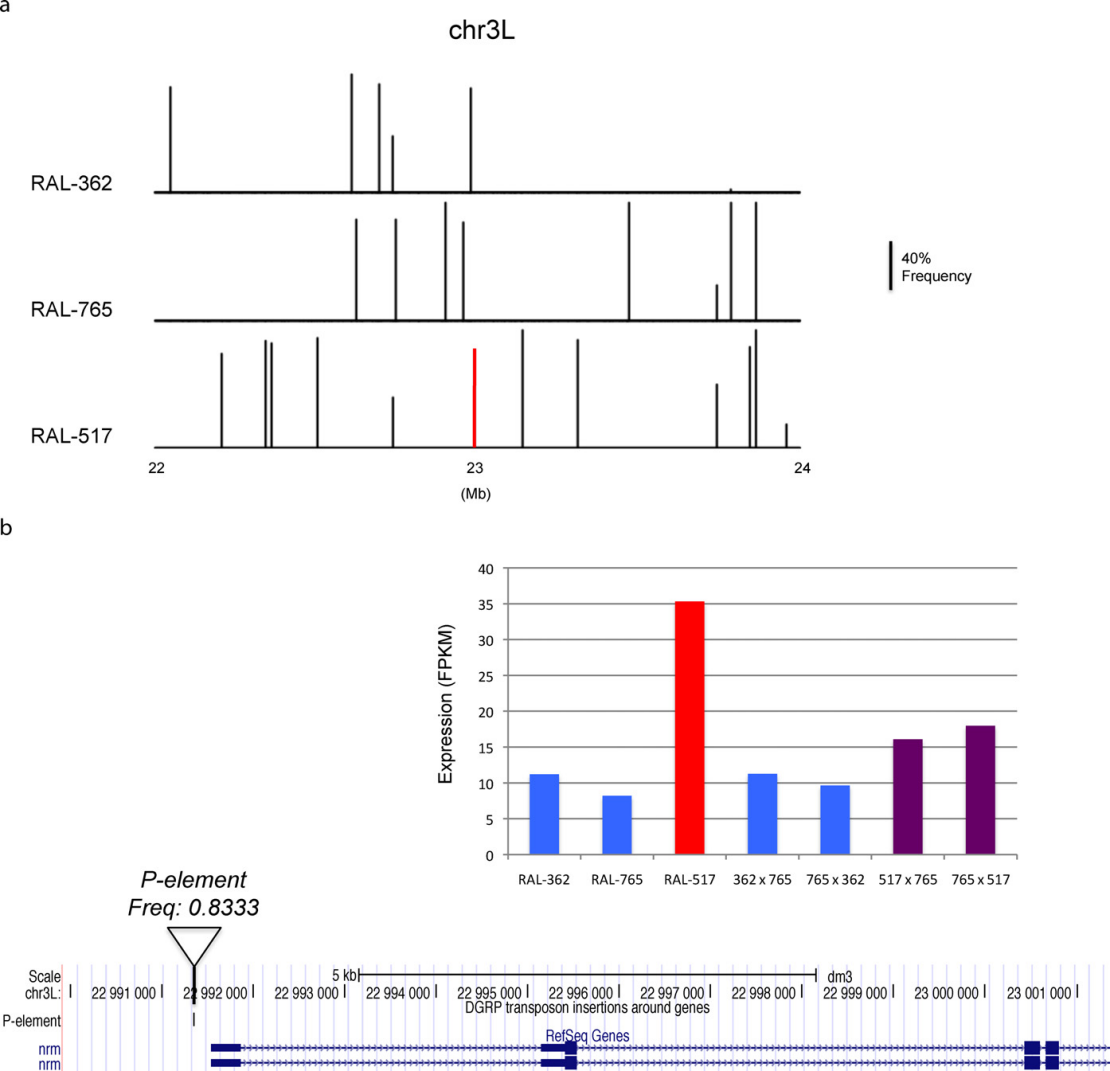
(**a**) Distribution of unique TE insertions of three DGRP strains in a region of chromosome arm 3L. The heights of the bars are proportional to the estimated population frequencies. The red bar in strain RAL-517 near 23Mb is a *P-element* insertion at the promoter of the *nrm* gene and its detailed view is presented in (b). (**b**) A *P-element* insertion at the promoter regions of the *nrm* gene in strain RAL-517 is correlated with a 3.65-fold increase in its expression level. The bar plot shows the expression level of *nrm* in the three lines as well as the four F1 progeny samples. The expression of *nrm* is higher for the progeny populations of RAL-517 (purple bars) than the progeny produced by crossing the other two strains.

TE transposition is one of the main sources of genomic variability. Relating transposition polymorphisms to variations at phenotypic and molecular levels is crucial for understanding how transposition shapes the genomic landscape and contributes to evolution (37–39). By integrating RNA-seq data for three DGRP lines with their respective genomic sequencing data, we searched for TE insertions that are likely to affect gene expression. More specifically, we looked for insertions that were in the promoter region or the gene body for which expression level of the affected gene changed by more than 2-fold. We identified 48 insertions that were associated with changes in gene expression. For example, *nrm* encodes a protein important for synaptic target recognition, and a *P-element* insertion at the promoter of *nrm* that is unique to strain RAL-517 is associated with a more than 3-fold increase in *nrm* expression (Figure 5a and b). Crosses with a strain showing lower expression produced progeny with intermediate expression levels, strongly suggesting that increased expression is inherited in the F1 generation (Figure 5b). The correlation between TE insertion and altered gene expression suggests a causal relationship, although other background variants can also contribute to the change in expression. Using TEMP to detect TE insertions and estimate their frequencies genome-wide, users will be able to correlate transposition polymorphisms with phenotypes and biological processes and identify candidate sites for experimental validation.

## DISCUSSION

Transposition of TEs is a widespread phenomenon that destabilizes the genome, but may also produce beneficial genetic diversity. The rapid development of high-throughput sequencing techniques offers unprecedented opportunities for detecting TE transpositions in a variety of samples. We described TEMP, an algorithm that can detect TE insertion and absence, pinpoint their junctions with genomic DNA at base pair resolution and estimate their frequencies in the population. Our analysis on both simulated and biological datasets demonstrates that TEMP is a reliable and useful tool for studying TE transpositions at both population and molecular levels and can be applied to a wide variety of datasets to accomplish quantitative analysis and generate testable hypotheses.

One limitation of TEMP is that it requires a curated library of transposon consensus sequences, namely the RepBase (40), and cannot identify transposition events *de novo*. Thus for a newly sequenced genome, one first needs to apply *de novo* repeat identification algorithms such as RECON (41) or RepeatScout (42) to build a library of transposon consensus sequences, before one can use TEMP to identify the presence and absence of these transposons in populations of the same species. One idea that may aid such an analysis is to perform *de novo* assembly of the reads of the test population that do not map to the reference genome. This may yield longer sequences which, once aligned back to the reference genome, can reveal insertion or deletion junctions. Instead of transposon consensus sequences, the PoPoolationTE algorithm uses a database of many diverged sequences for each TE family. PoPoolationTE therefore may have a higher sensitivity than TEMP with detecting highly diverged TE copies.

The ability of TEMP to detect insertions genome wide and identify junctions at base pair resolution for thousands of sites enabled us to better understand the molecular mechanisms of TE integration. Linheiro and Bergman examined TSD lengths and target site motifs on 166 DGRP datasets (34). Among the 25 TEs whose TSD lengths were reported in both studies, 19 of them had exactly the same TSD length (Supplementary Table S9). Linheiro and Bergman treated paired-end sequencing data as independent reads and used only junction-spanning reads to identify TE insertion target sites, possibly restricting the sensitivity of their method. Indeed, for each of the six TEs that the two studies disagreed, Linheiro and Bergman identified fewer than 20 non-redundant insertion sites, while we identified 50 or more sites. We also compared the target site motifs identified in the two studies and they mostly agree.

Transposition is proposed to produce both beneficial and deleterious changes in genome organization. To determine the utility of TEMP in defining the molecular consequences of transposon insertion, we applied TEMP to analyze dysgenic hybrids as well as 53 strains derived from independent wild populations of *Drosophila* and identified over 14 000 high frequency insertions at base pair resolution. Analysis of RNA-seq data from three of these strains, and the F1 progeny of inter-strain crosses, showed that many of these insertions were linked to heritable changes in gene expression (Figure 5b). These findings raise the possibility that strain-specific transposon insertions that modify gene expression can sweep through populations, perhaps because they provide a reproductive benefit. This can be directly tested by crossing strains and following the inheritance patterns of specific insertions using TEMP and measuring gene expression using RNA sequencing.

Our analysis of hybrid dysgenesis shows that transposition can also alter expression of small non-coding RNAs. Transposons are silenced by piRNAs that are deposited in the oocyte. In the early embryo, the piRNA pool is therefore derived exclusively from the maternal genome. Hybrid dysgenesis is triggered during crosses in which the sperm carries a transposon that is not represented in the maternal genome. Transposon activation in the hybrid germline leads to adult female sterility. We previously showed that paternal introduction of *P-element* transposons activated both the invading *P-element* and resident transposons that were shared by the maternal and paternal genomes. Remarkably, the dysgenic F1 females regained fertility with age, as they silenced *P-element* and resident elements. Furthermore, we demonstrated that this was linked to accumulation of *new* transposon insertions (i.e. not in either of the parental genomes) in the heterochromatic clusters that produced piRNAs, and that these insertions were the source of novel piRNAs that appeared to enhance silencing. Here, we used TEMP to estimate the frequencies of *existing* TE insertions in parental and progeny populations, and found that TE insertions within piRNA clusters were under positive selection in F1 dysgenic females. This finding, along with our earlier study, indicates that both *de novo* and inherited transposon insertions into piRNA clusters are under positive selection in dysgenic hybrids, where they appear to enhance silencing by promoting piRNA production.

Our studies thus show that transposition can alter both coding and non-coding RNA expression, and suggest that these modifications can generate beneficial genetic variation. The paradigm of sequencing parental and progeny populations, estimating the population frequencies of the transposition polymorphisms with TEMP and then identifying potentially selected polymorphisms can be applied to a wide range of systems to study the inheritance of transposition polymorphisms and their biological consequences.

## AVAILABILITY

Our software and the simulation datasets are available at TEMP website: http://zlab.umassmed.edu/TEMP/

## SUPPLEMENTARY DATA

Supplementary Data are available at NAR Online.

SUPPLEMENTARY DATA
