# Supplementary material for: TEMP: a computational method for analyzing transposable element polymorphism in populations
Source: Nucleic Acids Res. 2014 Apr 21;42(11):6826–38. doi: 10.1093/nar/gku323 (PMC4066757; doi:10.1093/nar/gku323)
Supplement: SUPPLEMENTARY DATA [file supp_42_11_6826__index.html]

TEMP: a computational method for analyzing transposable element polymorphism in populations — SUPPLEMENTARY DATA 

# TEMP: a computational method for analyzing transposable element polymorphism in populations

## SUPPLEMENTARY DATA

**Files in this Data Supplement:**

- SUPPLEMENTARY DATA
