# Supplementary material for: TEMP: a computational method for analyzing transposable element polymorphism in populations
Source: Nucleic Acids Res. 2014 Apr 21;42(11):6826–38. doi: 10.1093/nar/gku323 (PMC4066757; doi:10.1093/nar/gku323)
Supplement: SUPPLEMENTARY DATA [file supp_gku323_nar-03732-z-2013-File008.zip › NAR_supplementary_material/Supplementary Material.pdf]

Supplementary Material

Figure S1
A schematic representation of how TEMP determines the direction of a TE insertion.

Figure S2
A schematic representation of how TEMP determines the interval estimates of a TE insertion.

Figure S3
Workflow of how TEMP analyzes polymorphic TE insertions.

Figure S4
Workflow of how TEMP analyzes polymorphic absence of TEs.

Figure S5
Schematic depictions of the way we define sequences around break sites and the flanking sequences.

Figure S6
Histograms of TSD length distribution for 44 TE elements.

Table S1
DGRP genomic DNA sequencing and mRNA sequencing datasets used in our analysis.

Table S2
Enrichment and depletion of TE insertions in various genomic features (the DGRP dataset).

Table S3
Genes whose expression level is affected by TE insertions in their promoter region or gene body (the DGRP dataset).

Table S4
TEMP predicted TE insertion and absence in four human individuals (data from the 1000 genome project).

Table S5
TE insertion sites detected by TEMP in the 53 inbred lines from DGRP.

Table S6
Instances of TE absence detected by TEMP in the 53 inbred lines from DGRP.

Table S7
Sequence motifs enriched in sequences around TE insertion break sites.

Figure S1

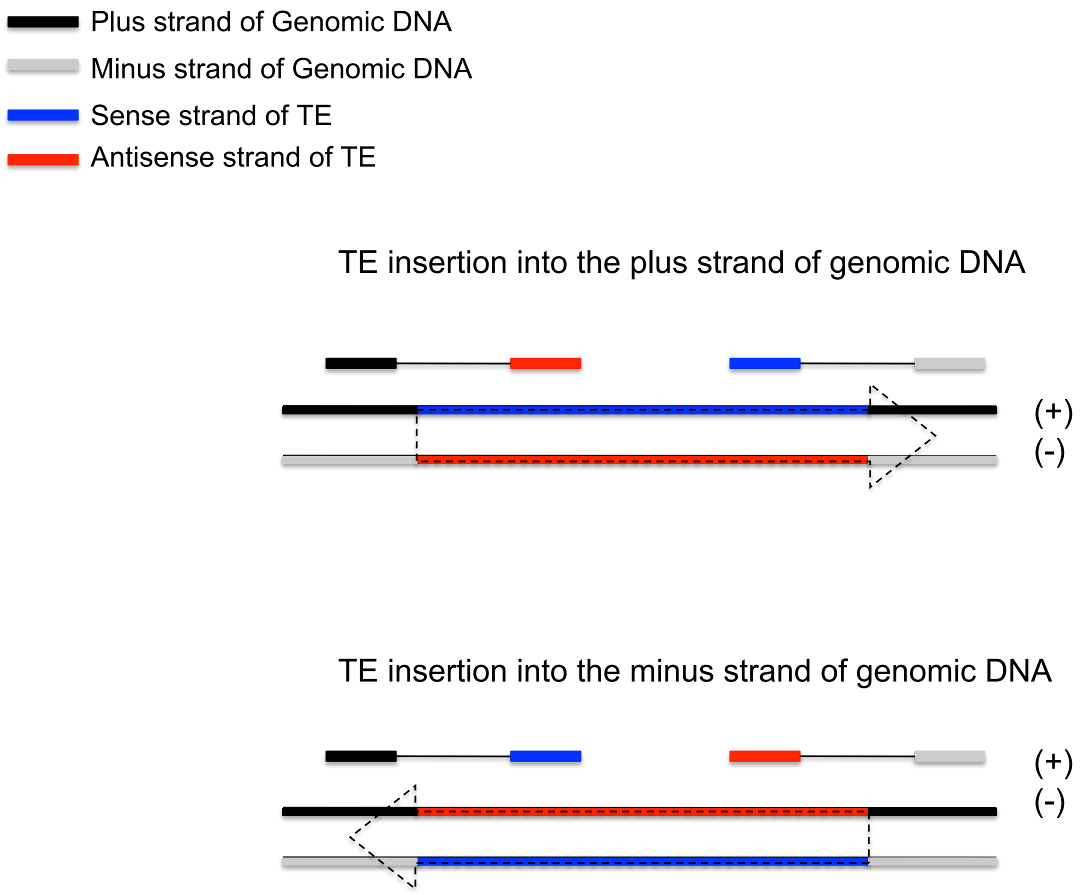

Determine the direction of the inserted TE. For a particular TE, the sequence supplied in the TE sequence database is the sense strand sequence. By definition, the two reads of a pair must come from two opposite strands. Therefore if the anchor read maps to the plus strand of the genome and the TE-mapping read maps to the antisense strand of the TE, the TE is inserted into the plus strand of the genomic DNA (upper panel). The same is true when the anchor read maps to the minus strand of the genome while TE-mapping read maps to the sense strand of the TE. On the contrary, if the anchor read maps to the plus strand of the genome and the TE-mapping read maps to the sense strand or the anchor read maps to the minus strand of the genome and the TE-mapping read maps to the antisense strand of the TE, then the plus strand of the TE is inserted into the minus strand of the genomic DNA (lower panel).

Figure S2

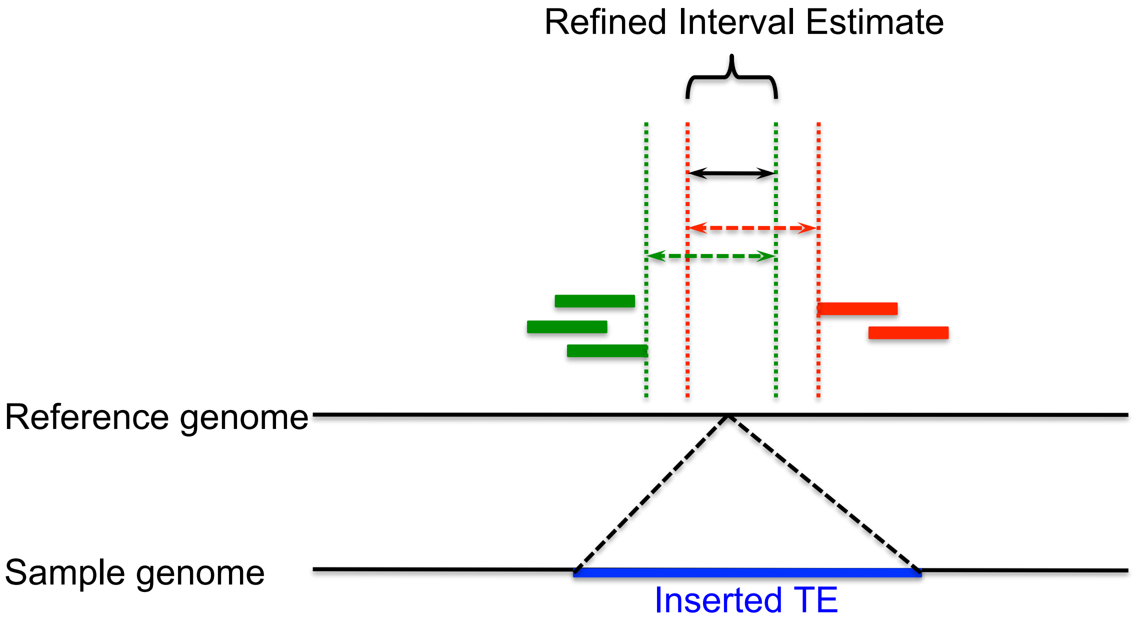

Compute the interval estimates for TE insertions. First anchor fragments that overlap by at least one nucleotide and support the same insertion are clustered (e.g., the green cluster and the red cluster in the figure, which map to the plus and minus strands of the genome respectively). For each cluster, TEMP defines an interval that starts at the 3′-end of the cluster if the cluster consists of fragments mapping to the plus strand of the genome and at the 5′-end of the cluster if the cluster contains fragments mapping to the minus strand of the genome. The length of the interval is $l - 2 \times r$, where l is the average insert size of the library and r is the read length. If there exists more than one cluster, TEMP takes the intersection of the intervals of the clusters as a more refined interval estimate.

Figure S3

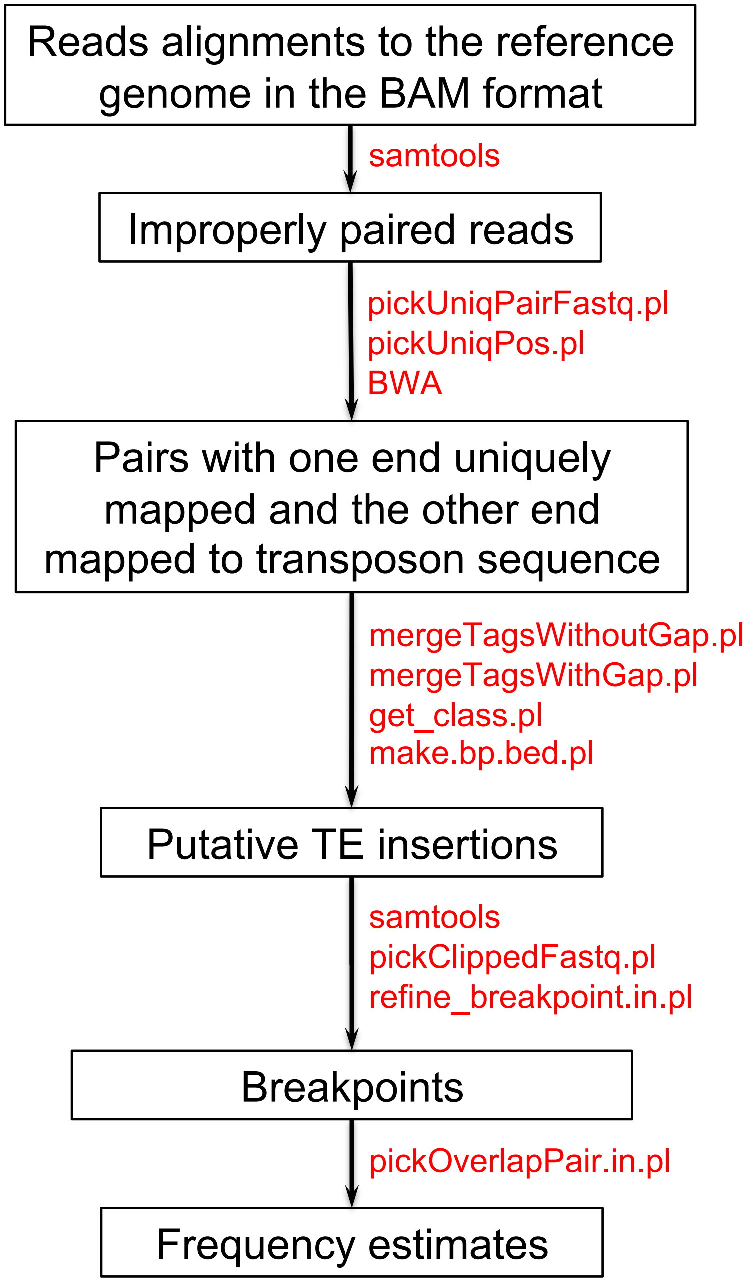

Workflow of how TEMP analyzes polymorphic TE insertions. The software and scripts required by each step are highlighted in red.

Figure S4

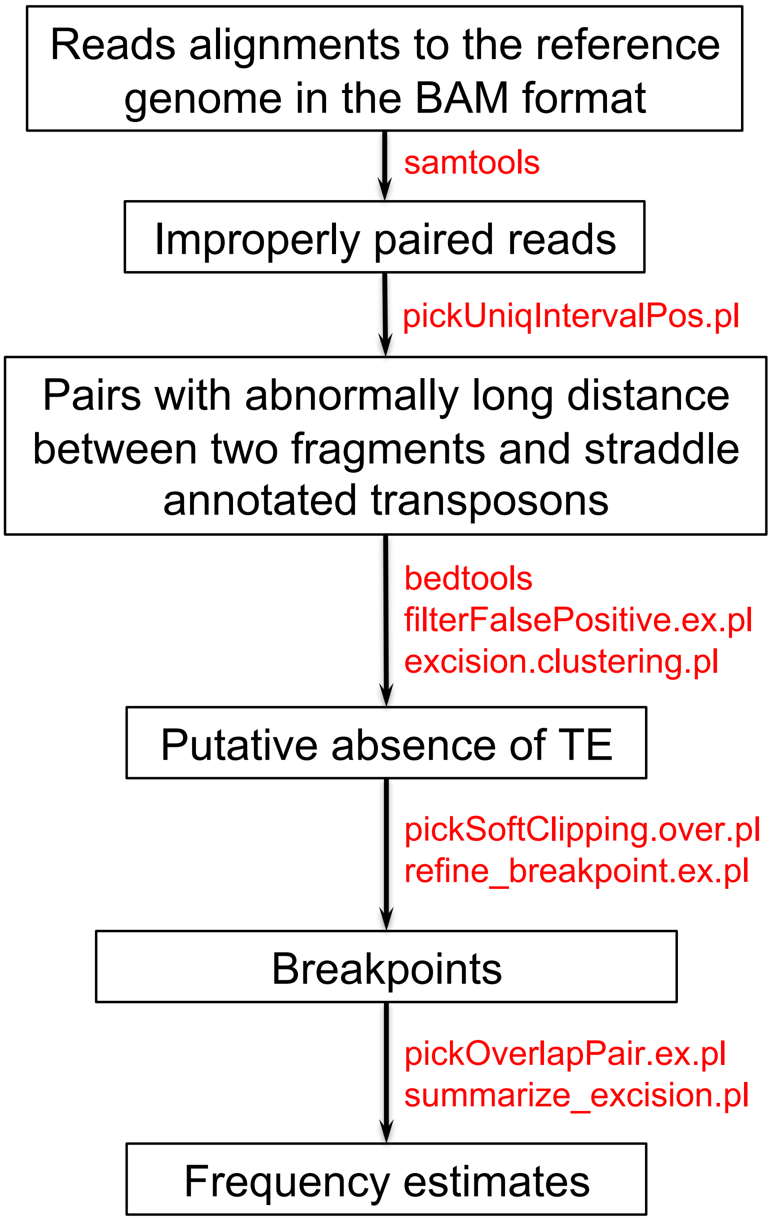

Workflow of how TEMP analyzes polymorphic absence of TEs. The software and scripts required by each step are highlighted in red.

Figure S5

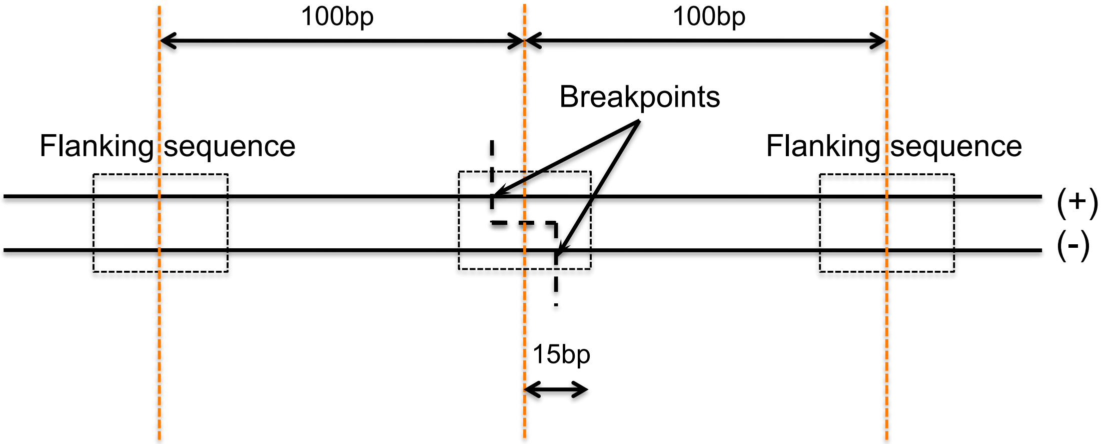

For dinucleotide composition and sequence motif analysis, we define the sequence around breakpoints as follows. We take the midpoint of the breakpoints on the two strands as identified by TEMP and extend in both directions by 15bp. In order to obtain a matching background, we take two flanking sequences of the same length 100bp upstream and downstream.

Figure S6

Histograms of target site duplication length distribution. There are in total 44 pages depicting 44 of the TEs for which TEMP identified more than 50 break sites each. The x-axis represents the length of TSD and the y-axis denotes the frequency of the corresponding TSD length.

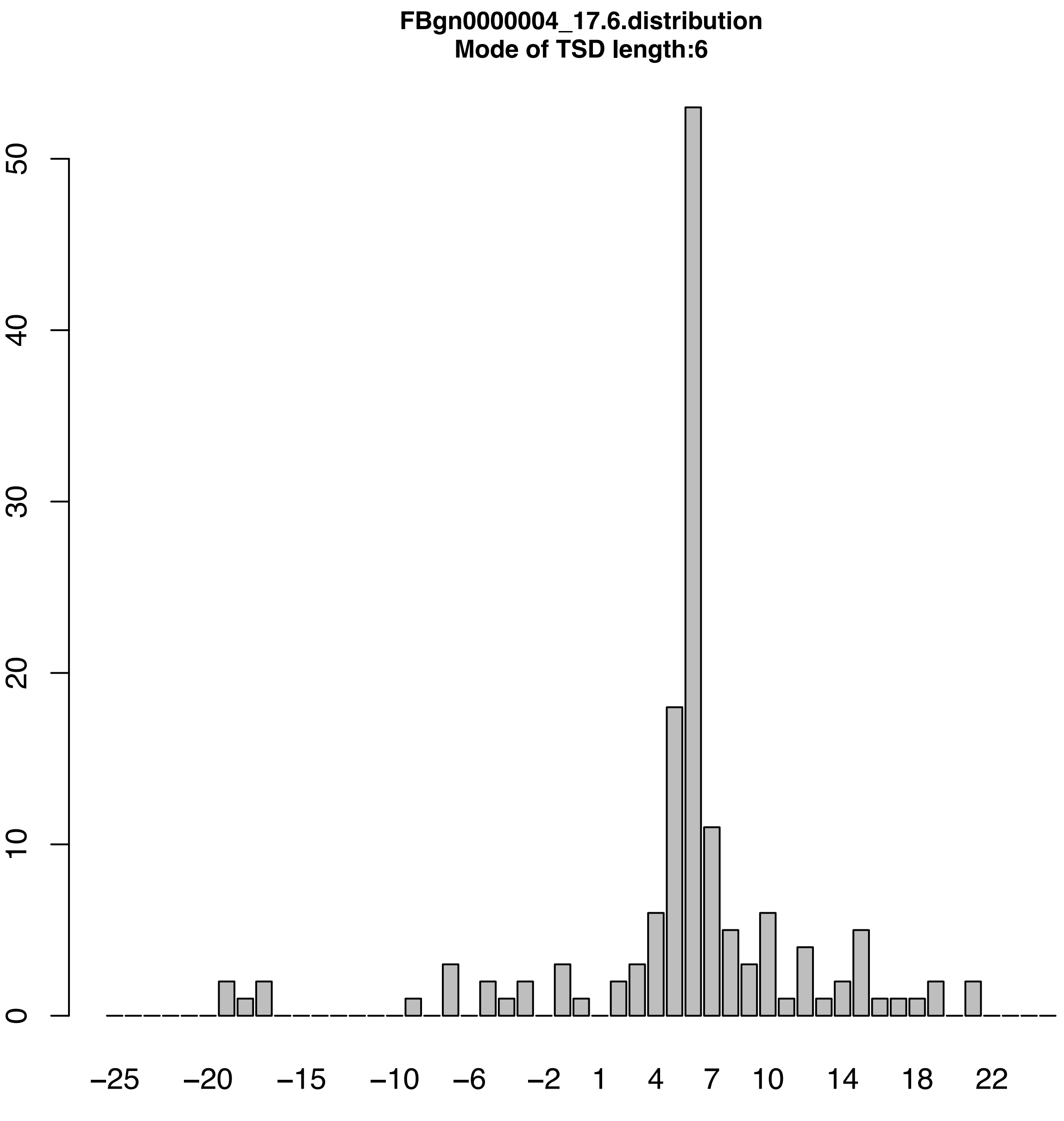
FBgn0000004_17.6.distribution
Mode of TSD length:6
0
10
20
30
40
50
−25
−20
−15
−10
−6
−2
1
4
7
10
14
18
22

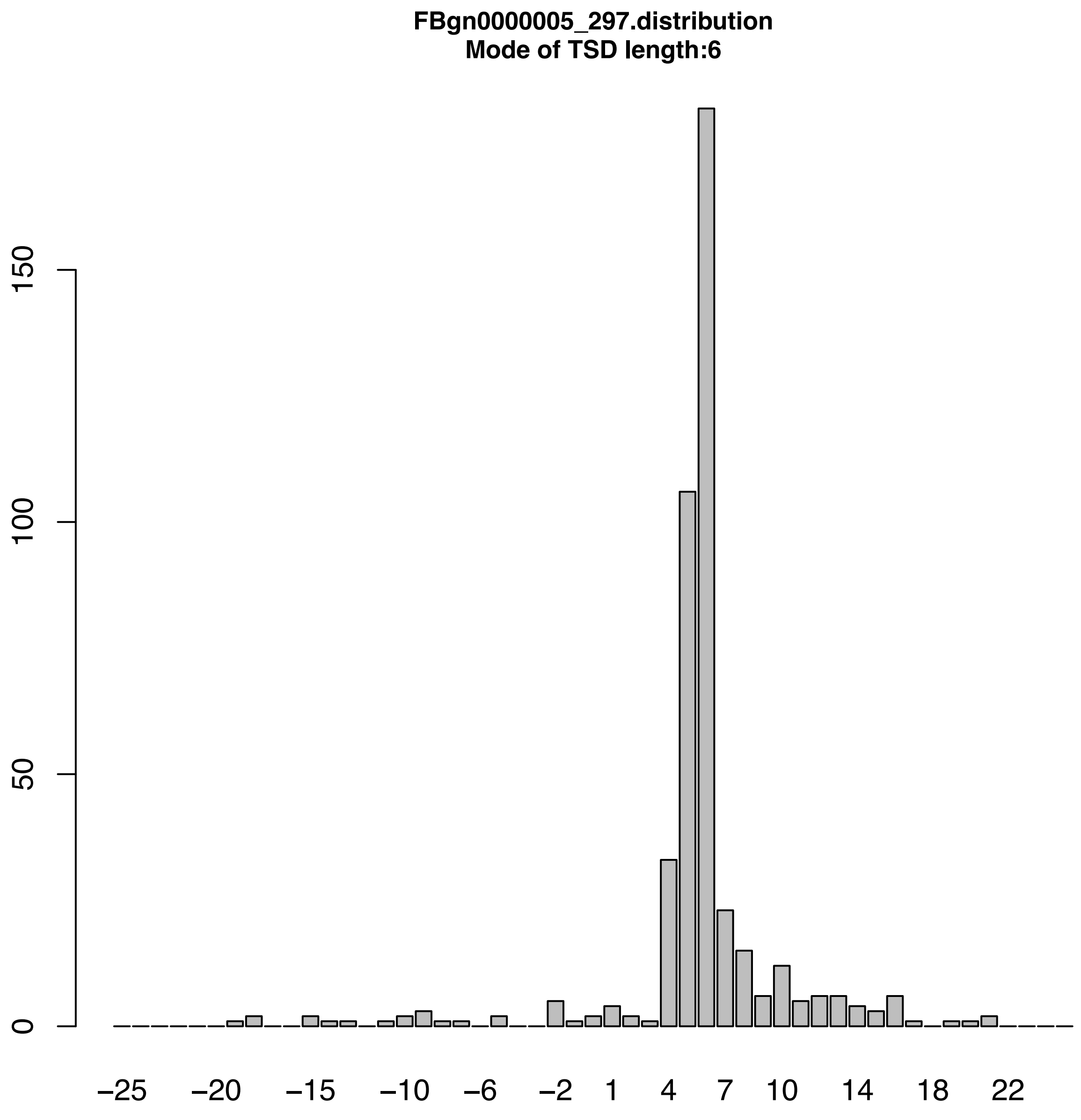
FBgn0000005_297.distribution
Mode of TSD length:6
0
50
100
150
−25
−20
−15
−10
−6
−2
1
4
7
10
14
18
22

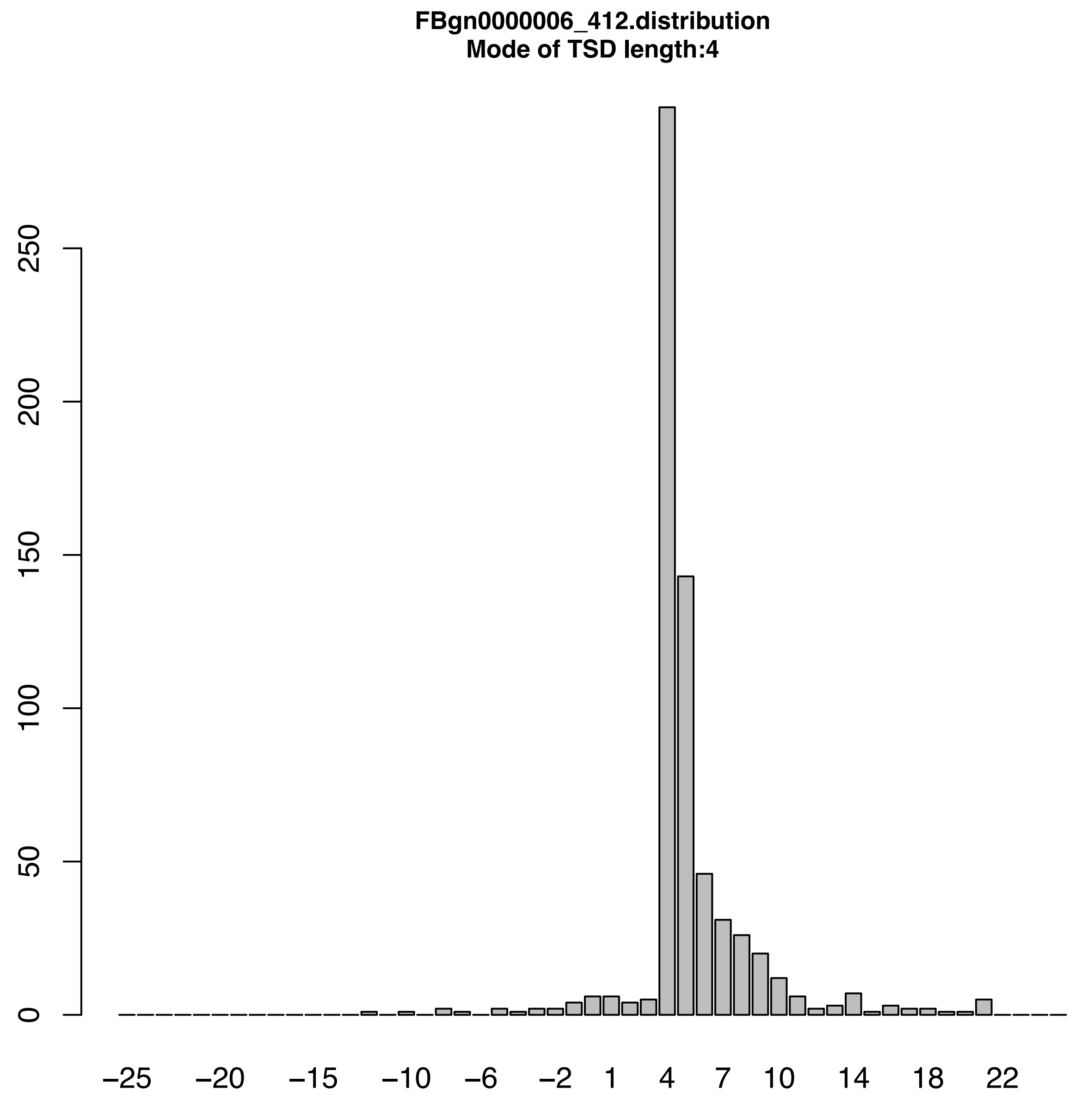
FBgn0000006_412.distribution
Mode of TSD length:4
0
50
100
150
200
250
−25
−20
−15
−10
−6
−2
1
4
7
10
14
18
22

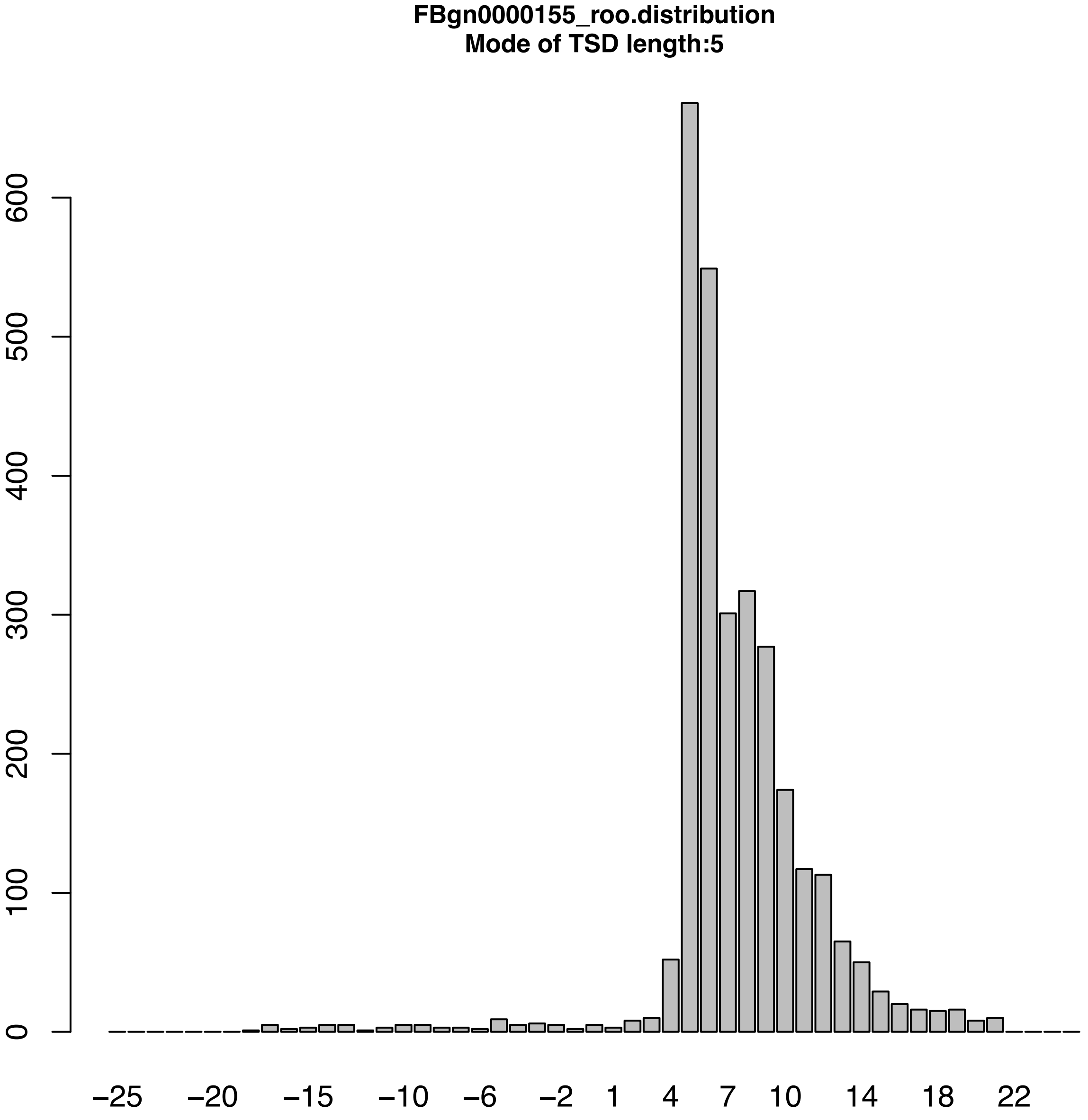
FBgn0000155_roo.distribution
Mode of TSD length:5
0
100
200
300
400
500
600
−25
−20
−15
−10
−6
−2
1
4
7
10
14
18
22

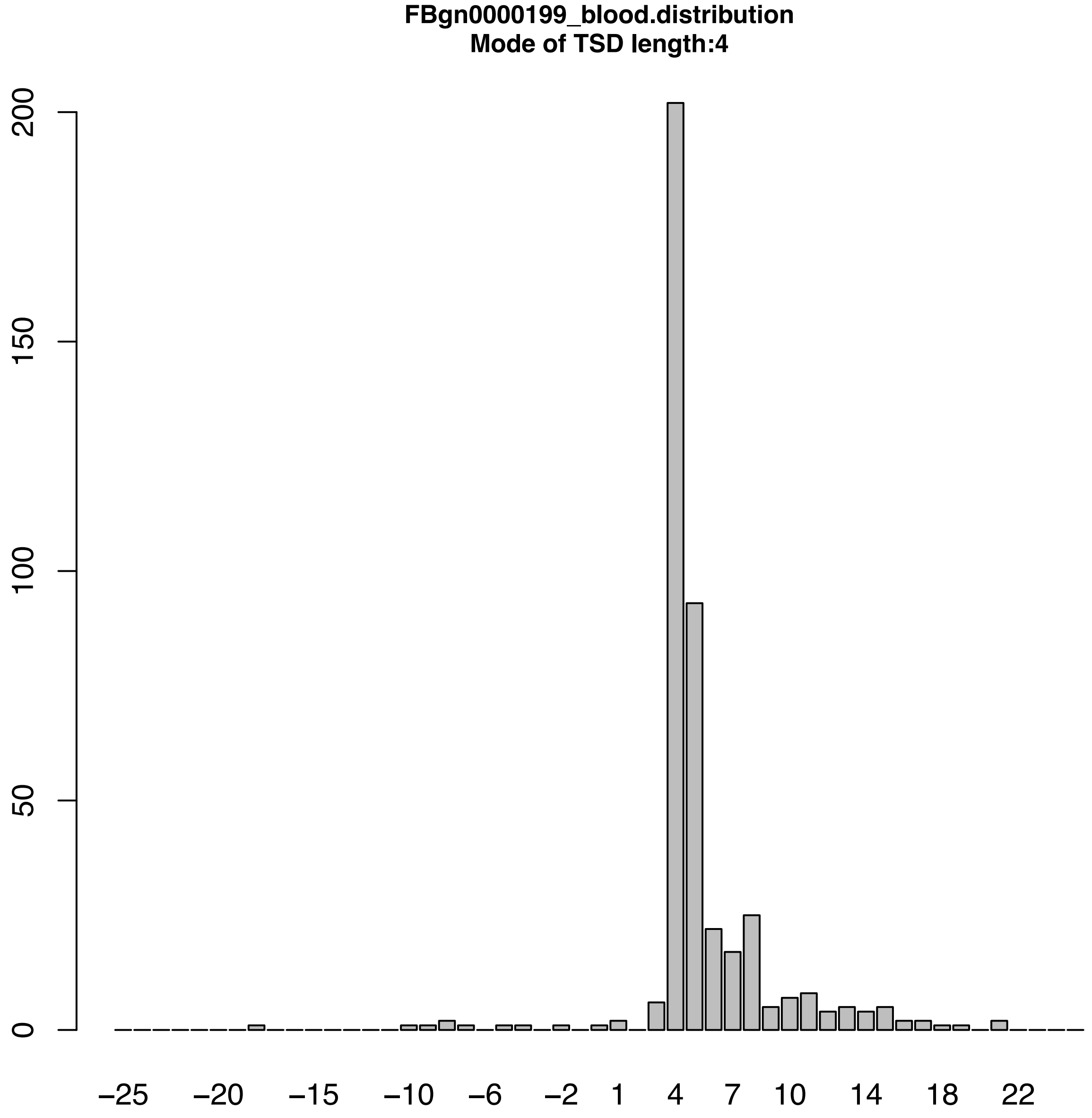
FBgn0000199_blood.distribution
Mode of TSD length:4
0
50
100
150
200
−25
−20
−15
−10
−6
−2
1
4
7
10
14
18
22

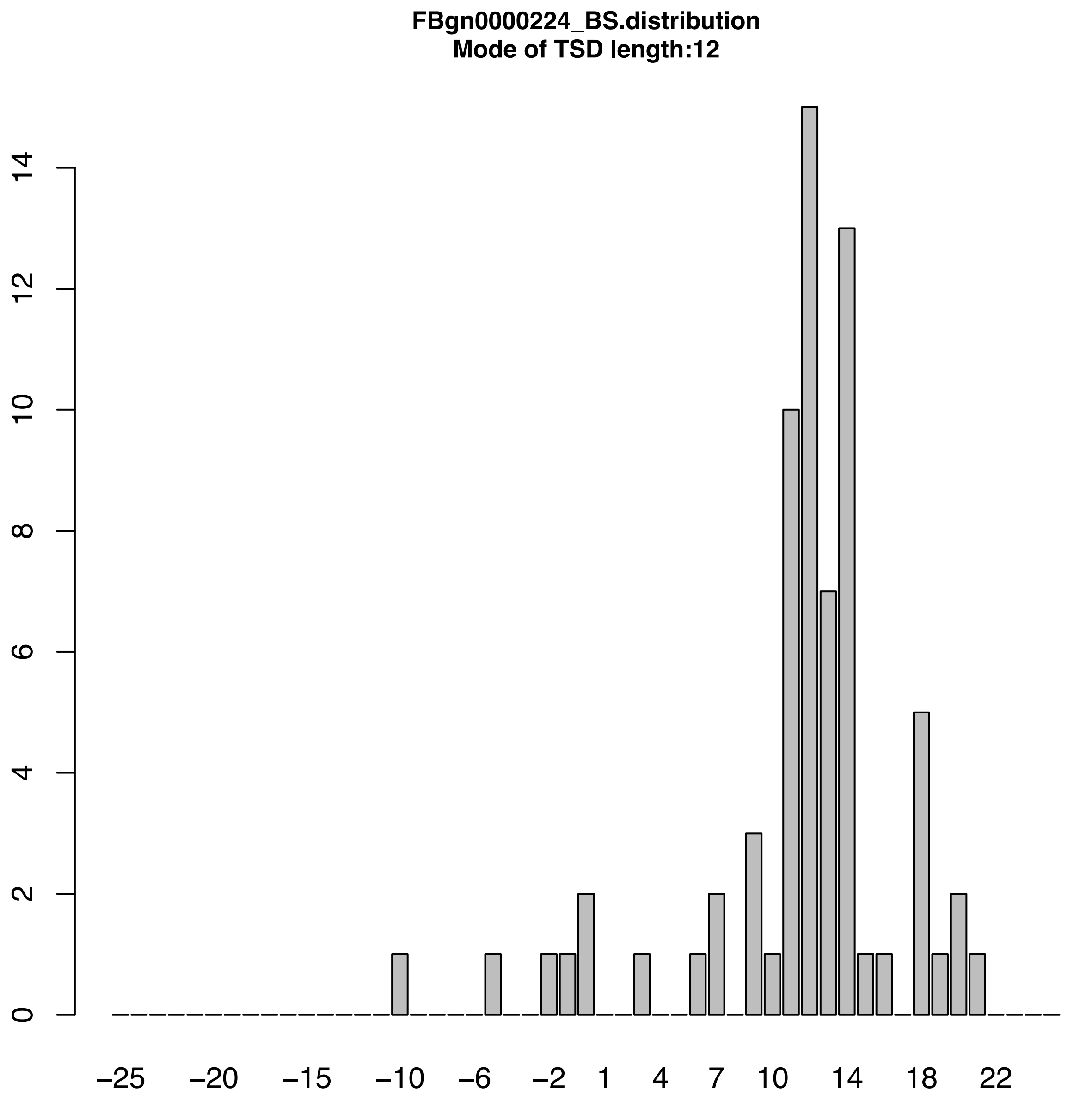
FBgn0000224_BS.distribution
Mode of TSD length:12
0
2
4
6
8
10
12
14
−25
−20
−15
−10
−6
−2
1
4
7
10
14
18
22

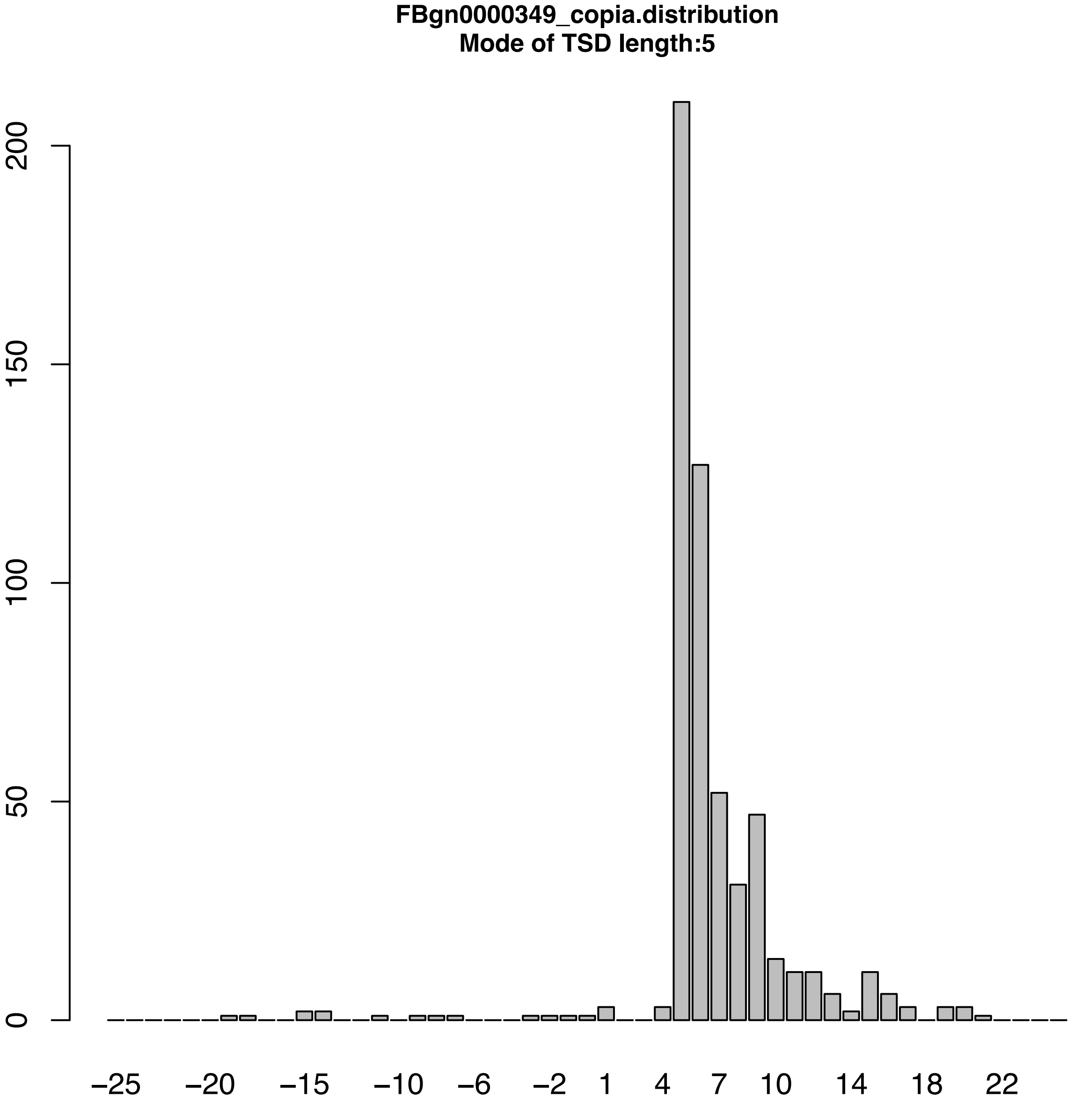
FBgn0000349_copia.distribution
Mode of TSD length:5
0
50
100
150
200
−25
−20
−15
−10
−6
−2
1
4
7
10
14
18
22

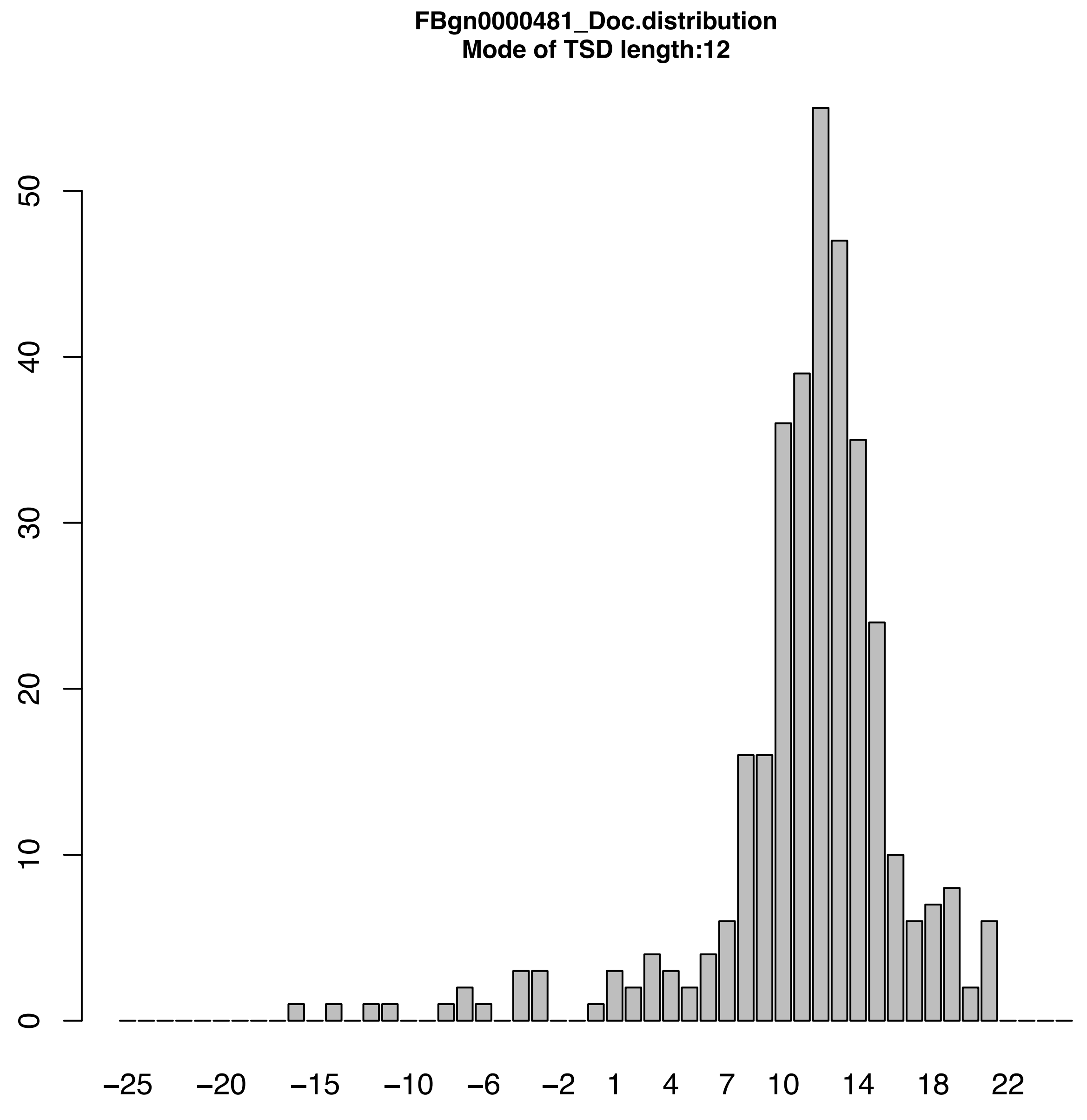
FBgn0000481_Doc.distribution
Mode of TSD length:12
0
10
20
30
40
50
−25
−20
−15
−10
−6
−2
1
4
7
10
14
18
22

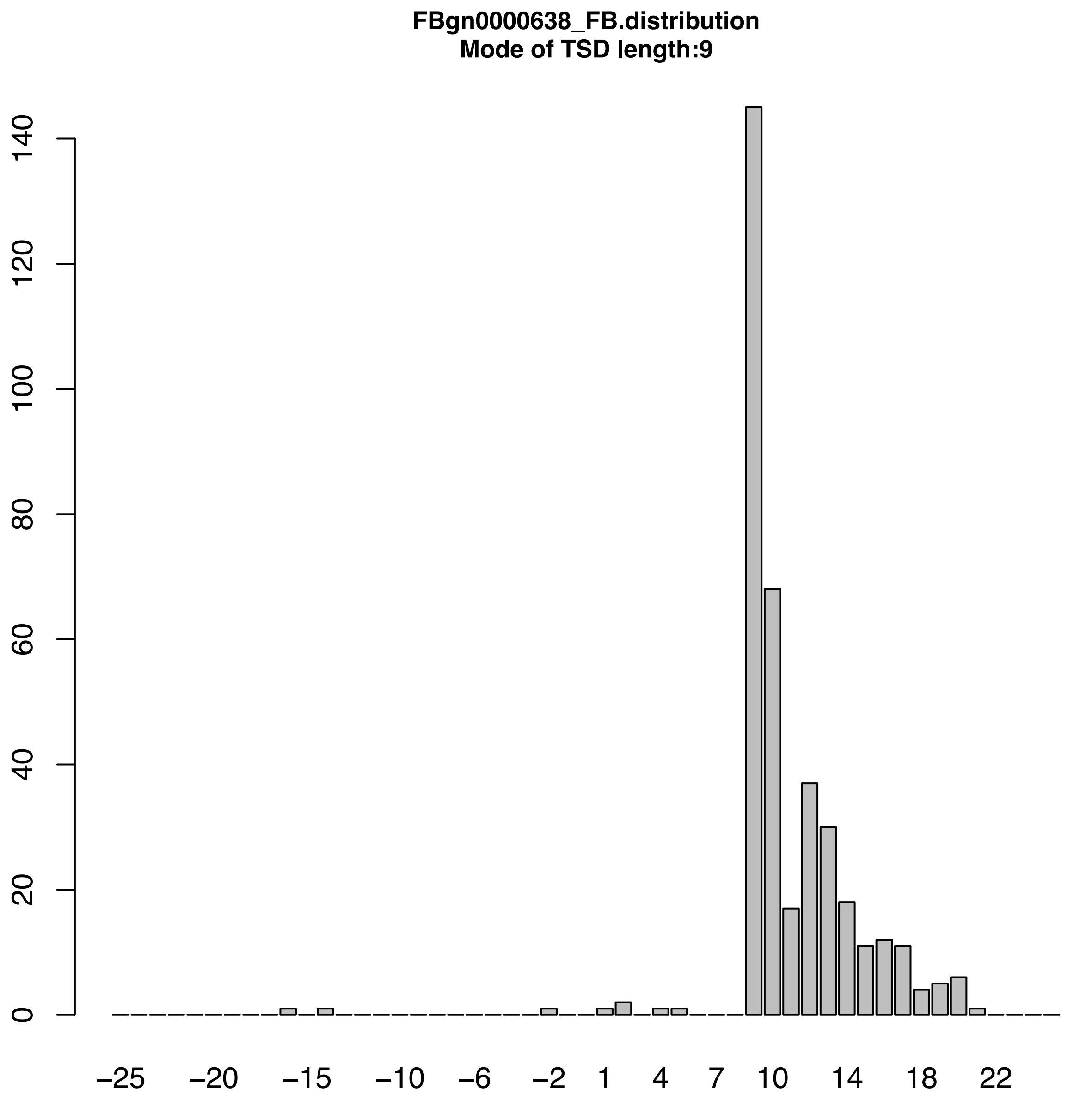
FBgn0000638_FB.distribution
Mode of TSD length:9
0
20
40
60
80
100
120
140
−25
−20
−15
−10
−6
−2
1
4
7
10
14
18
22

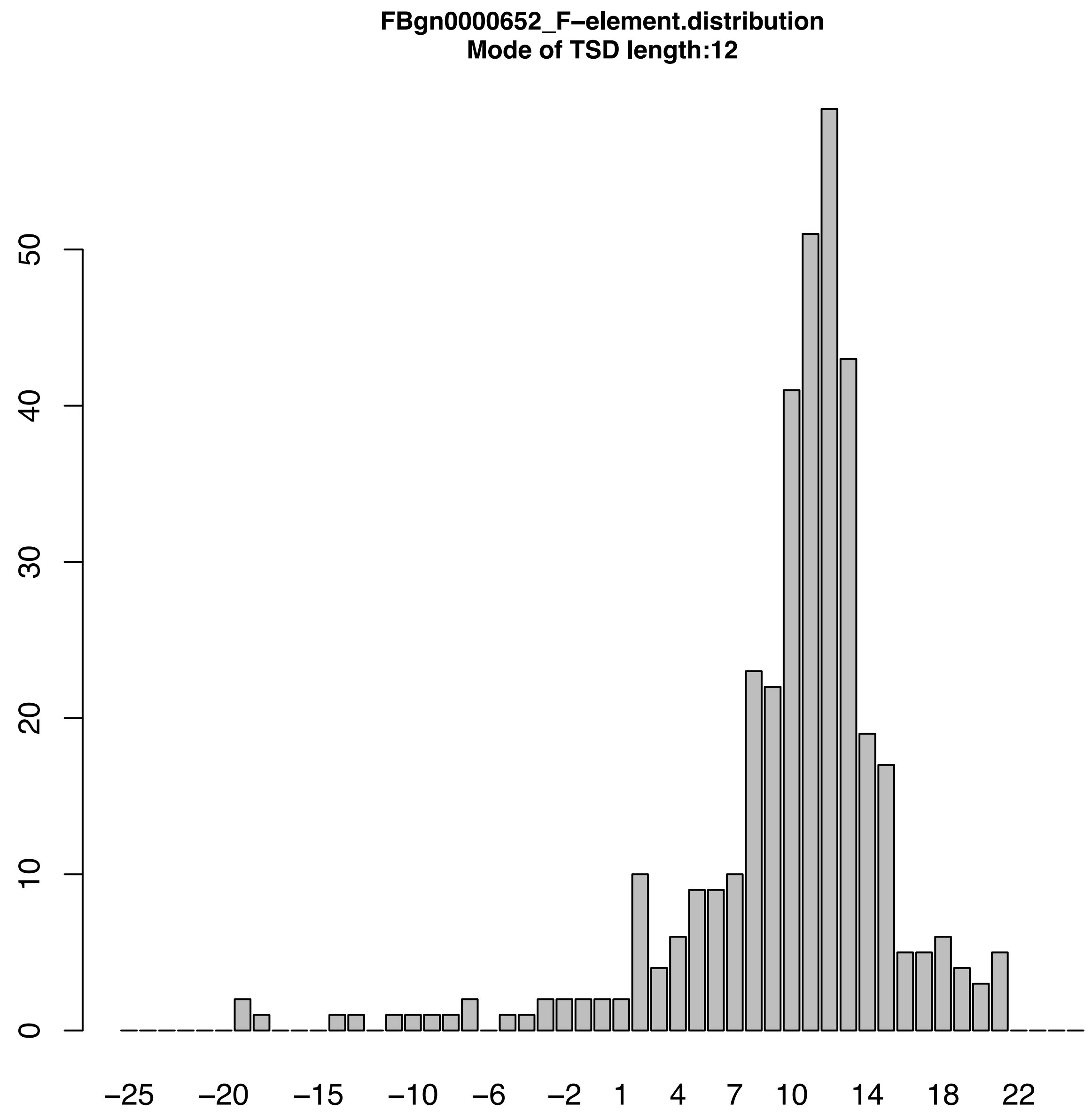
FBgn0000652_F−element.distribution
Mode of TSD length:12
0
10
20
30
40
50
−25
−20
−15
−10
−6
−2
1
4
7
10
14
18
22

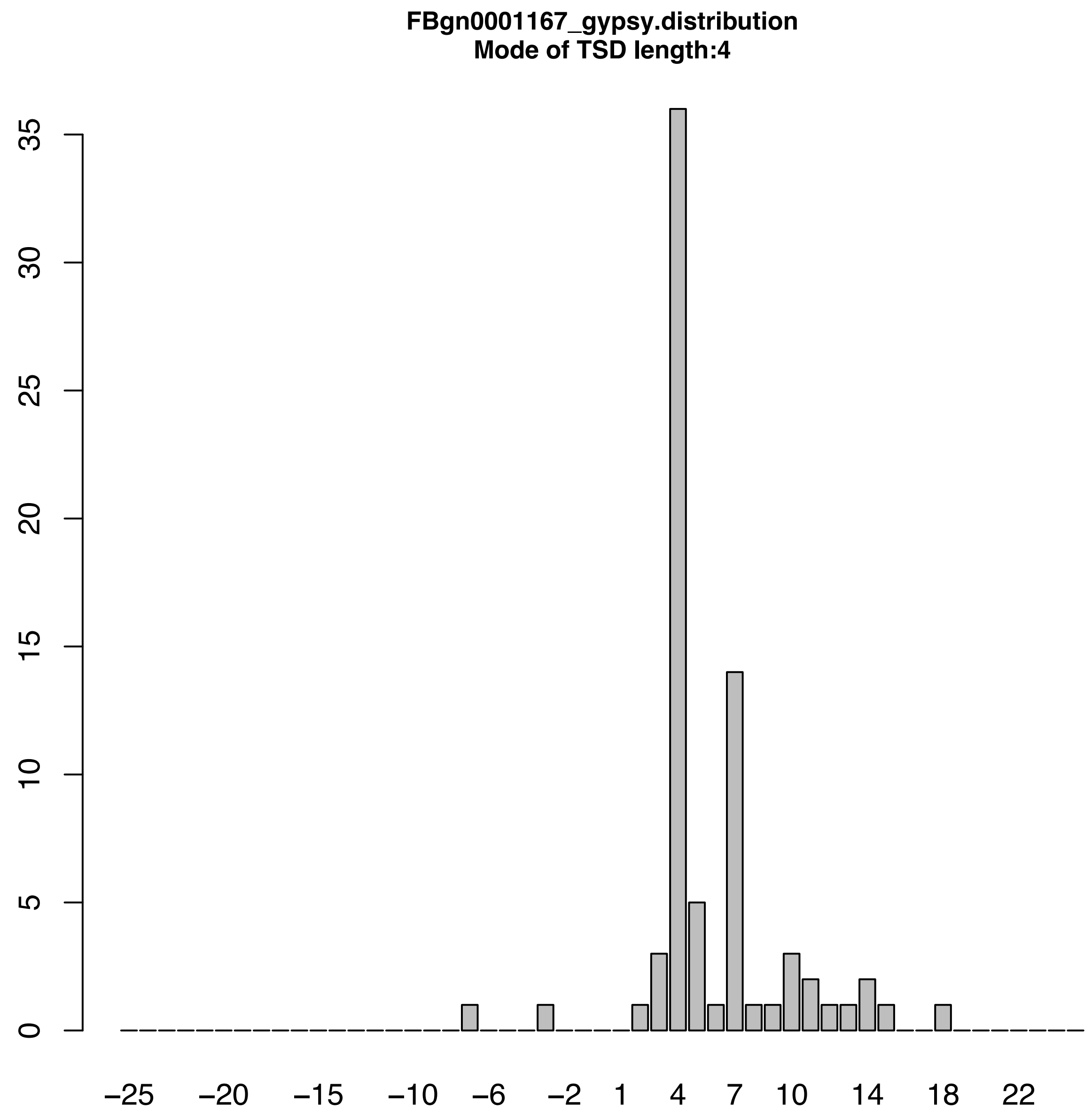
FBgn0001167_gypsy.distribution
Mode of TSD length:4
0
5
10
15
20
25
30
35
−25
−20
−15
−10
−6
−2
1
4
7
10
14
18
22

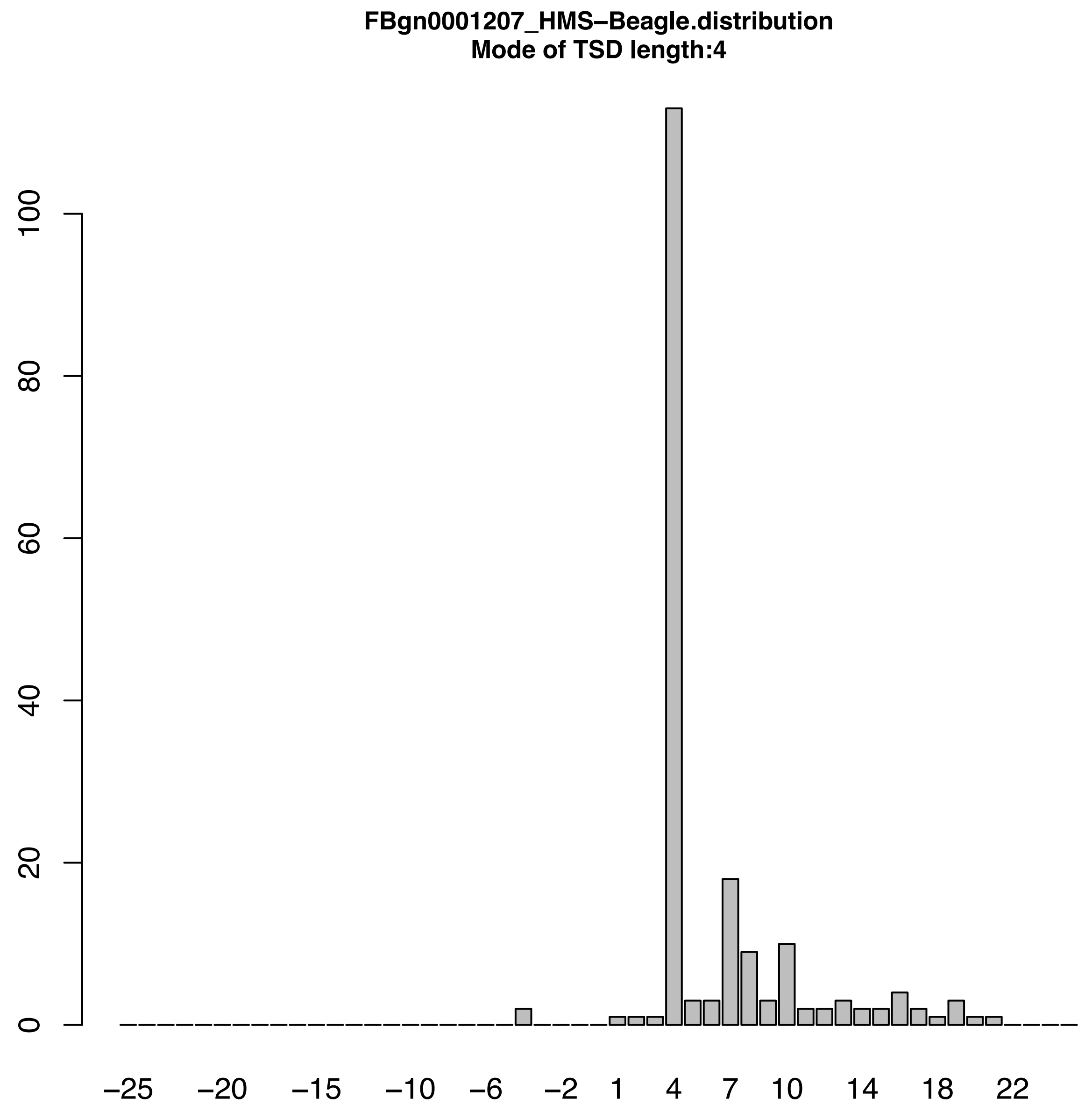
FBgn0001207_HMS−Beagle.distribution
Mode of TSD length:4
0
20
40
60
80
100
−25
−20
−15
−10
−6
−2
1
4
7
10
14
18
22

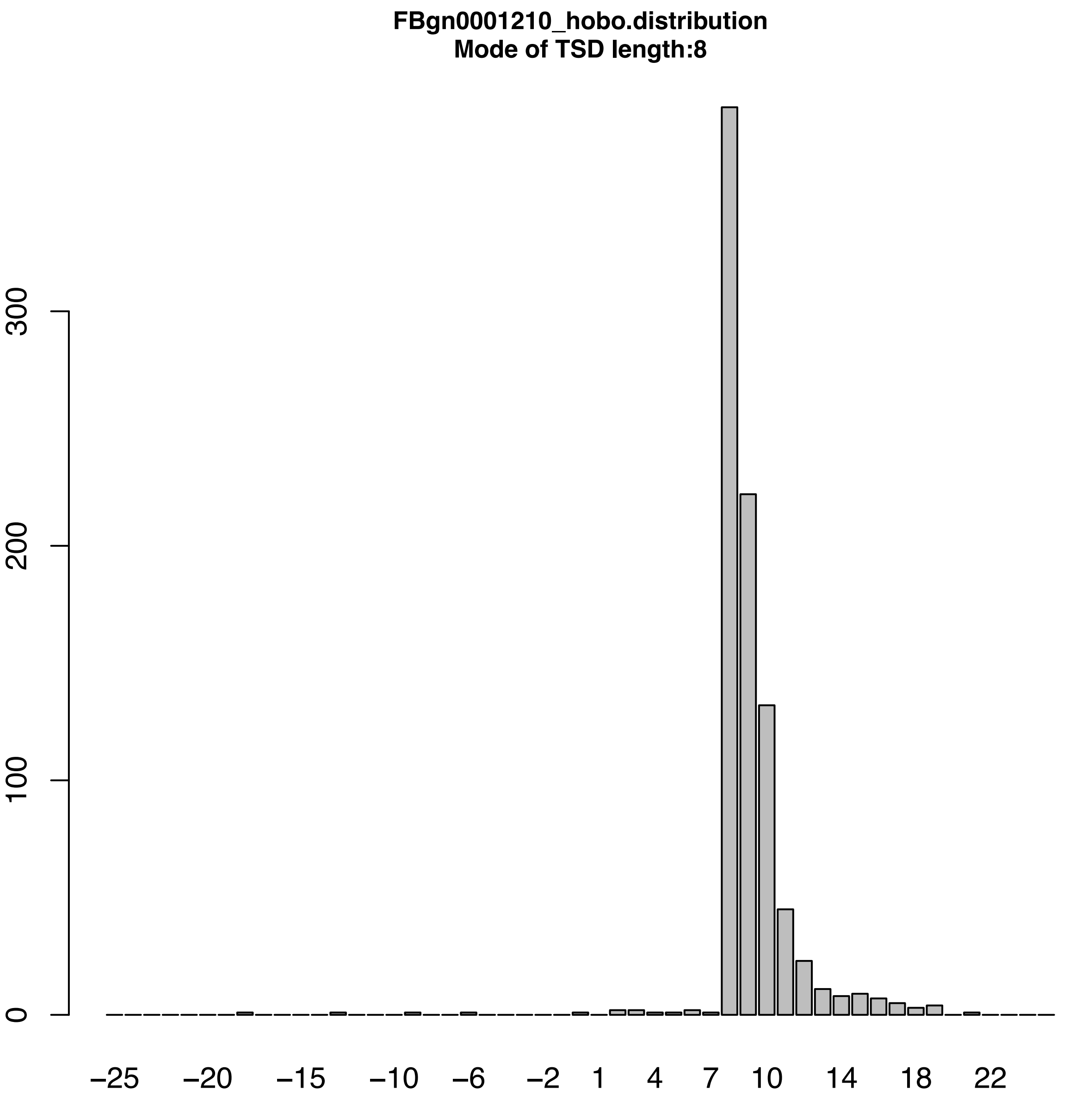
FBgn0001210_hobo.distribution
Mode of TSD length:8
300
200
100
0
−25
−20
−15
−10
−6
−2
1
4
7
10
14
18
22

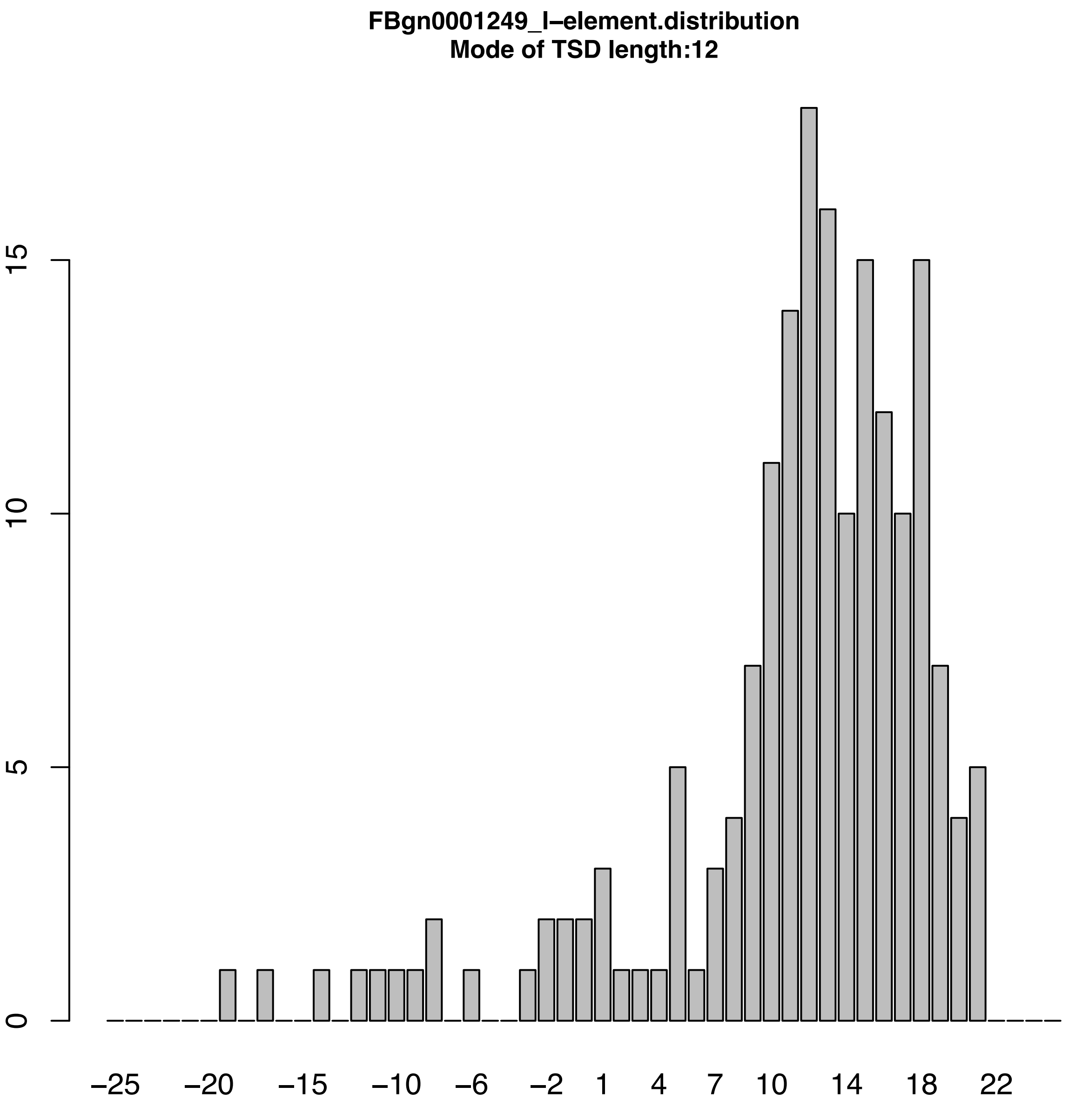

FBgn0001249_I–element.distribution
Mode of TSD length:12
0
5
10
15
–25
–20
–15
–10
–6
–2
1
4
7
10
14
18
22

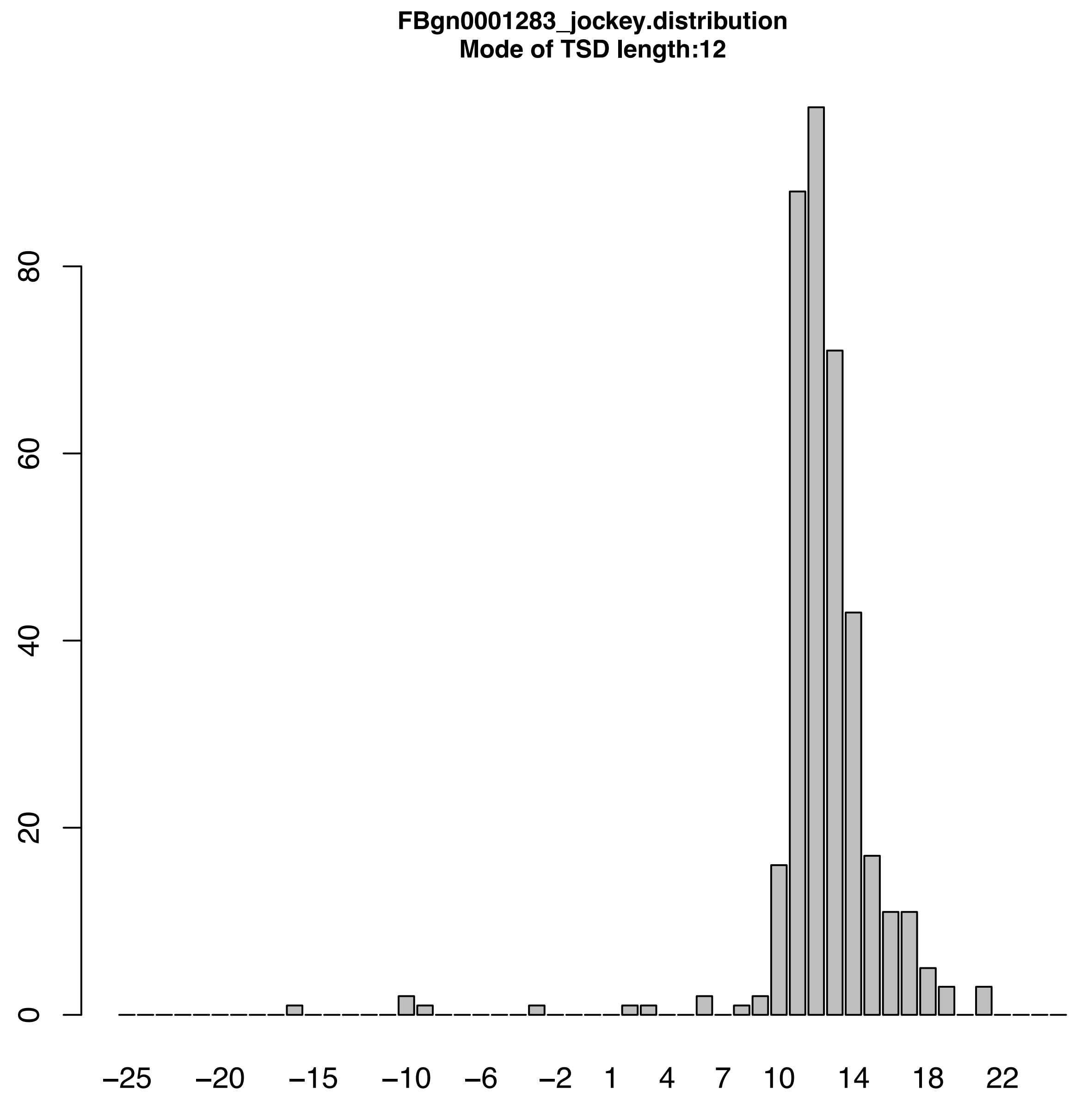
FBgn0001283_jockey.distribution
Mode of TSD length:12
0
20
40
60
80
−25
−20
−15
−10
−6
−2
1
4
7
10
14
18
22

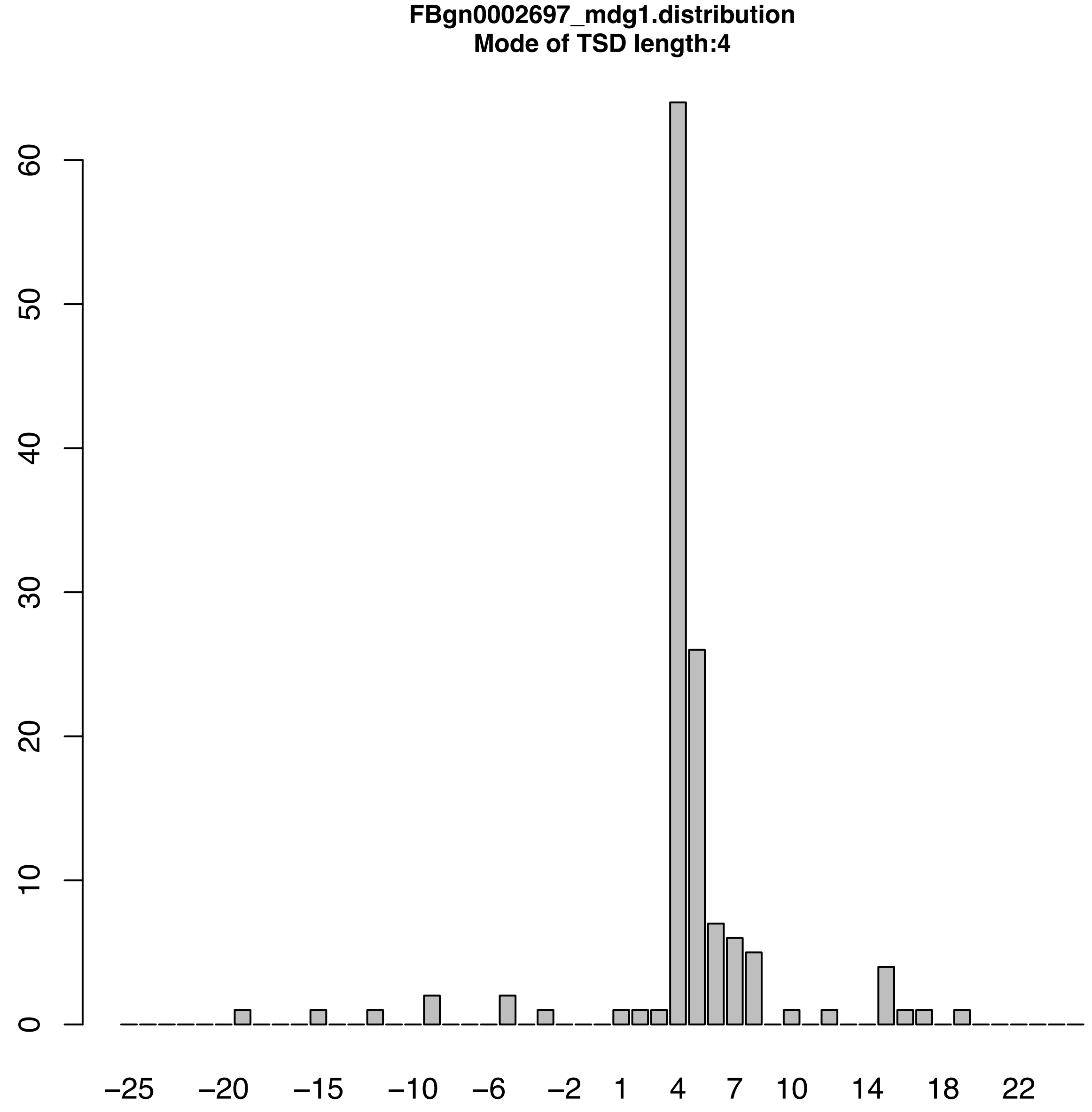
FBgn0002697_mdg1.distribution
Mode of TSD length:4
0
10
20
30
40
50
60
−25
−20
−15
−10
−6
−2
1
4
7
10
14
18
22

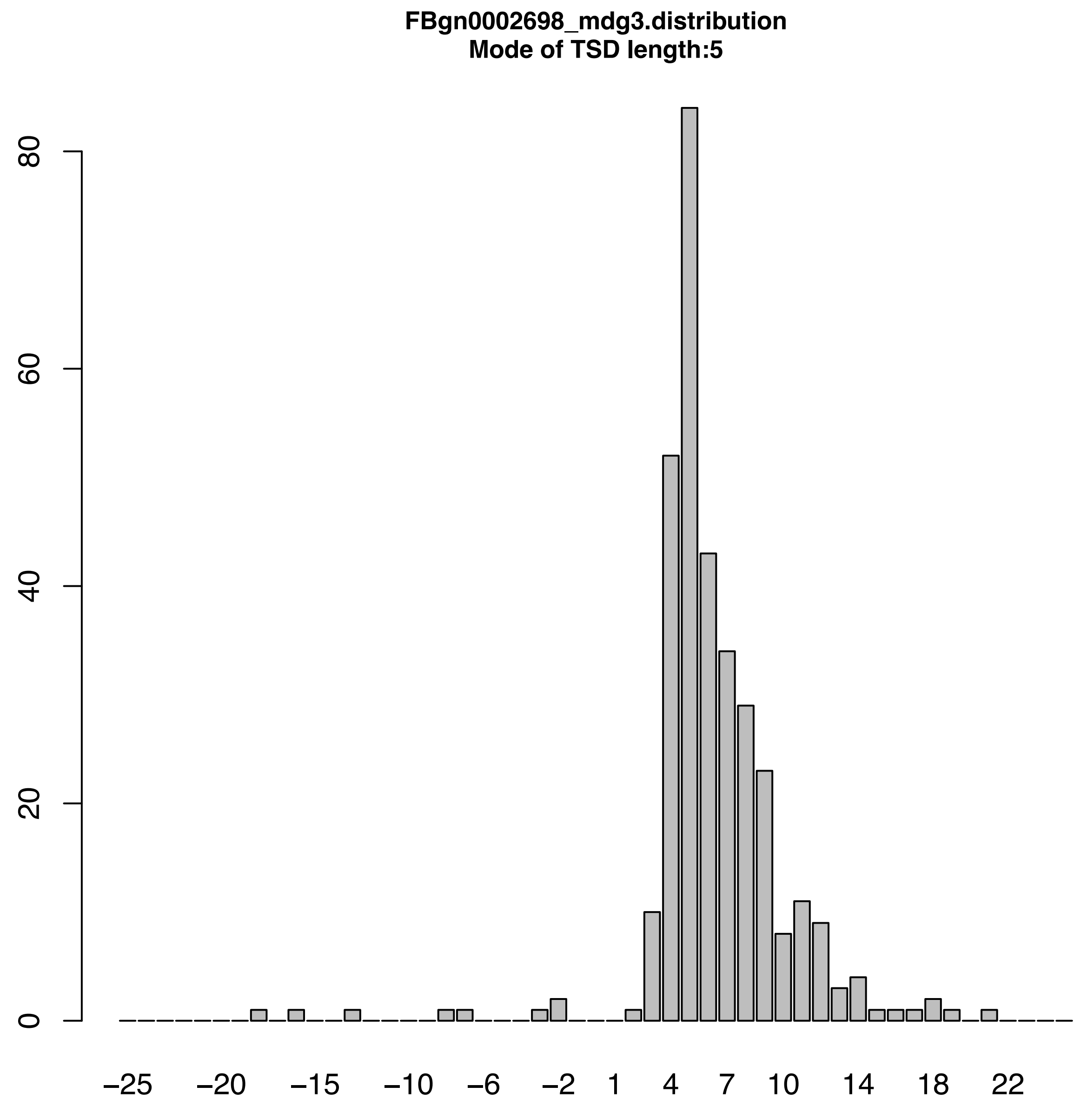
FBgn0002698_mdg3.distribution
Mode of TSD length:5
0
20
40
60
80
−25
−20
−15
−10
−6
−2
1
4
7
10
14
18
22

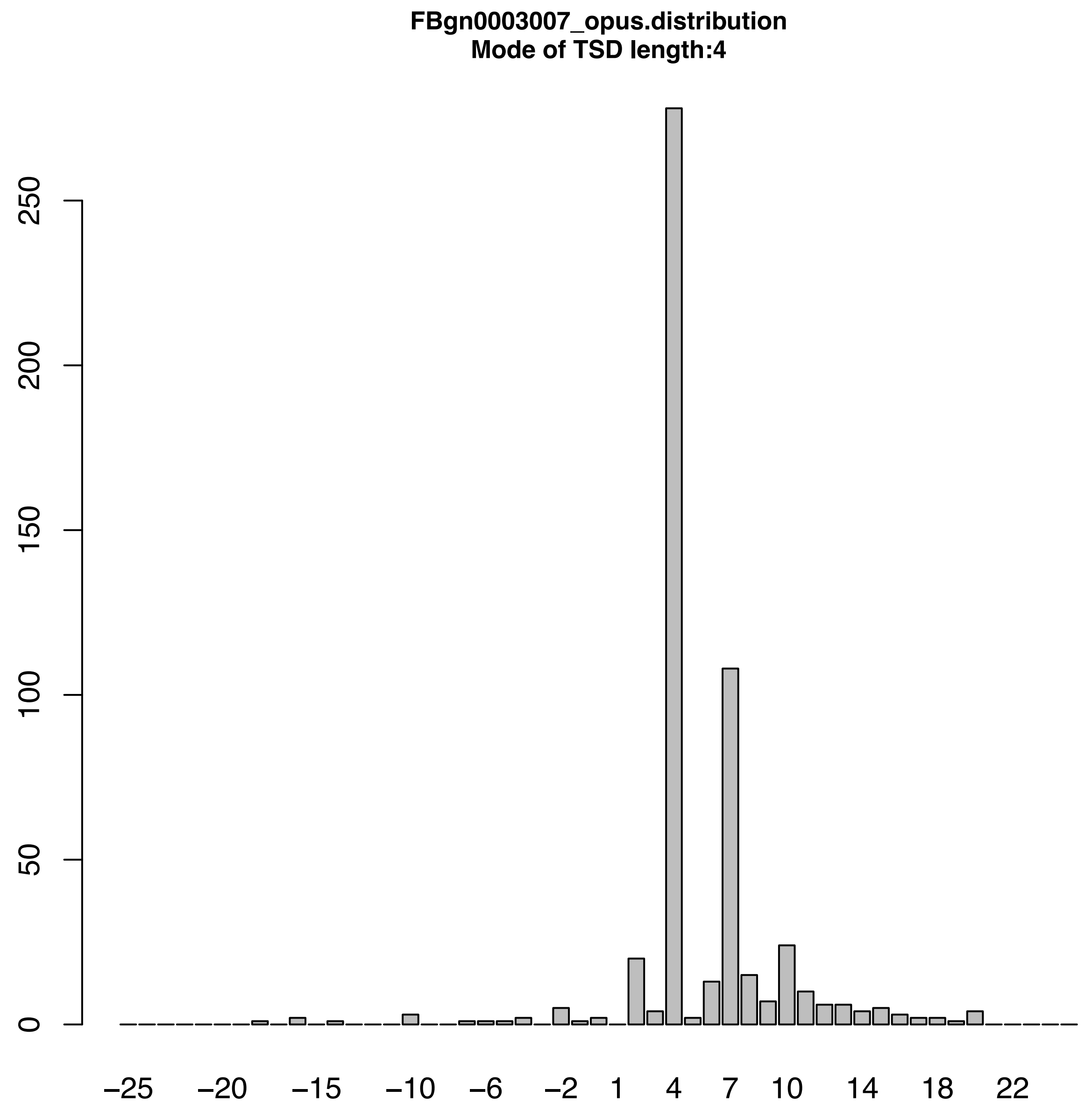

FBgn0003007_opus.distribution
Mode of TSD length:4
0
50
100
150
200
250
−25
−20
−15
−10
−6
−2
1
4
7
10
14
18
22

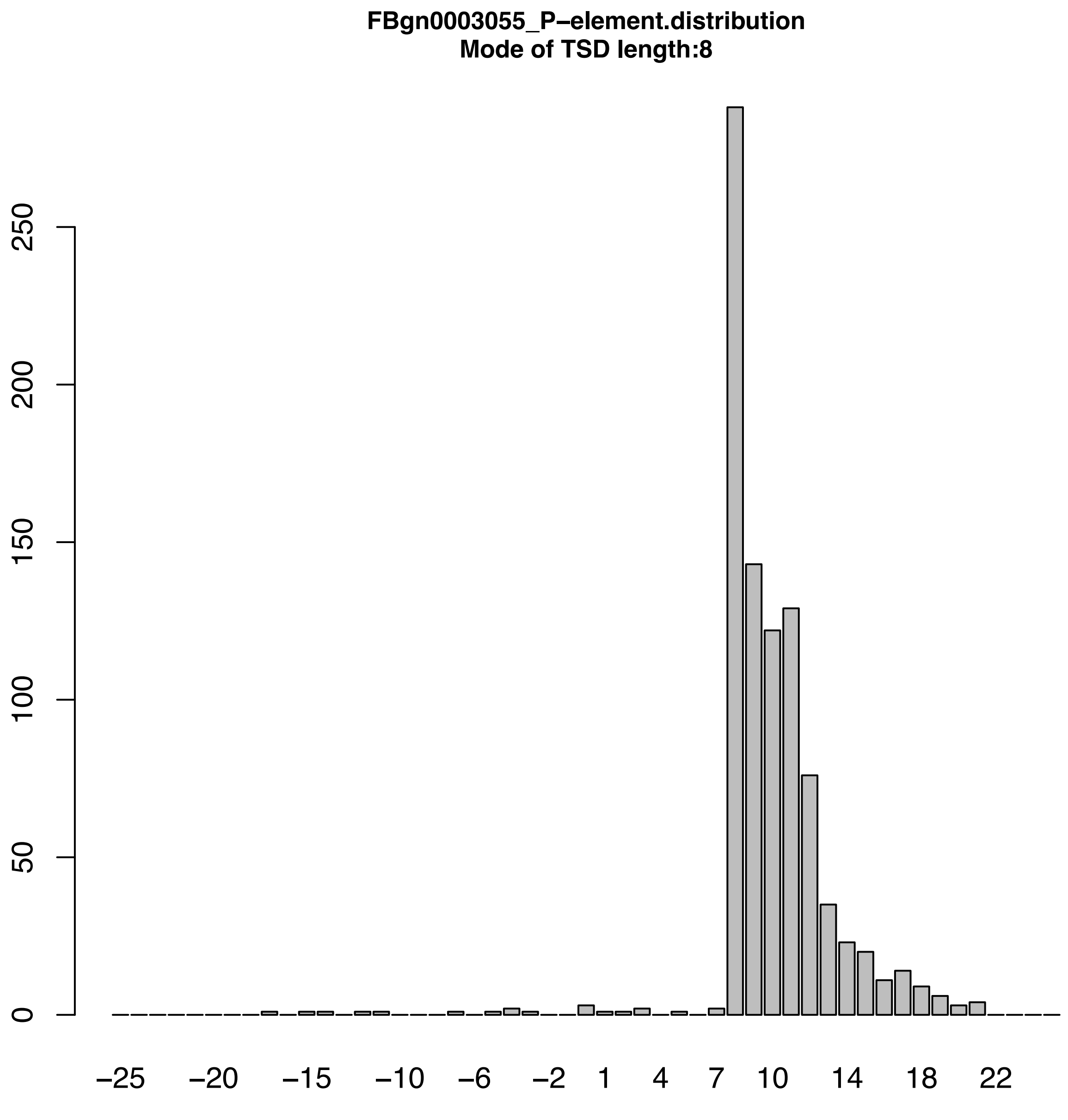

FBgn0003055_P–element.distribution
Mode of TSD length:8
0
50
100
150
200
250
–25
–20
–15
–10
–6
–2
1
4
7
10
14
18
22

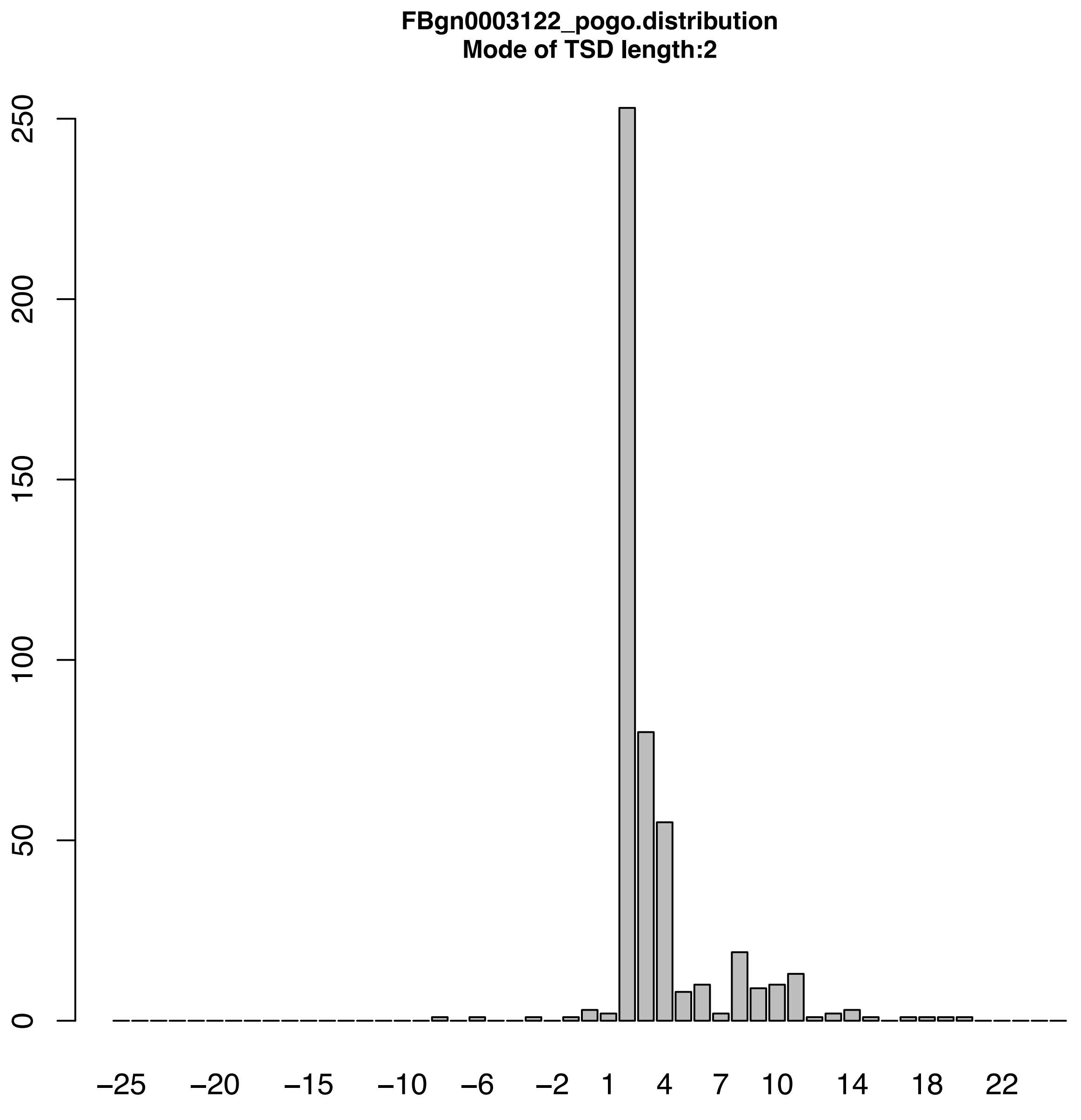
FBgn0003122_pogo.distribution
Mode of TSD length:2
0
50
100
150
200
250
−25
−20
−15
−10
−6
−2
1
4
7
10
14
18
22

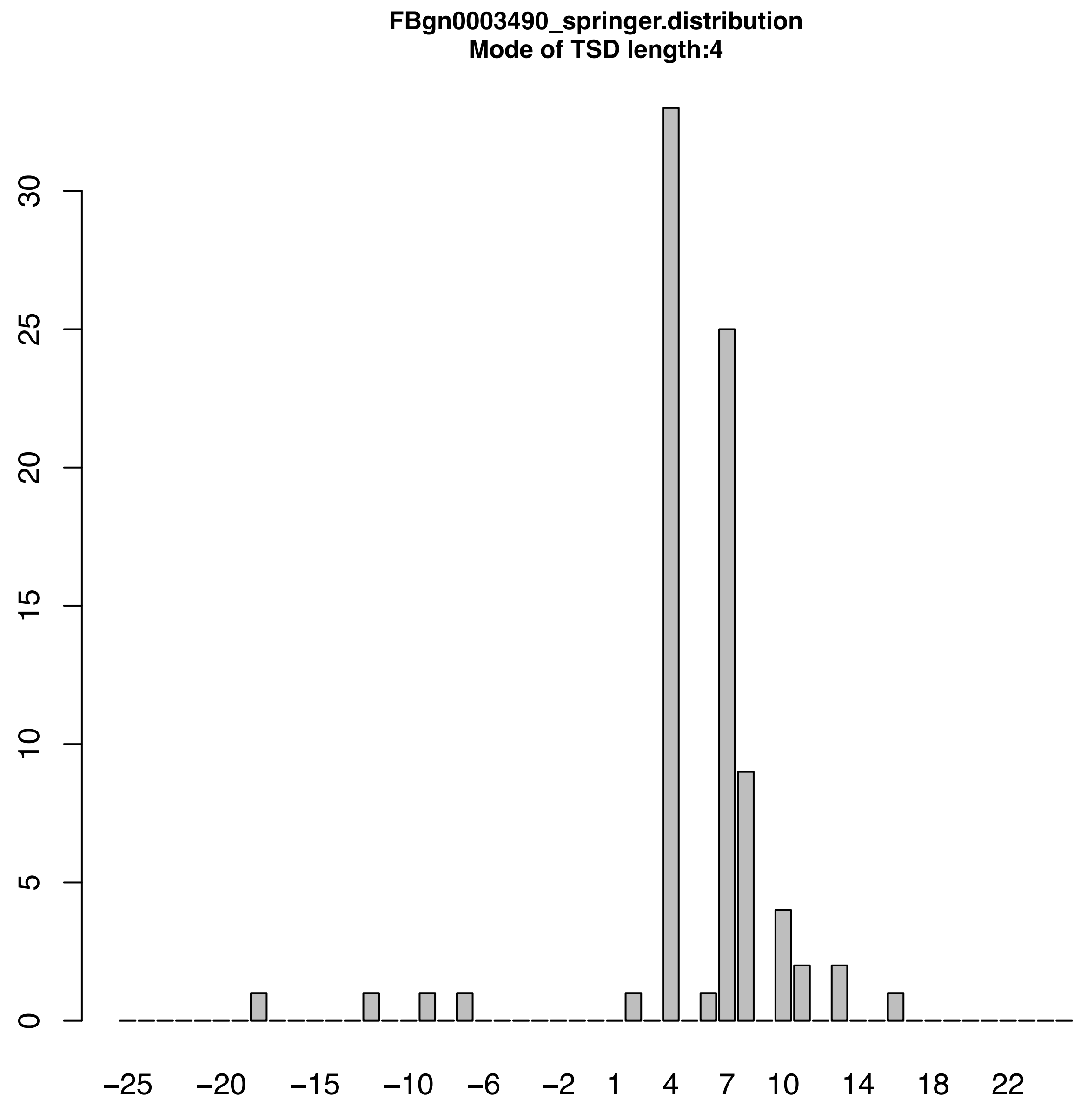

FBgn0003490_springer.distribution
Mode of TSD length:4
0
5
10
15
20
25
30
−25
−20
−15
−10
−6
−2
1
4
7
10
14
18
22

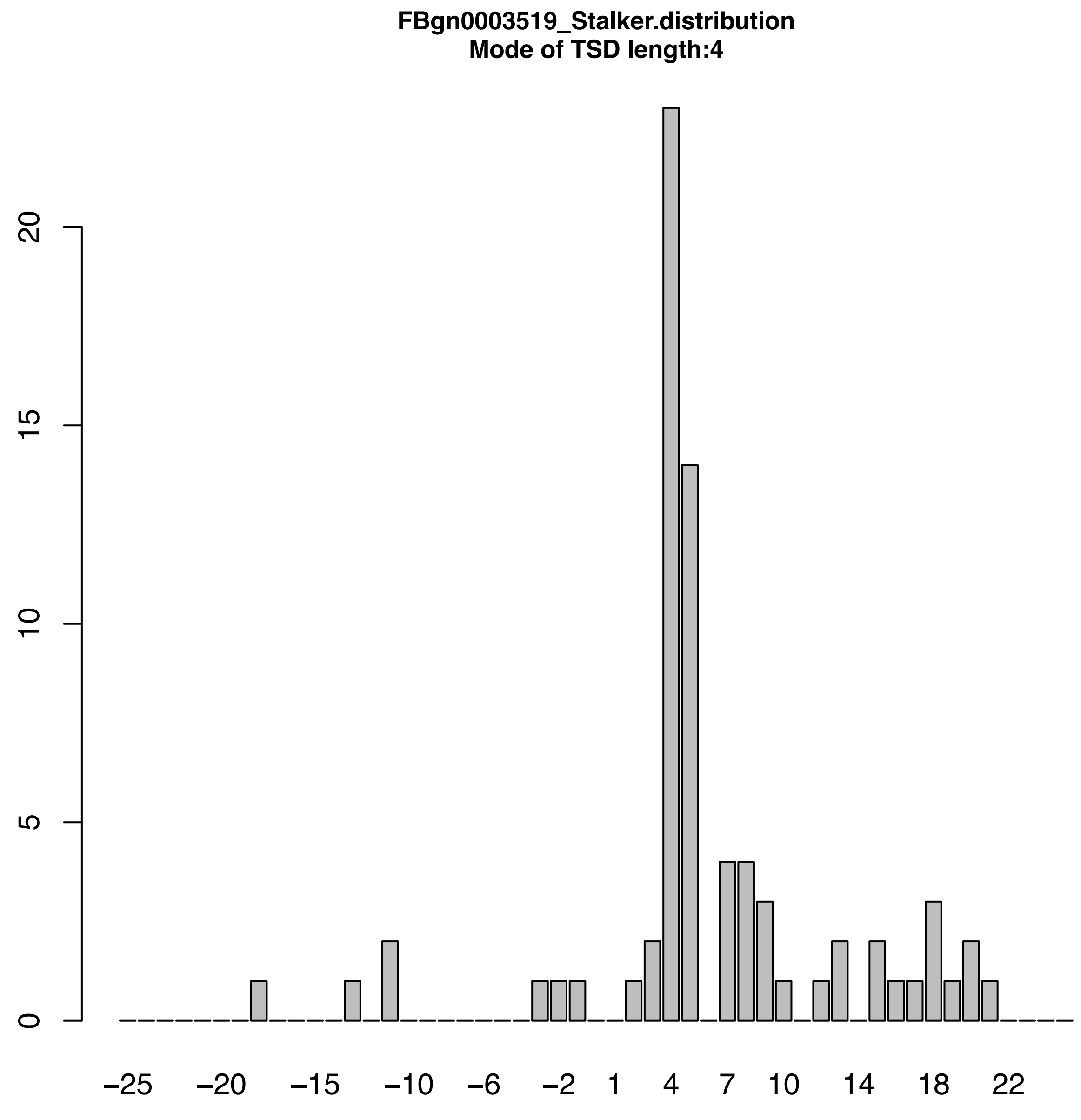
FBgn0003519_Stalker.distribution
Mode of TSD length:4
0
5
10
15
20
−25
−20
−15
−10
−6
−2
1
4
7
10
14
18
22

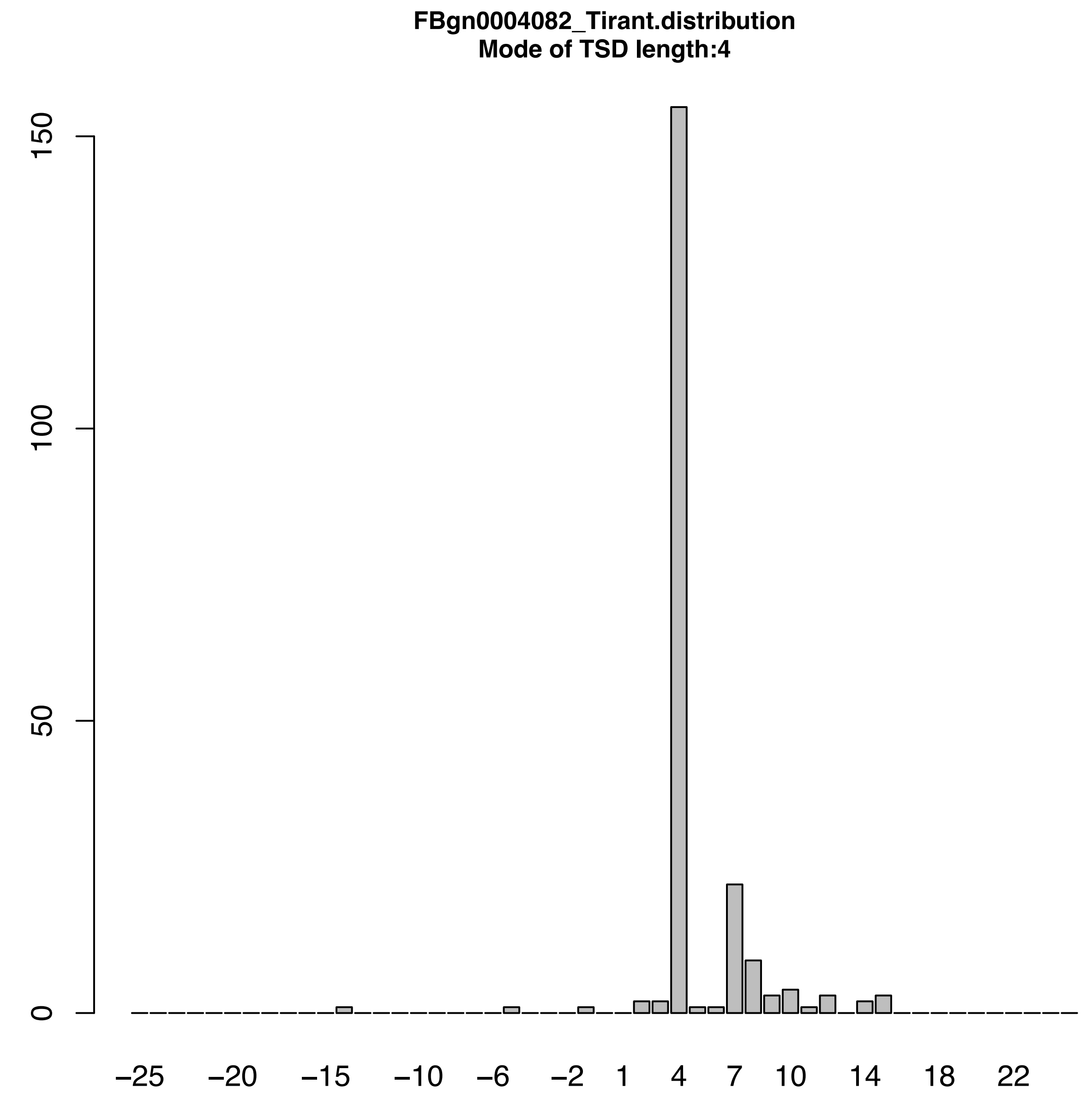
FBgn0004082_Tirant.distribution
Mode of TSD length:4
150
100
50
0
−25
−20
−15
−10
−6
−2
1
4
7
10
14
18
22

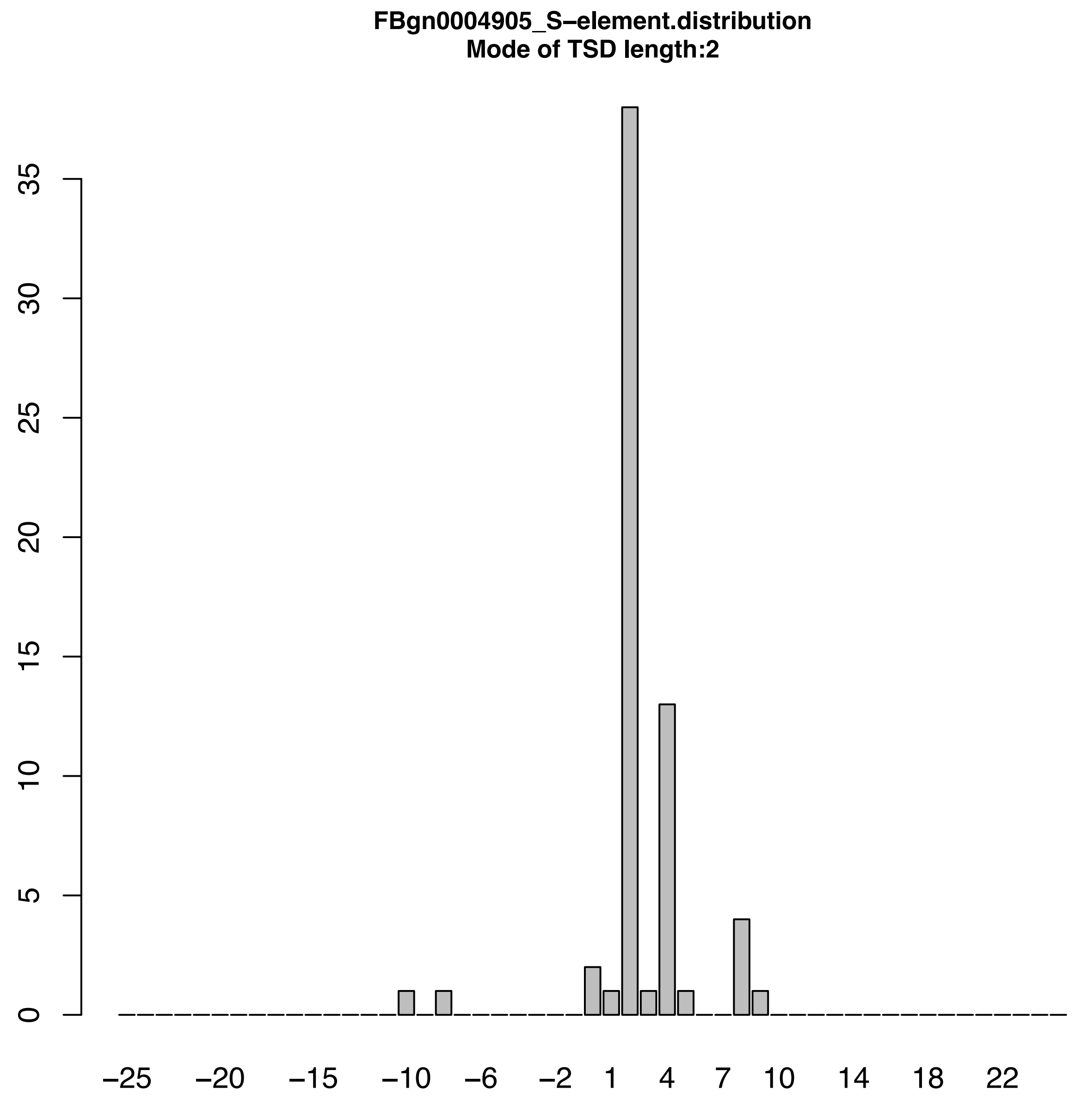
FBgn0004905_S−element.distribution
Mode of TSD length:2
0
5
10
15
20
25
30
35
−25
−20
−15
−10
−6
−2
1
4
7
10
14
18
22

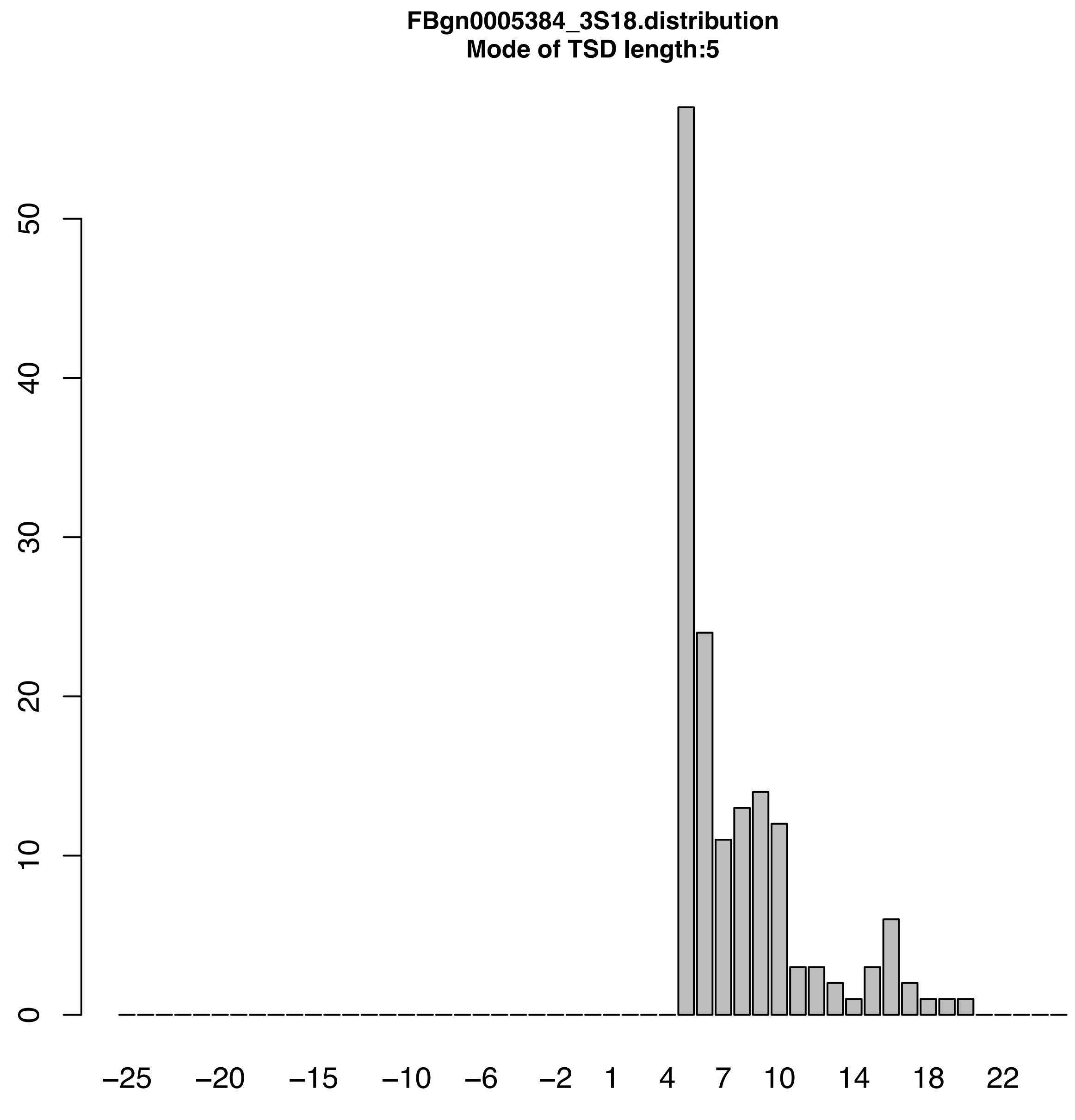
FBgn0005384_3S18.distribution
Mode of TSD length:5
0
10
20
30
40
50
−25
−20
−15
−10
−6
−2
1
4
7
10
14
18
22

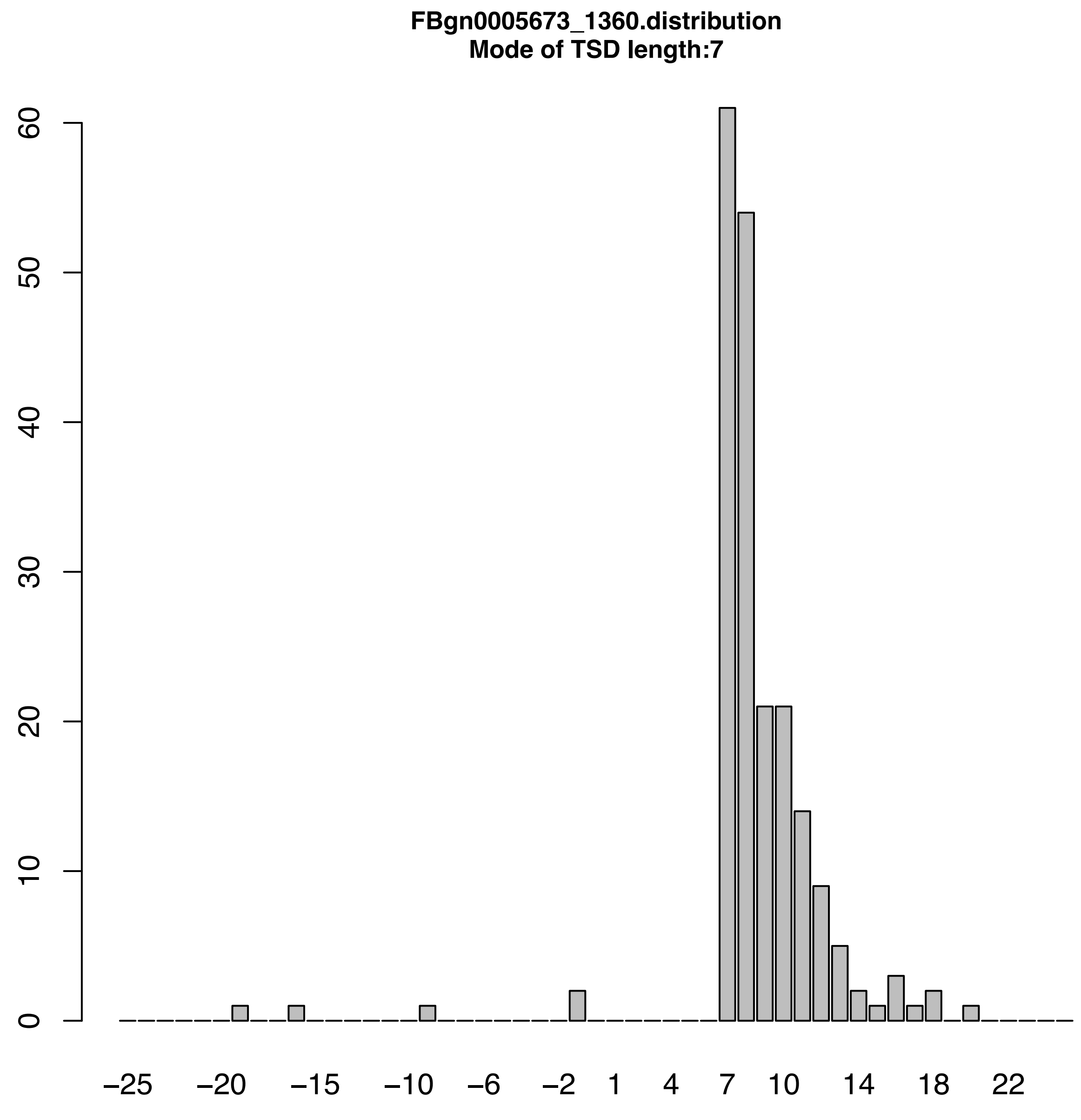

FBgn0005673_1360.distribution
Mode of TSD length:7
0
10
20
30
40
50
60
−25
−20
−15
−10
−6
−2
1
4
7
10
14
18
22

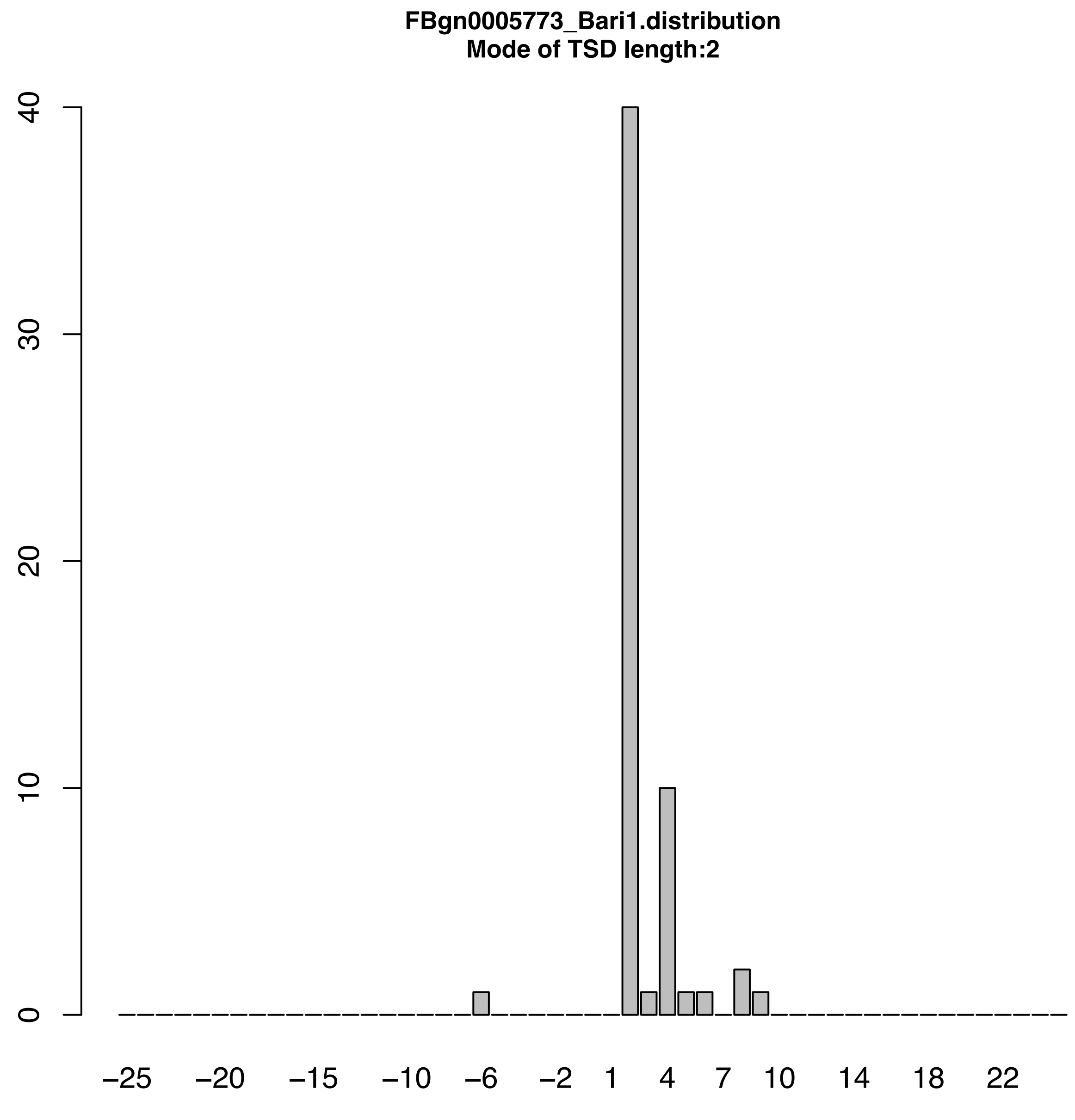
FBgn0005773_Bari1.distribution
Mode of TSD length:2
0
10
20
30
40
−25
−20
−15
−10
−6
−2
1
4
7
10
14
18
22

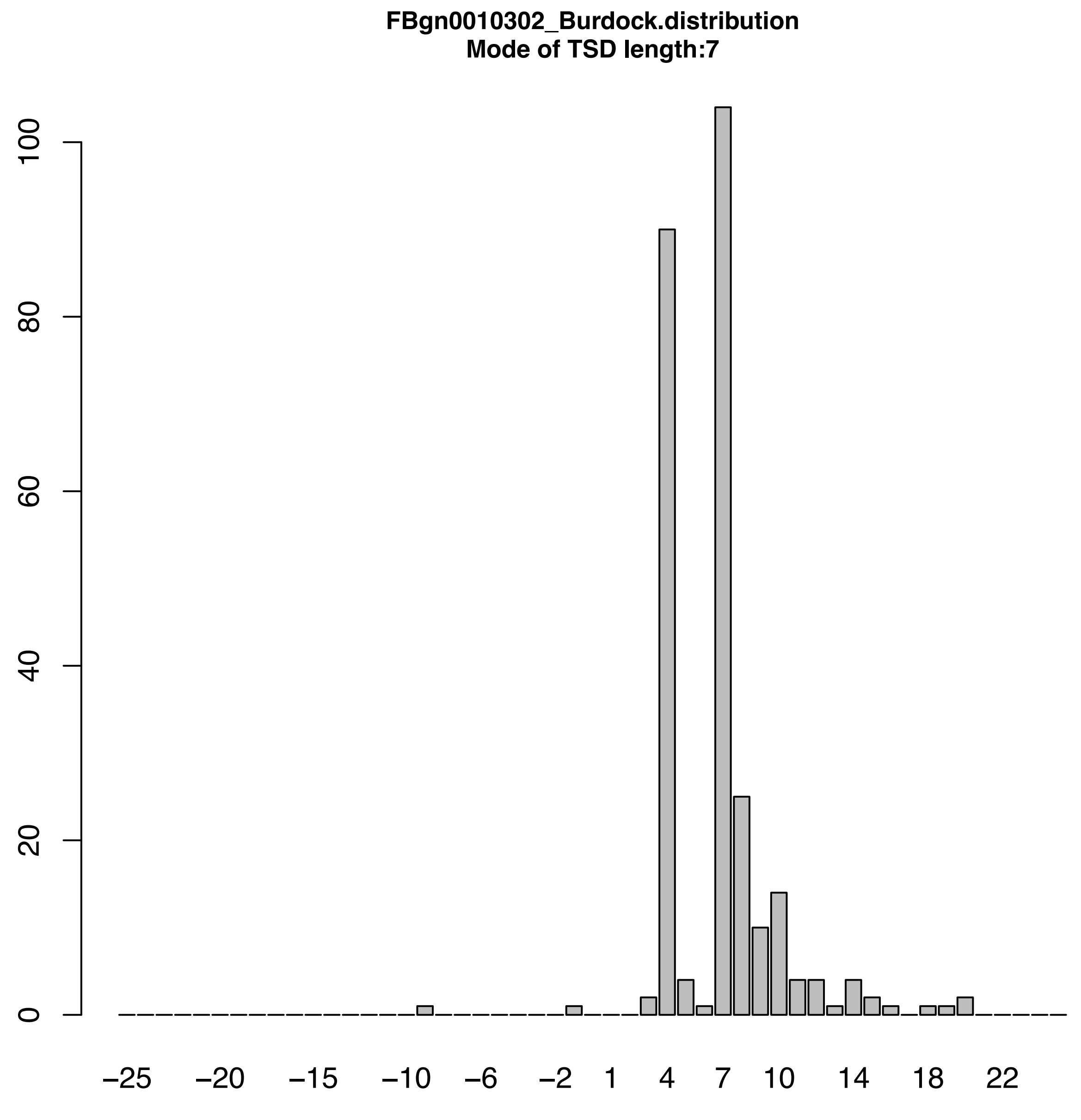
FBgn0010302_Burdock.distribution
Mode of TSD length:7
0
20
40
60
80
100
−25
−20
−15
−10
−6
−2
1
4
7
10
14
18
22

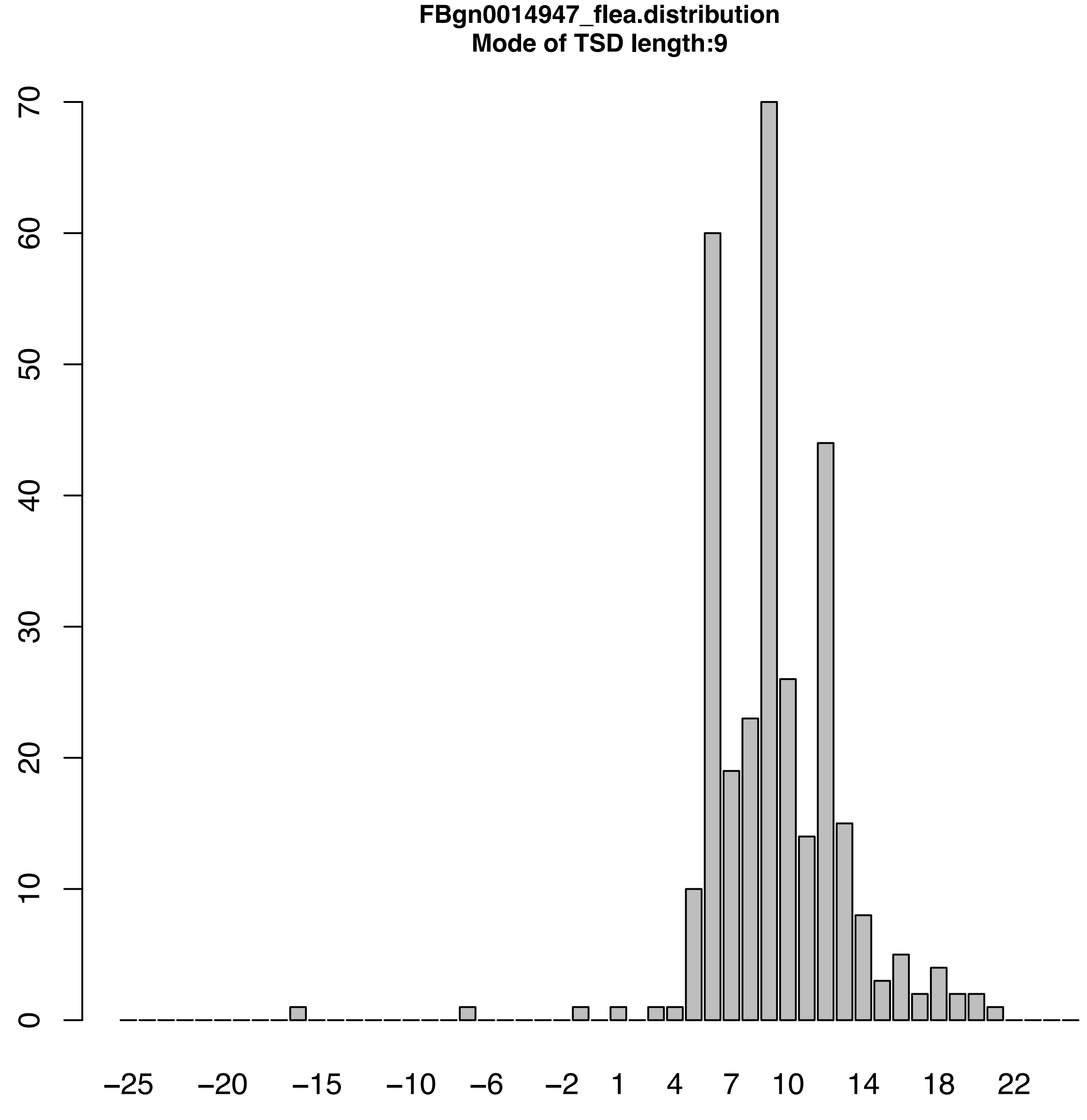

FBgn0014947_flea.distribution
Mode of TSD length:9
0
10
20
30
40
50
60
70
−25
−20
−15
−10
−6
−2
1
4
7
10
14
18
22

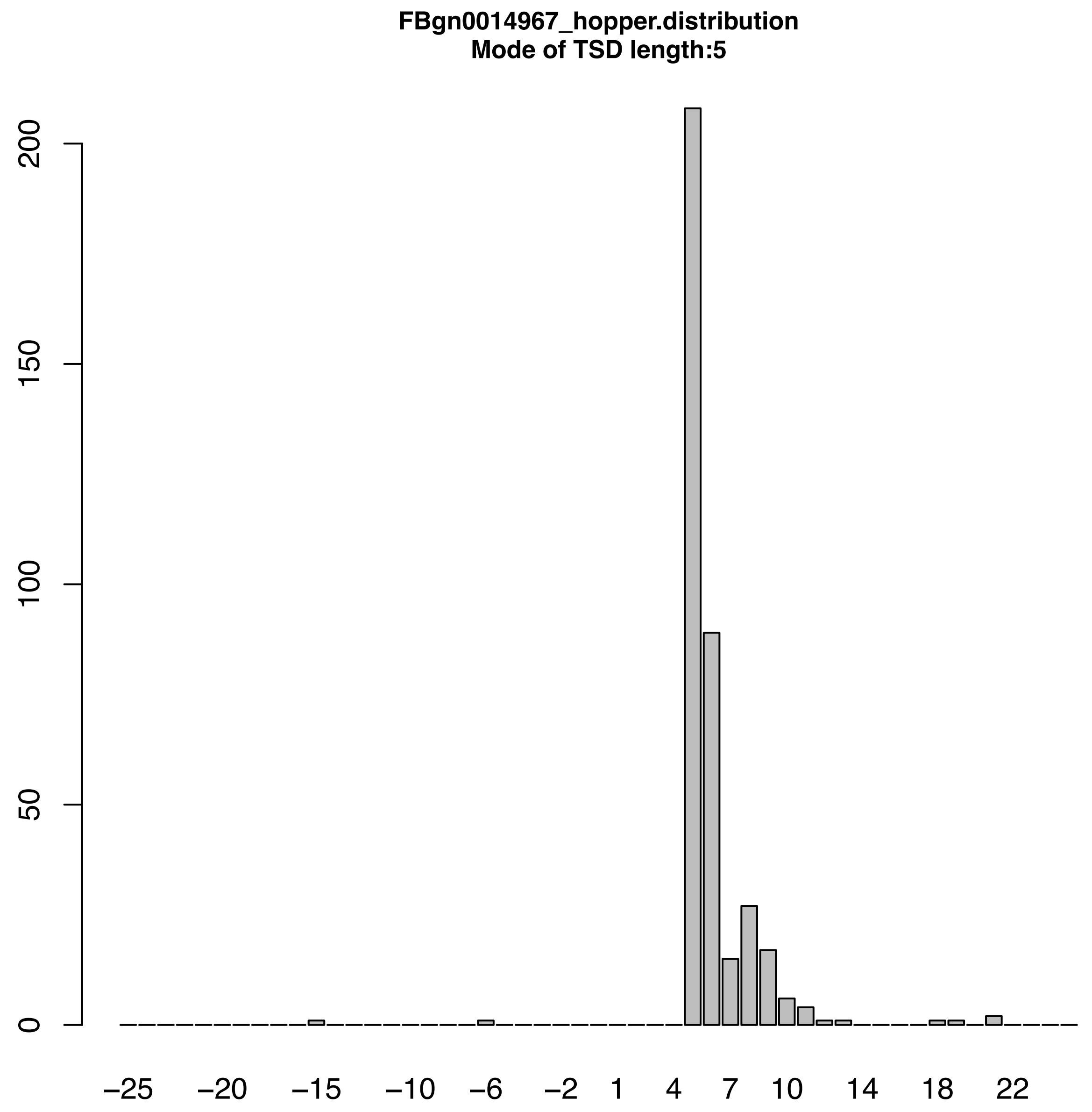
FBgn0014967_hopper.distribution
Mode of TSD length:5
0
50
100
150
200
−25
−20
−15
−10
−6
−2
1
4
7
10
14
18
22

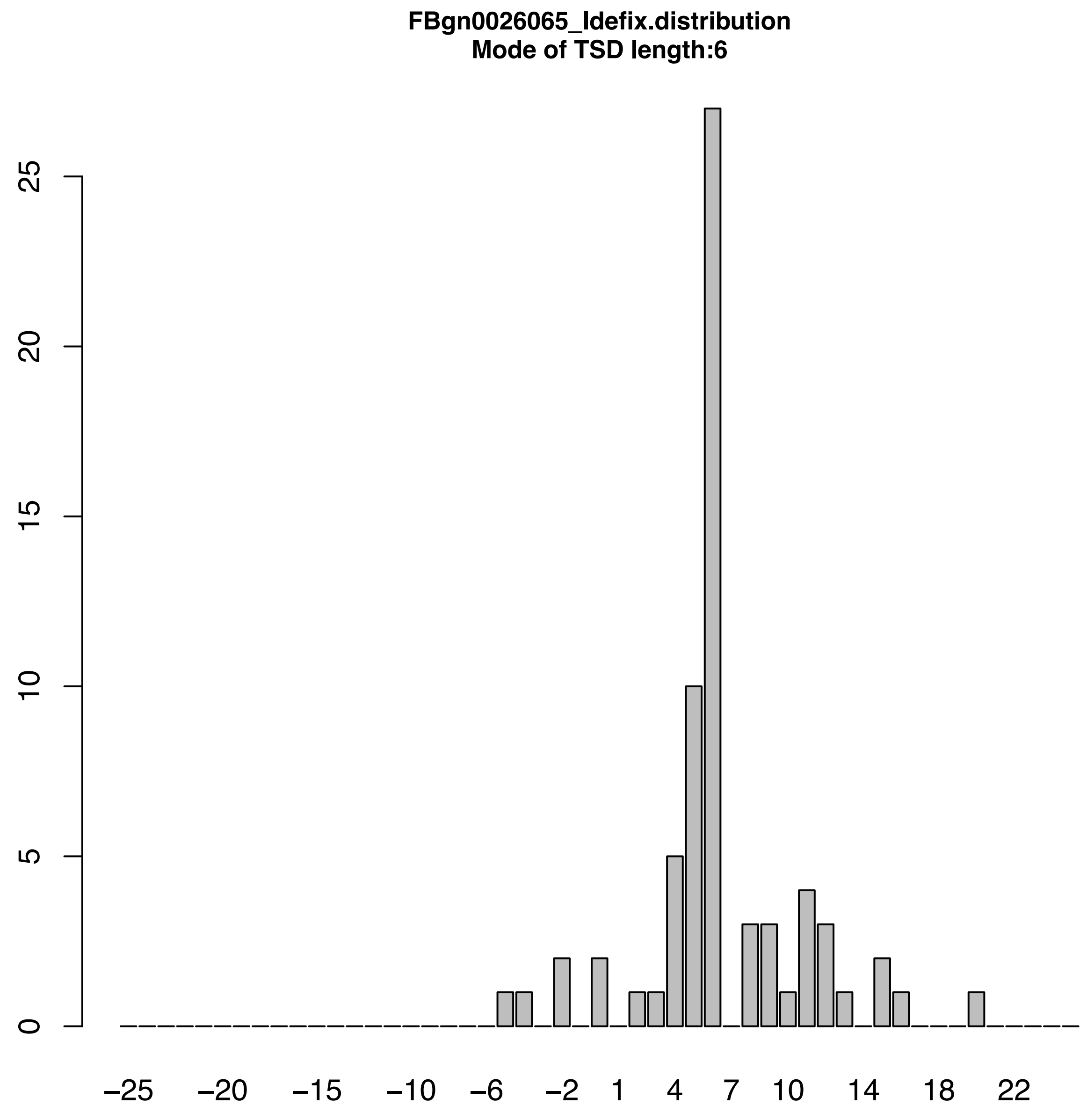

FBgn0026065_Idefix.distribution
Mode of TSD length:6
0
5
10
15
20
25
−25
−20
−15
−10
−6
−2
1
4
7
10
14
18
22

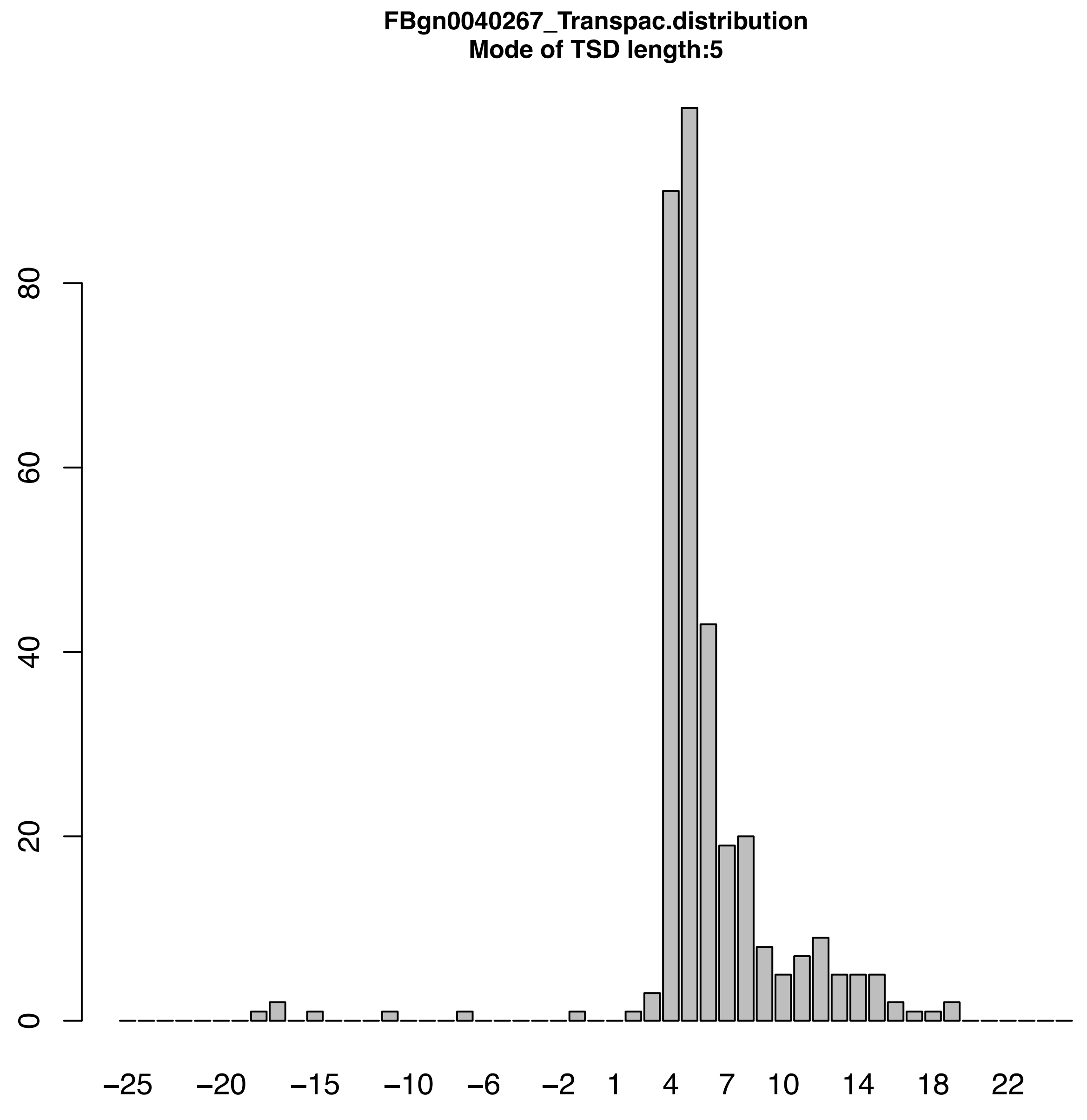
FBgn0040267_Transpac.distribution
Mode of TSD length:5
0
20
40
60
80
−25
−20
−15
−10
−6
−2
1
4
7
10
14
18
22

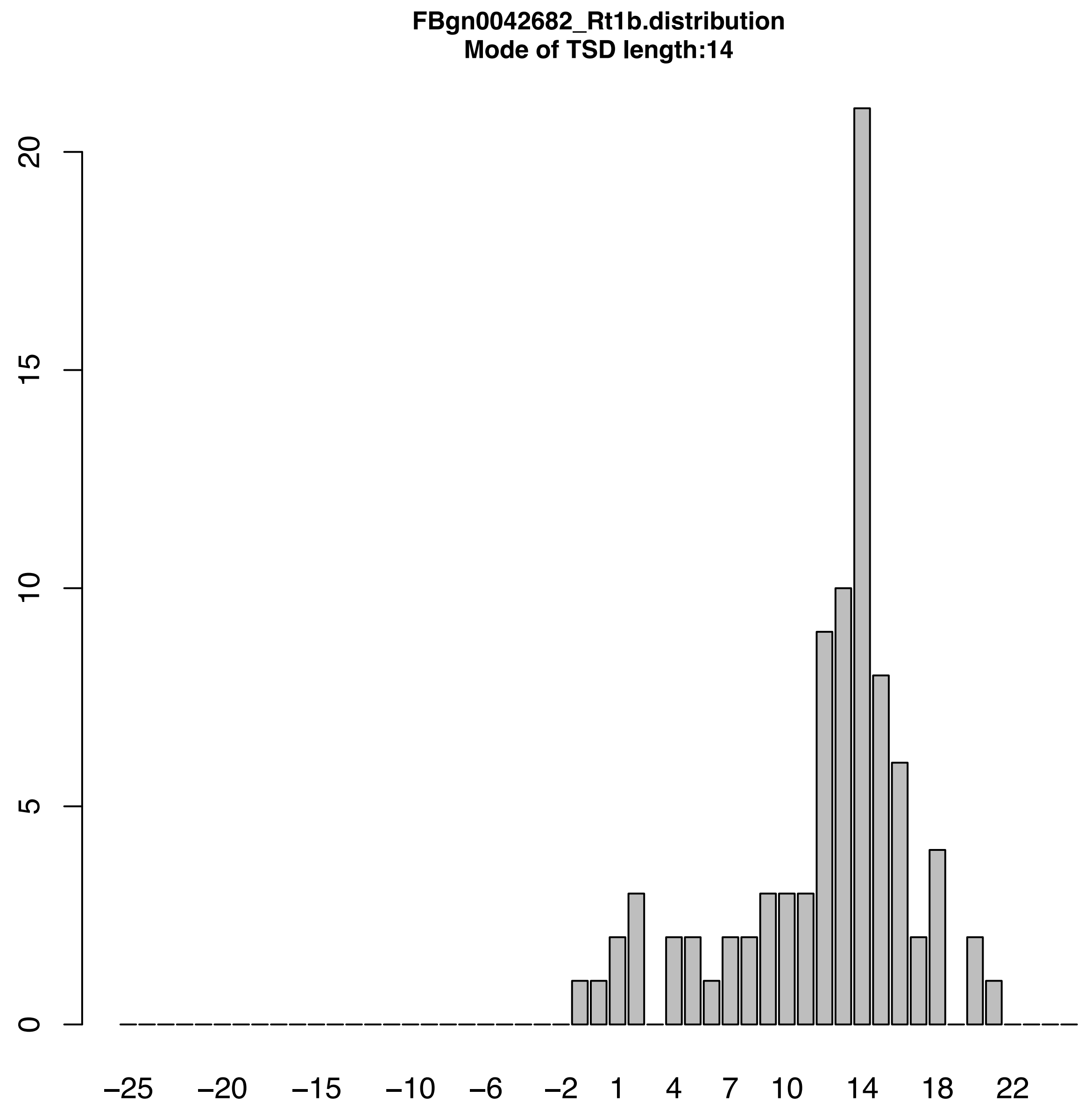
FBgn0042682_Rt1b.distribution
Mode of TSD length:14
0
5
10
15
20
−25
−20
−15
−10
−6
−2
1
4
7
10
14
18
22

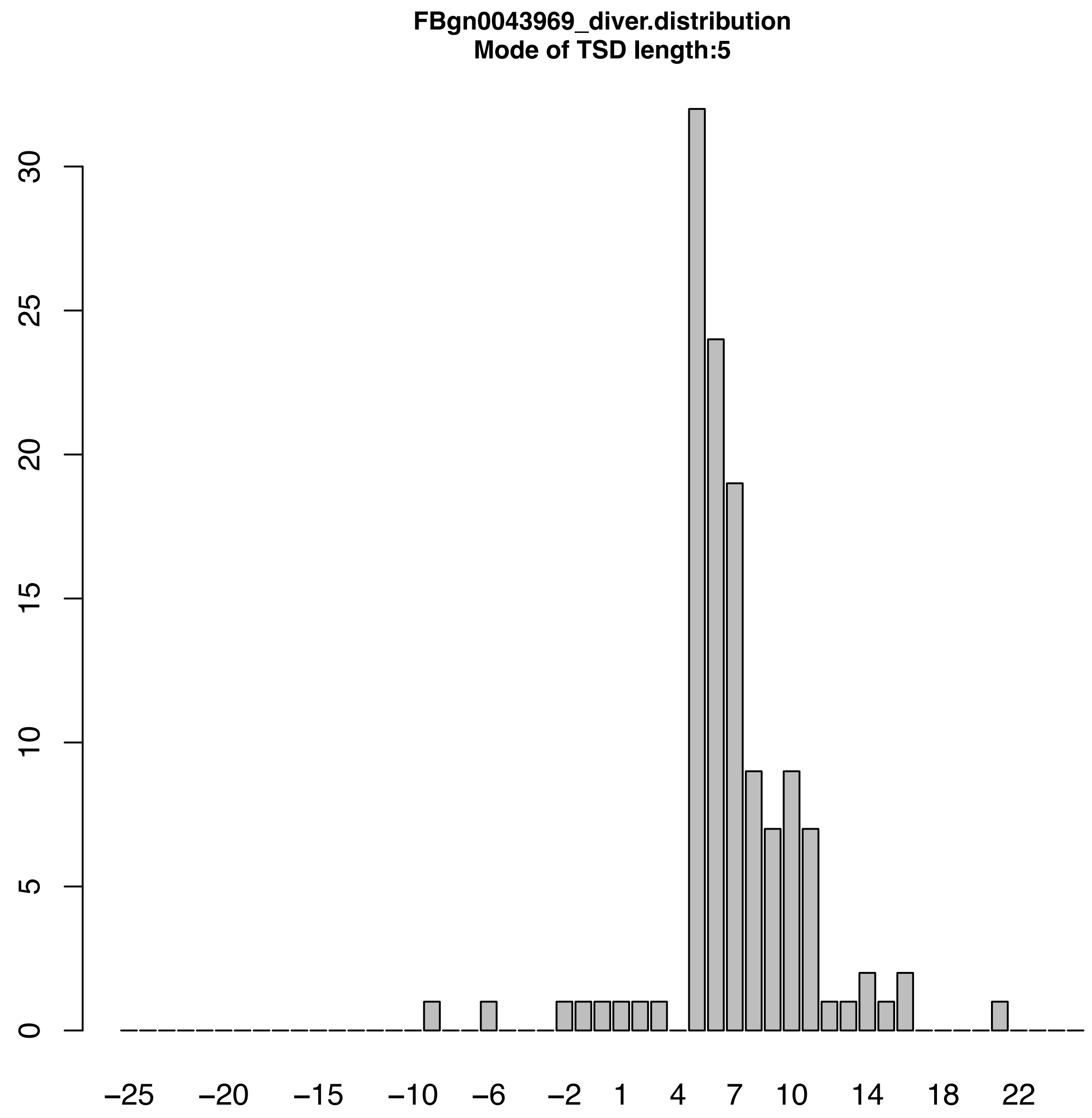
FBgn0043969_diver.distribution
Mode of TSD length:5
0
5
10
15
20
25
30
−25
−20
−15
−10
−6
−2
1
4
7
10
14
18
22

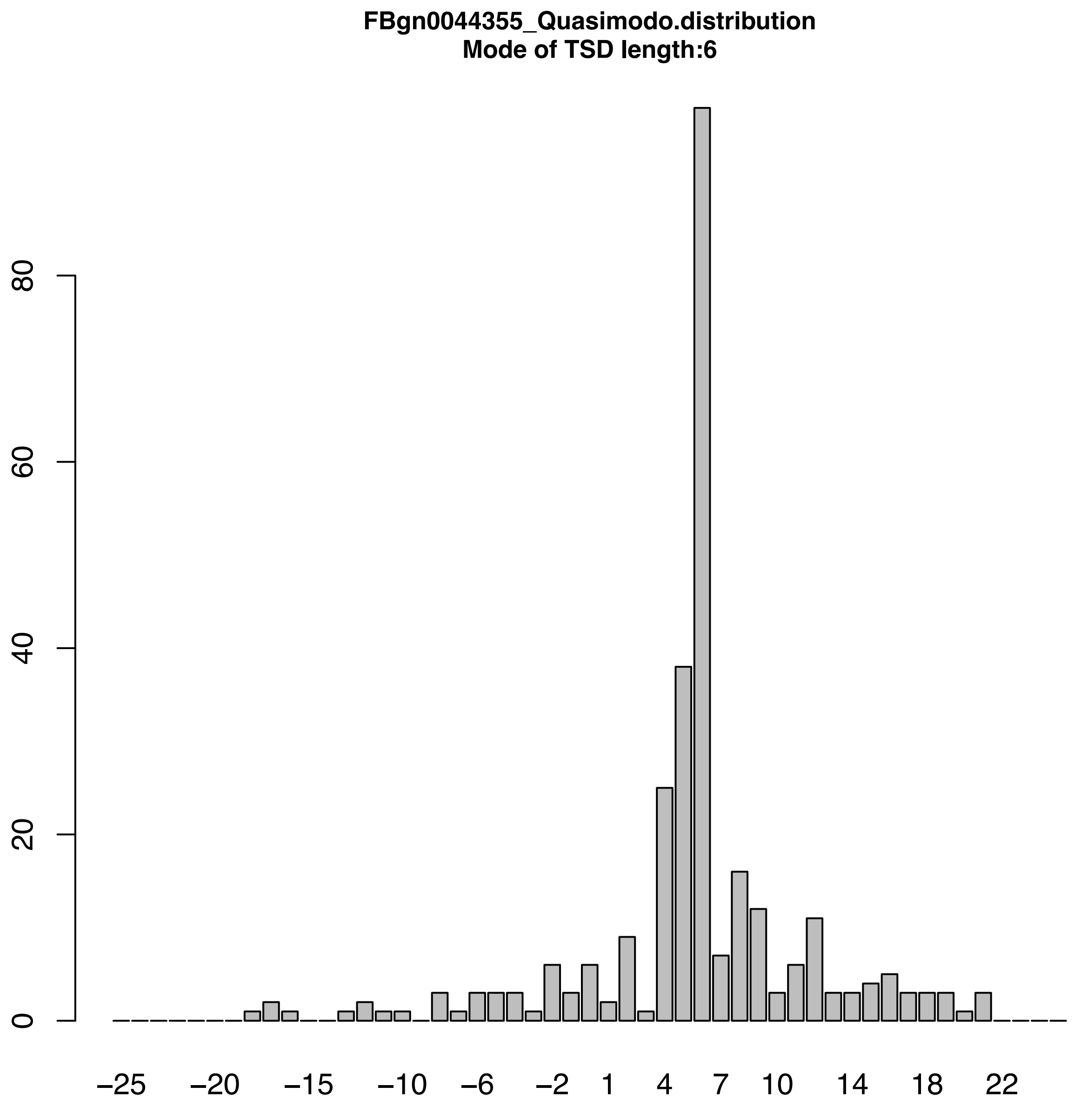
FBgn0044355_Quasimodo.distribution
Mode of TSD length:6
0
20
40
60
80
−25
−20
−15
−10
−6
−2
1
4
7
10
14
18
22

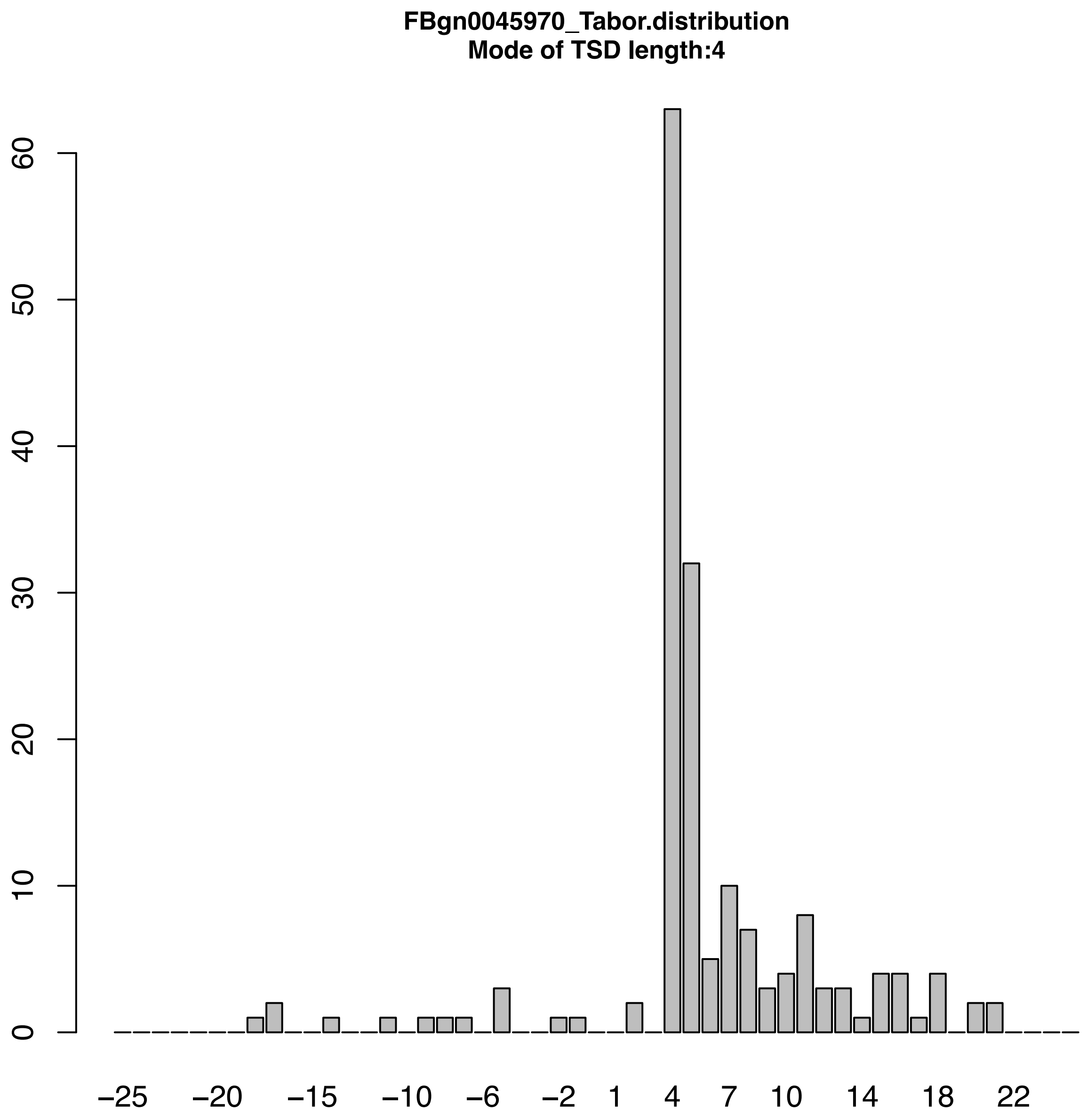
FBgn0045970_Tabor.distribution
Mode of TSD length:4
0
10
20
30
40
50
60
−25
−20
−15
−10
−6
−2
1
4
7
10
14
18
22

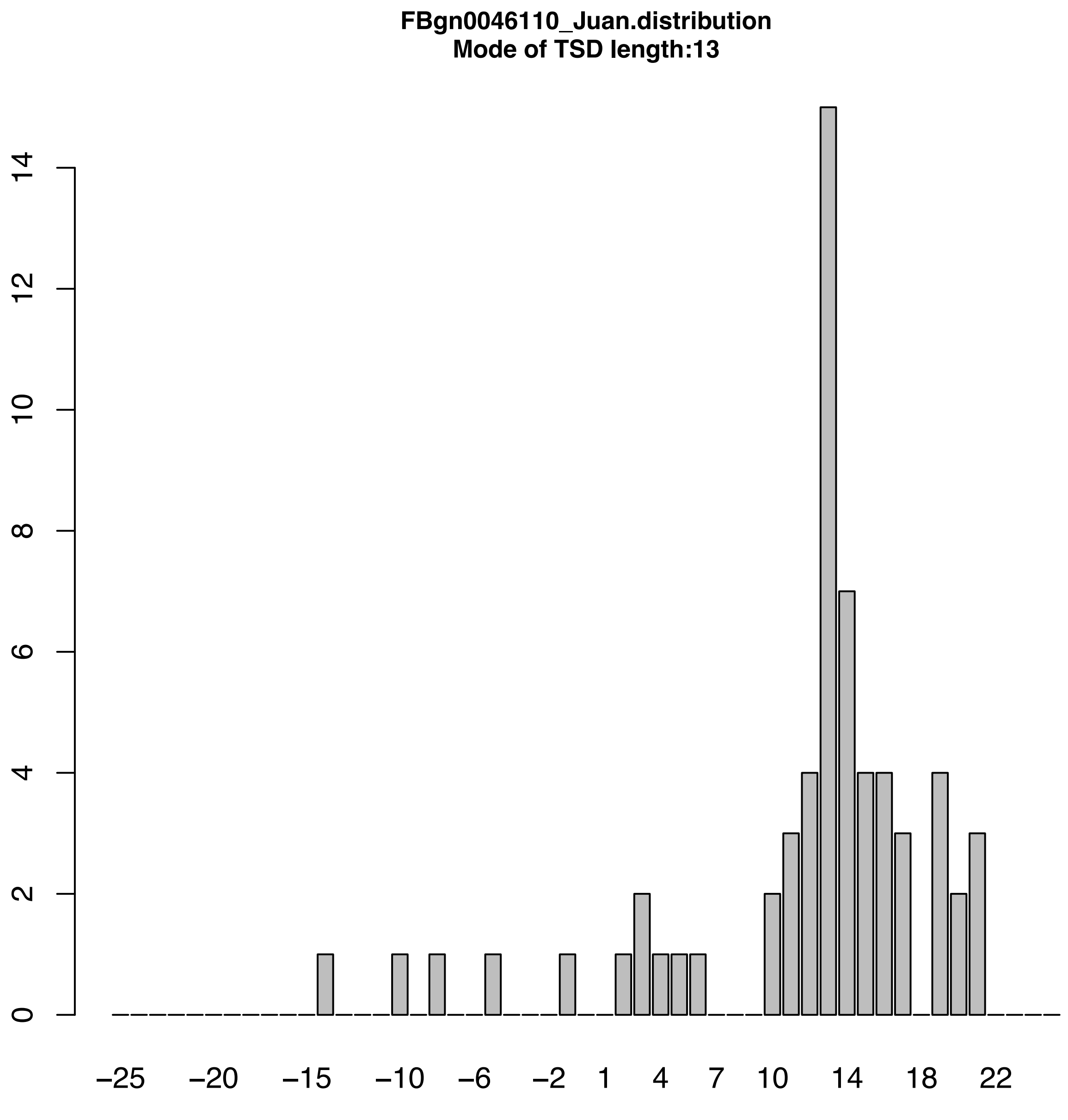
FBgn0046110_Juan.distribution
Mode of TSD length:13
0
2
4
6
8
10
12
14
−25
−20
−15
−10
−6
−2
1
4
7
10
14
18
22

FBgn0061485_rover.distribution
Mode of TSD length:6

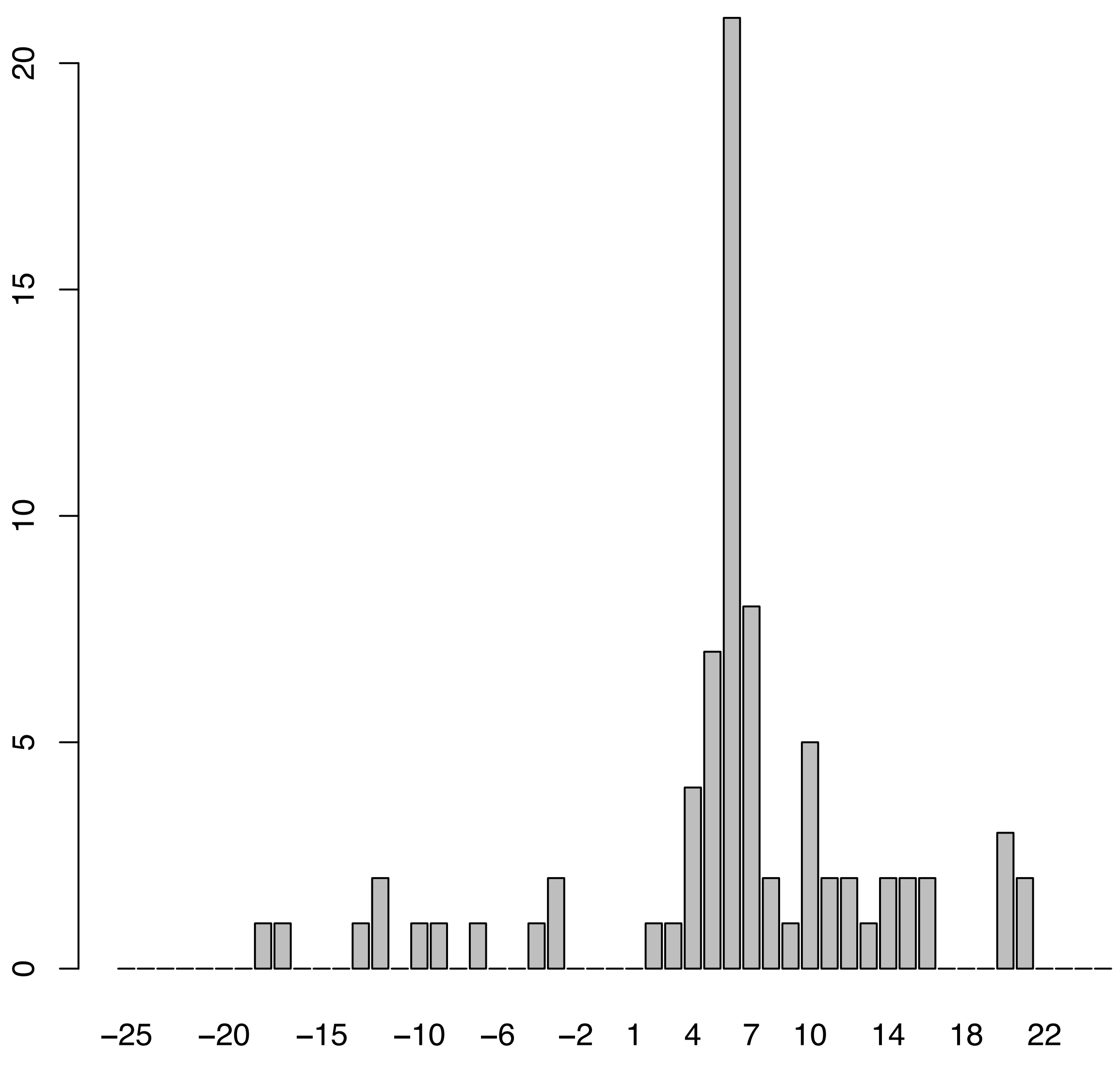

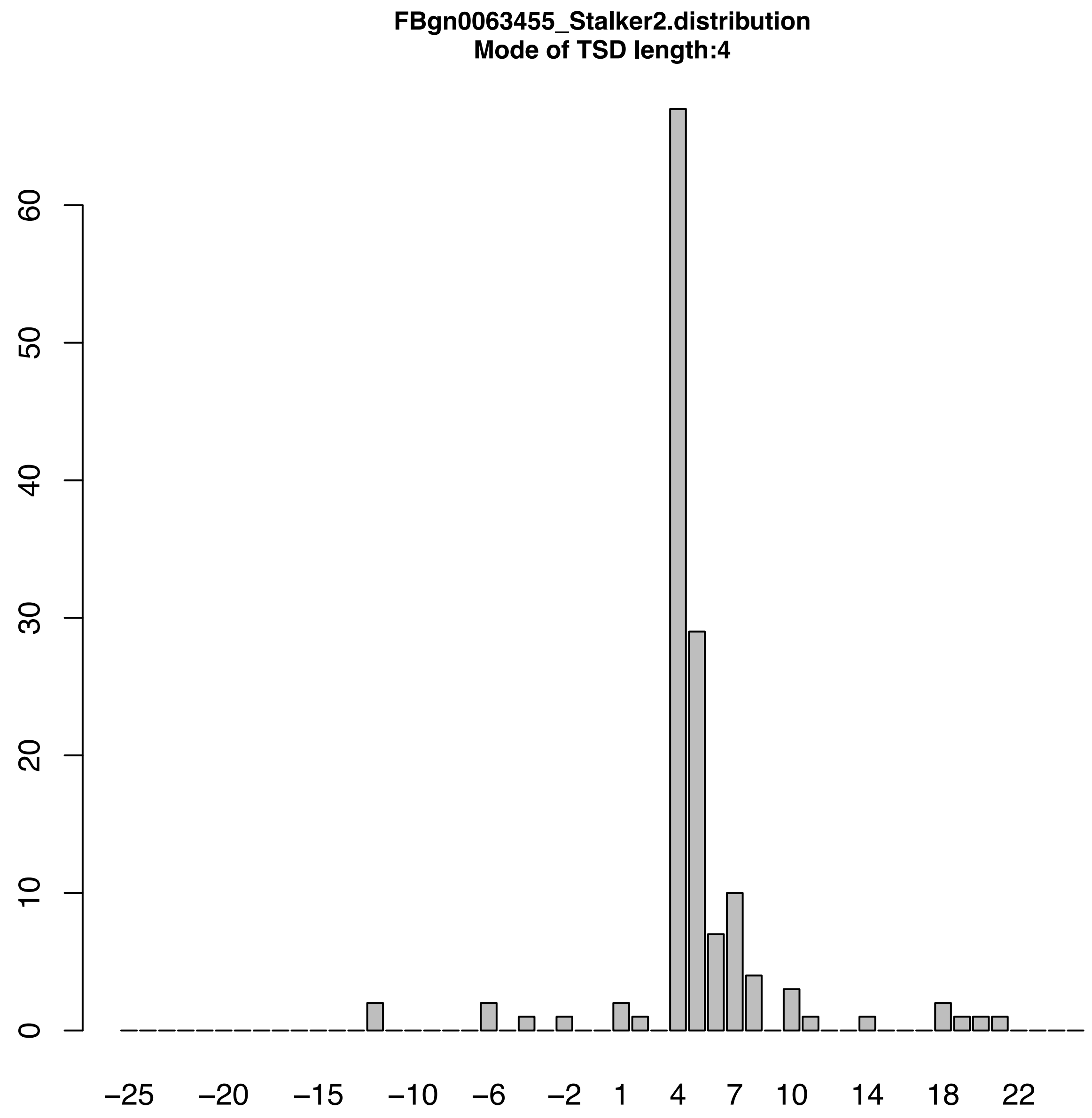

FBgn0063455_Stalker2.distribution
Mode of TSD length:4
0
10
20
30
40
50
60
−25
−20
−15
−10
−6
−2
1
4
7
10
14
18
22

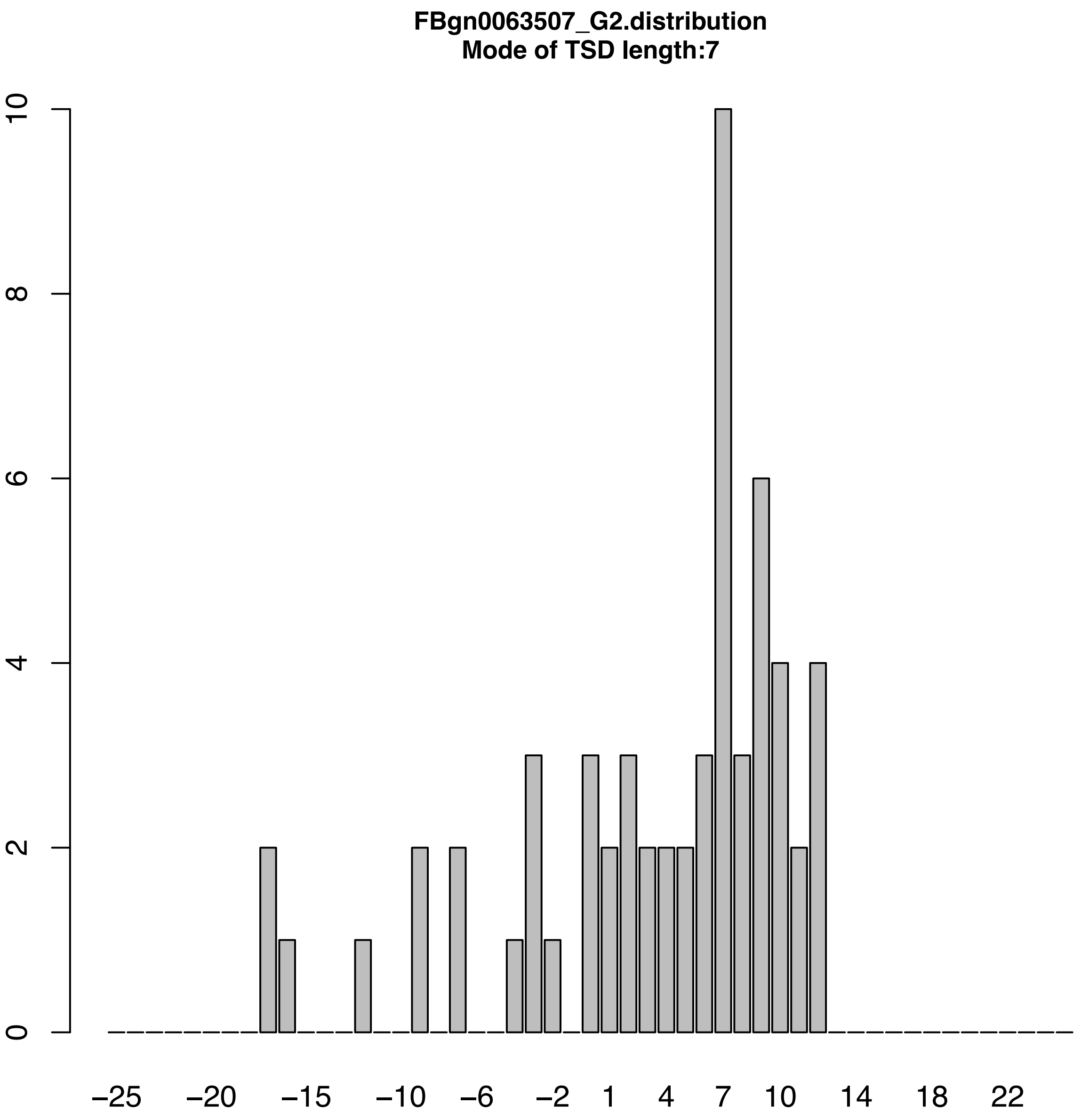
FBgn0063507_G2.distribution
Mode of TSD length:7
0
2
4
6
8
10
−25
−20
−15
−10
−6
−2
1
4
7
10
14
18
22

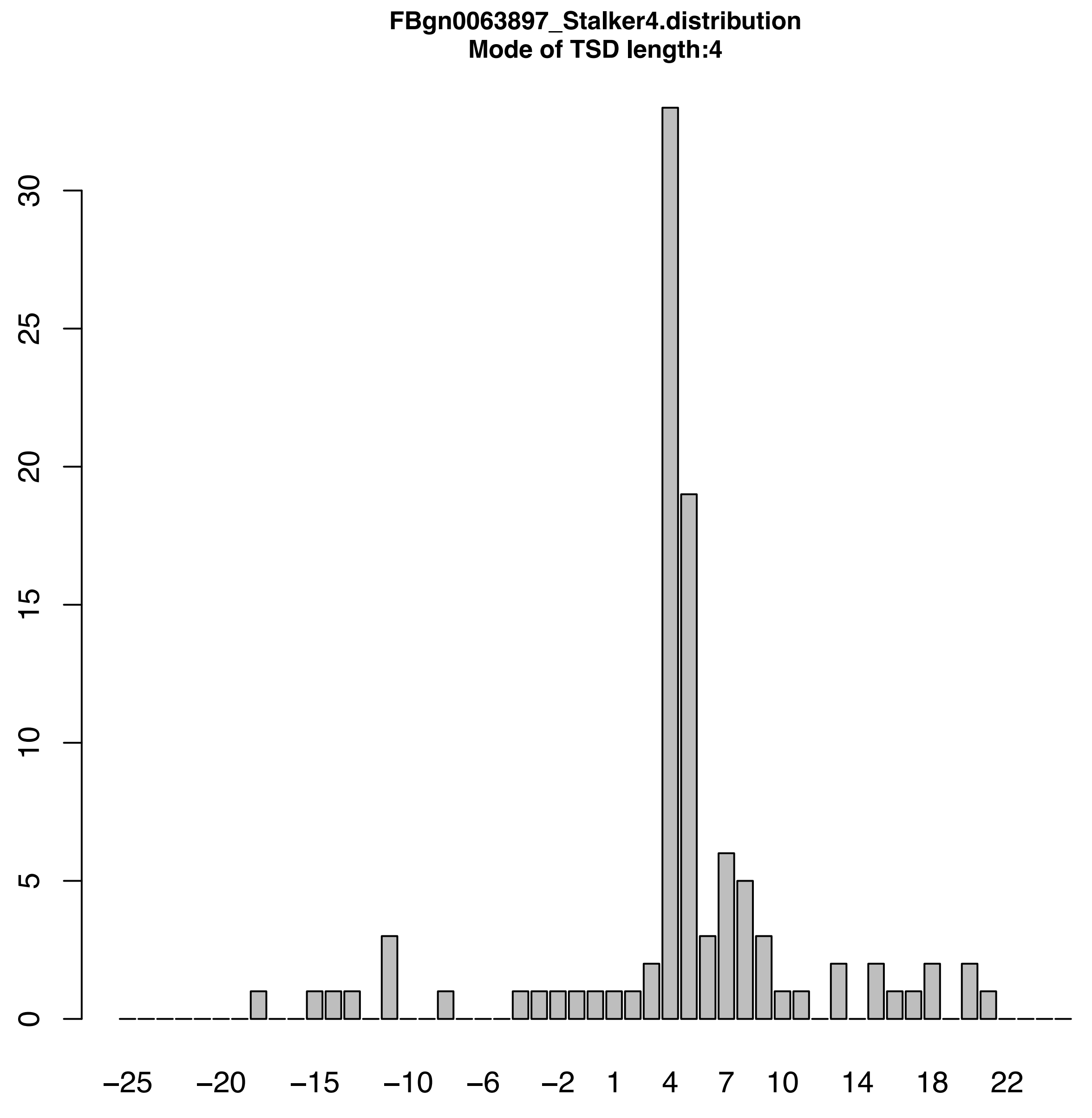
FBgn0063897_Stalker4.distribution
Mode of TSD length:4
0
5
10
15
20
25
30
−25
−20
−15
−10
−6
−2
1
4
7
10
14
18
22

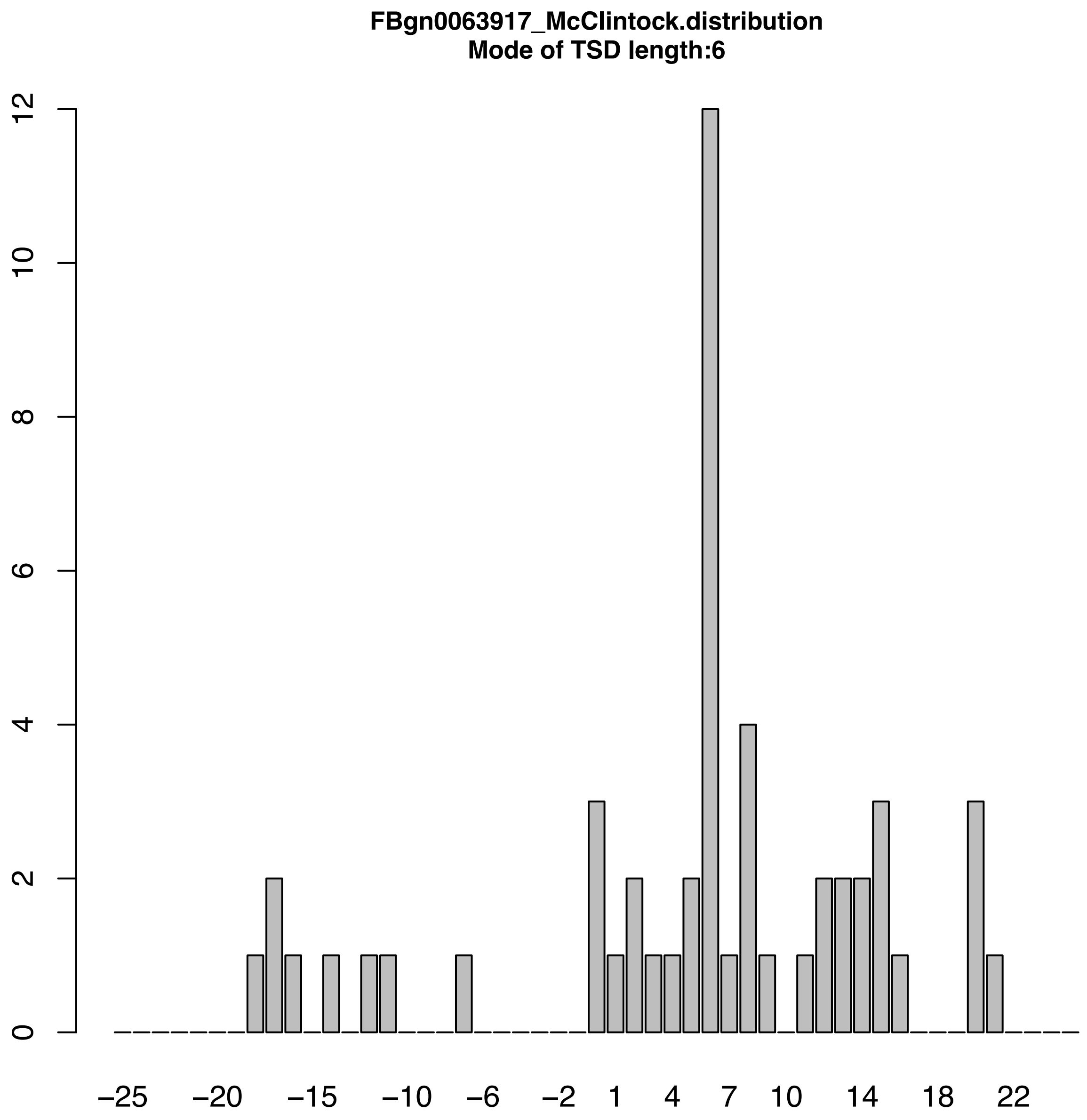
FBgn0063917_McClintock.distribution
Mode of TSD length:6
0
2
4
6
8
10
12
−25
−20
−15
−10
−6
−2
1
4
7
10
14
18
22

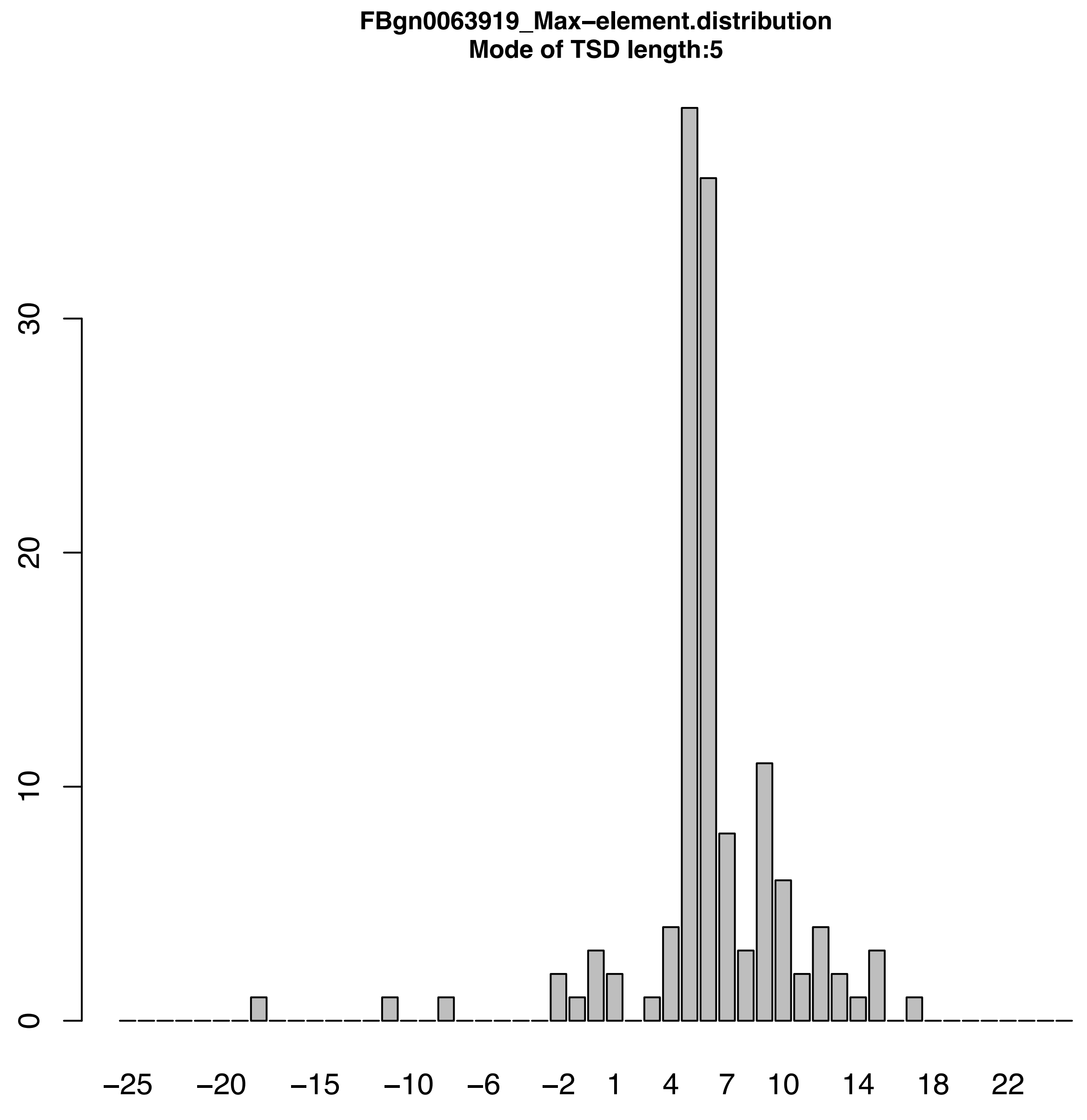

FBgn0063919_Max−element.distribution
Mode of TSD length:5
0
10
20
30
−25
−20
−15
−10
−6
−2
1
4
7
10
14
18
22

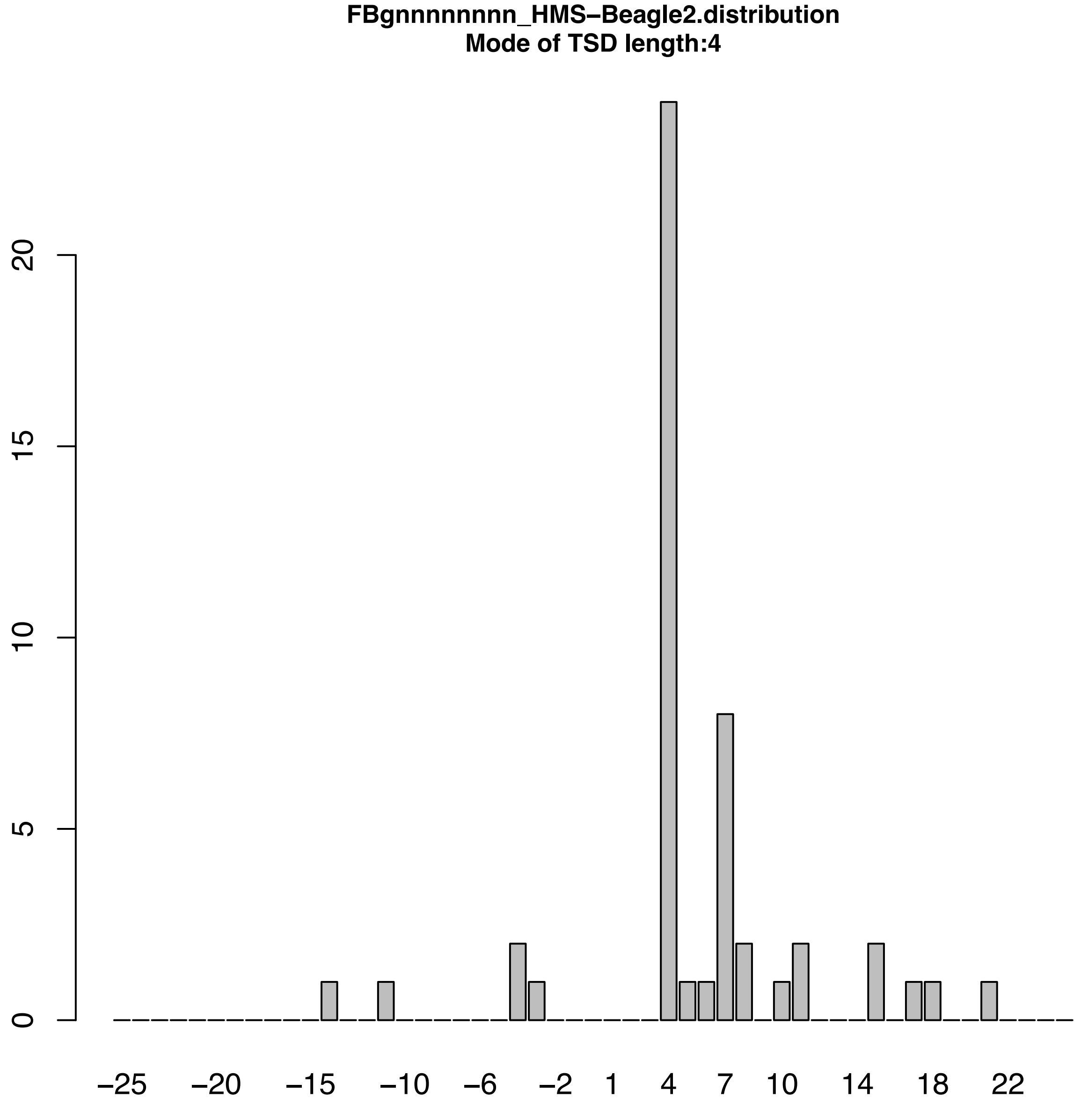
FBgnnnnnnnn_HMS−Beagle2.distribution
Mode of TSD length:4
0
5
10
15
20
−25
−20
−15
−10
−6
−2
1
4
7
10
14
18
22
